# Click chemistry approaches to expand the repertoire of PEG-based fluorinated surfactants for droplet microfluidics†

**DOI:** 10.1039/c8ra01254g

**Published:** 2018-04-06

**Authors:** Randall Scanga, Lucie Chrastecka, Ridhwan Mohammad, Austin Meadows, Phenix-Lan Quan, Eric Brouzes

**Affiliations:** Department of Chemistry, Massachusetts Institute of Technology Cambridge Massachusetts USA randallscanga@yahoo.com; Department of Biomedical Engineering, Stony Brook University Stony Brook New York USA eric.brouzes@stonybrook.edu; Laufer Center for Physical and Quantitative Biology, Stony Brook University Stony Brook New York USA

## Abstract

We report the novel and simplified synthesis of fluorinated surfactants for droplet microfluidics. The range of applications of droplet microfluidics has greatly expanded during the last decade thanks to its ability to manipulate and process tiny amount of sample and reagents at high throughput in independent reactors. A critical component of the technology is the formulation of the immiscible oil phase that contains surfactants to stabilize droplets. The success of droplet microfluidics relies mostly on a single fluorinated formulation that uses a PFPE–PEG triblock surfactant. The synthesis of this surfactant is laborious and requires skills in synthetic chemistry preventing the wider community to explore the synthesis of surfactants with alternate structures. We sought to provide a simplified synthesis for novel PFPE–PEG surfactants based on click chemistry approaches such as copper-catalyzed azide-alkyne cycloaddition (CuAAC) and UV-activated thiol–yne reactions. Our strategy is based on converting a moisture sensitive intermediate typically used in the synthesis of the triblock PFPE–PEG surfactant into a stable and click ready molecule. We successfully combined that fluorinated tail with differently functionalized PEG and glycerol ethoxylate molecules to generate surfactants with diverse structures *via* CuACC and thiol–yne reactions. We report the characterization, biocompatibility and ability to stabilize emulsions of those surfactants, as well as the unique advantages and challenges of the strategy.

## Abstract 2

Click-synthesis of fluorinated surfactants for droplet microfluidics.

## Graphical abstract

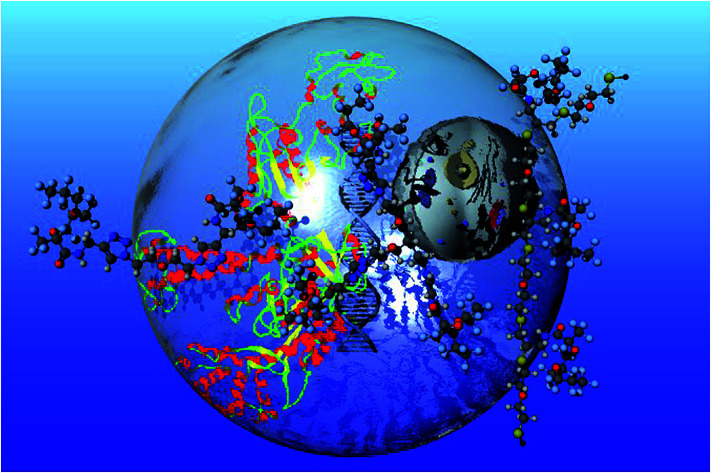

## Introduction

Emulsification plays a critical role in numerous industries such as food processing, or oil industry.^1,2^ Recently, microfluidic approaches for emulsification^3–6^ has enabled a better control of encapsulated samples into monodisperse droplets and the development of platforms for chemical and biological applications. Droplet microfluidics is mostly used to encapsulate and manipulate aqueous samples in droplets with volumes that range from pL to nL into an immiscible phase within microfluidic channels. The integration of different droplet modules such as merging, injection, purification or sorting and their high manipulation rates (up to several 1000's of droplets per second) make it an attractive platform to develop high throughput applications.^7–15^

The critical aspects of the technology are (1) its ability to generate monodisperse droplets, which allows their controlled manipulation in microfluidic channels; (2) the droplet stability that maintains compartmentalization of samples into independent reactors; and (3) its compatibility with a wide array of reactions. Those properties depend on the aqueous, oil and surfactant formulation. Fluorinated surfactant oil formulations have been the most used because they allow very good stability, biocompatibility and limited molecule exchange while requiring low surfactant concentration. An added advantage is the ability to readily merge an emulsion *en masse*, in bulk, by the simply adding a co-surfactant.^9^

The development of novel fluorinated surfactants is still a very active field in the pursuit of formulations for specific or broader applicability.^16,17^ Fluorinated surfactants are based on perfluoropolyethers (PFPE), especially their carboxylated form (Krytox 157 FSH from Dupont, referred as Krytox) that allows chemical coupling to hydrophilic molecules. Its fluorophilicity assures its dissolution into the oil phase and it acts as the surfactant tail. Krytox can be readily used as a surfactant after neutralization with a strong base such as ammonium hydroxide.^11^ While greatly lowering the interfacial tension, and generating highly stable droplets, this ionic surfactant required the use of a triblock poly(ethylene oxide) (PEO)–poly-(propylene oxide) (PPO)–PEO (Pluronic F68) co-surfactant to assure long term viability of encapsulated U937 mammalian cells and it exhibited very rapid molecule exchange when used in conjunction with a mixture of FC 3283 and HFE 7500 fluorinated oils.^11^ Another surfactant with great biological and cellular compatibility was synthesized by appending Krytox tails to a bi-functional dimorpholinophosphate molecule.^12^ Those promising surfactants were however suffering from batch to batch variation.

A breakthrough occurred with the synthesis of a triblock copolymer surfactant composed of a homo-bifunctional polyethylene glycol (PEG) head flanked by Krytox molecules joined *via* an amide linkage.^18^ This surfactant exhibits most of the expected attributes with great stability, even during PCR thermocycling with the addition of a 4-arm PEO–PPO star block copolymer (Tetronic 1307) co-surfactant,^19^ minimal molecule exchange,^11,20^ great compatibility with molecular reactions such as PCR,^9^*in vitro* transcription translation,^21^ and very good cell viability with bacteria and yeast at long timescales (several days) and with mammalian cells at moderate timescales (from a day to a few days depending on the cell type).^22,23^

More recent efforts spanned the development of surfactant stabilized *via* ionic interactions and without synthesis,^24^ the use of nonionic tris(hydroxymethyl)methyl (Tris) as surfactant head,^25^ the use of polyglycerol to replace the PEG head,^8^ as well as a surfactant-free approach that relies on nanoparticles to stabilize droplets by Pickering effect.^26^ Other groups have also designed surfactants for specific applications such as catalytic reaction^27^ or T-cell capture.^28^

Overall, the most used and proven surfactants remain PFPE–PEG based surfactants; however, these generally suffer from lengthy and specialized syntheses^18,29^ that hinders their modification by a larger number of researchers. Our overarching goal is to develop a toolbox usable by the wide microfluidic community to develop novel surfactants to study the structure-function of fluorinated surfactants and expand the scope of droplet microfluidics. In this paper, we investigated the use of click chemistry to simplify the synthesis of PEG-based fluorinated PFPE surfactants and to generate surfactants with novel structures. This approach holds the promise of high reaction efficiency, operational simplicity, and broad substrate scope.

The term click chemistry qualifies chemical reactions that are simple to perform, use readily available reagents, provide high yield, do not generate side-products, can be run in simple solvents and whose purification does not require chromatographic methods.^30^ The archetypical click reaction is the cycloaddition between azides and terminal alkynes, which proceeds rapidly at ambient temperature and pressure when catalyzed by copper.^31,32^ To prevent the oxidation of the catalytic Cu(i) into the inactive Cu(ii), the catalyzer can be generated *in situ* by reduction of copper(ii) acetate monohydrate with sodium ascorbate and can be protected by chelating ligands, also called ligands, such as tris-(benzyltriazolylmethyl)amine (TBTA)^33^ or various phenanthrolines such as neocuproine.^34^

Beyond this hallmark reaction, there have also been numerous reports of new chemistries fulfilling the criteria to be classified as click reactions. Some of the better-known examples include the strain-promoted azide-alkyne cycloaddition (SPAAC), tetrazine ligation, thiol–ene and thiol–yne chemistry. Tetrazine ligation like the SPAAC reaction of cyclooctynes is an additive-free click strategy that is amendable to both bioconjugation and live-cell imaging.^35,36^ In addition, thiol–ene and thiol–yne chemistries are especially effective for construction and modification of polymeric materials. Repetitive thiol–ene reactions can be used to form multifunctional molecules, which are further derived into dendrimers^37^ and thiol–yne reactions easily yield hyperbranched polymers.^38^ In these reactions, a thiyl radical is generated either thermally or in the presence of a photo-initiator such as 2,2-dimethoxy-2-phenylacetophenone (DMPA) to enable its reaction with ene or yne moieties. In the thiol–yne reaction, alkynes react with a single thiol resulting in the formation of vinyl sulfide that subsequently reacts with a second thiol to produce the 1,2-disubstituted adduct.^39^ Thiol–ene chemistry has been used to prepare sulfide linked amphiphilic PFPE–PEG triblock copolymers for antibacterial applications.^40^ Starting from an analogous alkyne derivative, authors have also reported the synthesis of a triazole linked homologue *via* copper-catalyzed azide-alkyne cycloaddition (CuAAC) for application in energy storage.^41^ Interestingly, the strain-promoted copper-free variant SPAAC has been applied to the *in situ* derivatization of interface chemistry for droplet microfluidics after droplet formation.^42^

In this report, we detail the results of our investigation of click based approaches to the synthesis of novel PFPE–PEG surfactants for droplet microfluidics.

## Materials and methods

### Chemicals

All chemicals were used as provided without further purification unless noted otherwise. Tetrahydrofuran, triethylamine, oxalyl chloride, propargyl amine, sodium ascorbate, neocuproine, hexa (ethylene glycol) dithiol, glycerol ethoxylate, PEG 600 diol and hexafluorobenzene were purchased from Sigma Aldrich. Deuterobenzene, deuterated dimethyl sulfoxide and deuterochloroform were purchased from Cambridge Isotope Laboratories (CIL). Copper(ii) acetate was obtained from Acros Organics and 2,2-dimethoxy-2-phenylacetophenone (DMPA) from TCI. TBTA was synthesized following a previously published procedure.^43^ Perfluorotripropylamine (FC 3283), methoxyperfluorobutane (HFE 7100), 2-(trifluoromethyl)-3-ethoxydodecafluorohexane (HFE 7500) and Krytox FSH were obtained from Miller-Stephenson. Acetonitrile (MeCN) was obtained from EMD and hexafluoroisopropanol (HFIP) from Alfa-Aesar. Thiourea was purchased from Chem-Impex International and ethanol, methanol, sodium hydroxide and hydrochloric acid were obtained from Macron Fine Chemicals.

### Synthesis

#### PFPE acid chloride (7)

The procedure for preparation of the PFPE acid chloride adheres to the previously published method with a few minor alterations^18,44^ (see ESI†). Starting with the commercially available end functional PFPE carboxylic acid Krytox FS(H) 157 (DuPont), the corresponding acid chloride is obtained by treatment with oxalyl chloride in refluxing methoxyperfluorobutane (HFE 7100, 3M). The newly generated acid chloride is isolated *in situ* under reduced pressure and redissolved in FC 3283. The resultant solution/suspension is then filtered under inert atmosphere and used without further purification in the subsequent amidation reaction.

^19^F NMR (C_6_D_6_/C_6_F_6_, 282 MHz) *δ* ppm: −80.67 (m, 5F, O–CF(CF_3_)–CF_2_), −81.96 (m, 3F, CF_3_–CF_2_–CF_2_), −82.57 (m, 3F, CF_3_–(CF)–C=O), −125.68 (m, 1F, CF_3_–(CF)–C=O), −130.48 (s, 2F, CF_3_–CF_2_–CF_2_), −144.87 (m, 1F, O–CF(CF_3_)–CF_2_).

#### PFPE amides (8 and 9)

The amidation reaction is performed in a biphasic solvent system comprised of FC 3283 and THF in the presence of TEA which serves as an HCl scavenger (see ESI† and Holtze *et al.*^18^). The desired amine coupling partner dissolved in THF is added dropwise over *ca.* 30 min to the fluorocarbon phase resulting in the formation of a fine emulsion. After *ca.* 24 h the crude reaction mixture is isolated *via* rotary evaporation and once again redissolved in FC 3283 and filtered to obtain a clear, viscous, yellow oil.

Propargyl derivative (8) ^1^H NMR (C_6_D_6_/C_6_F_6_, 300 MHz) *δ* ppm: 6.58 (br, 1H, C(O)–NH–CH_2_), 4.12 (m, 2H, O–CH_2_–C≡), 2.18 (t, 1H, –C≡H).

^19^F NMR (C_6_D_6_/C_6_F_6_, 282 MHz) *δ* ppm: −80.74 (m, 5F, O–CF(CF_3_)–CF_2_), −82.54 (m, 3F, CF_3_–CF_2_–CF_2_), −83.72 (m, 3F, CF_3_–(CF)–C=O), −130.47 (s, 2F, CF_3_–CF_2_–CF_2_), −133.39 (m, 1F, CF_3_–(CF)–C=O), −144.86 (m, 1F, O–CF(CF_3_)–CF_2_).

### CuAAC reactions

#### PFPE–PEG triazole linked triblock copolymer (12)

3.0 mL of methoxyperfluorobutane (HFE 7100) was added to 3.0 g (0.51 mmol) of PFPE-propargyl derivative and sonicated to obtain a clear, homogeneous solution. A solution of 164 mg (0.27 mmol) of PEG 600 diazide in 1.5 mL of MeOH was prepared and sonicated. Next, 0.0102 g (0.051 mmol, 10 mol%) Cu(OAc)_2_ and 17 mg of neocuproine (0.0815 mmol, 16 mol%) were added to the previously generated PEG 600 diazide/MeOH solution and further sonicated.

20.2 mg (0.102 mmol) of sodium ascorbate was then combined with 1.5 mL of DI H_2_O, mixed and transferred to the PEG 600 diazide/Cu(OAc)_2_/neocuproine/MeOH solution. Finally, the combined solution was added to the PFPE-propargyl derivative/HFE 7100 solution in a single portion, and stirred at 1200 rpm with a 1′′ × 1/2′′ PTFE coated stir bar at room temperature (r.t.) for *ca.* 1 h before heating to 60 °C. After *ca.* 48 h, 6.0 mL of MeOH was added to the crude reaction mixture and stirred vigorously for *ca.* 5 min. The resulting destabilized emulsion was left to stand until complete phase separation was noted. The fluorocarbon phase was then extracted and dried over MgSO_4_ under stirring for *ca.* 30 min before filtering through a 0.2 μm PFTE syringe filter. Finally, solvent was removed under reduced pressure.

^1^H NMR (C_6_D_6_, 300 MHz) *δ* ppm: 8.49 (br, 1H, C(O)–NH–CH_2_), 7.9 (s, 1H, –CH_2_–CN=CH–), 4.67 (br, 2H, –NH–CH_2_–CN=), 4.55 (br, 2H, –N–CH_2_–CH_2_–), 3.9 (br, 2H, –N–CH_2_–CH_2_–), 3.6 (br, 2H, –O–CH_2_–CH_2_–). FTIR *ν* cm^−1^: 2874 (–C–H), 1721 (C(NH)=O), 1533 (amide II), 1306–1130 (C–F, C–O, overlapping).

#### PFPE–PEG triazole linked tetrablock copolymer (14)

A solution of 1.5 mL 2-(trifluoromethyl)-3-ethoxydodecafluorohexane (HFE 7500) and 1.5 g (0.254 mmol, 3.0 eq.) of PFPE-propargyl derivative was prepared. 95.6 mg (0.0889 mmol, 1.05 eq.) of glycerol ethoxylate triazide and 0.975 mL of MeCN was prepared and sonicated. Next, 0.00761 g (0.0381 mmol, 15 mol%) Cu(OAc)_2_ and 40.8 mg of tris-(benzyltriazolylmethyl)amine (TBTA) (0.0815 mmol, 16 mol%) were added to the previously generated glycerol ethoxylate triazide/MeCN solution and sonicated. 17.6 mg (0.0889 mmol) of sodium ascorbate was then combined with 0.525 mL of DI H_2_O, mixed and transferred to the glycerol ethoxylate triazide/Cu(OAc)_2_/TBTA/MeCN solution. Finally, the combined solution was added to the PFPE-propargyl derivative/HFE 7500 solution in a single portion, and stirred at 1200 rpm with a 1′′ × 1/2′′ PTFE coated stir bar at r.t. for *ca.* 1 h before heating to 80 °C. After *ca.* 48 h, 6.0 mL of MeOH was added to the crude reaction mixture and further stirred for *ca.* 5 min. Stirring was discontinued and the destabilized emulsion was left to phase separate. The fluorocarbon phase was then isolated and dried over MgSO_4_ under stirring for *ca.* 30 min before filtering through a 0.2 μm PFTE syringe filter. Finally, solvent was removed under reduced pressure at elevated temperature (70–80 °C).

^1^H NMR (C_6_D_6_, 300 MHz) *δ* ppm: 9.15 (br, 1H, C(O)–NH–CH_2_), 7.97 (br, 1H, –CH_2_–CN=CH–), 4.57 (br, 2H, –N–CH_2_–CH_2_=), 3.54 (br, 2H, –O–CH_2_–CH_2_–).

### Thiol–yne reactions

#### Brush-like PFPE–PEG polymer (13)

2.0 g (0.34 mmol, 1 equiv.) of PFPE-propargyl derivative was dissolved in 7 mL of methoxyperfluorobutane. Next, 106.8 mg (96 μL, 0.34 mmol, 1.0 equiv.) of hexa(ethylene glycol)dithiol and 17.6 mg (0.068 mmol, 0.2 equiv.) of 2,2-dimethoxy-2-phenylacetophenone DMPA were combined and immediately dissolved in 3.5 mL of MeOH. The DMPA/thiol/MeOH solution was then transferred to the previously generated PFPE-propargyl derivative/HFE 7100 solution and the vial sealed with a septum. The sealed vessel was then flushed under positive pressure of N_2_ and irradiated at 365 nm while stirring at *ca.* 1200 rpm, at r.t. overnight. The following day the crude reaction mixture was combined with an equivalent volume of MeOH (10.5 mL) and allowed to stand until complete phase separation was noted. The fluorous phase was then isolated and directly evaporated *in vacuo* to obtain a clear, colorless oil.

^1^H NMR (C_6_D_6_, 300 MHz) *δ* ppm: 8.35 (br, 1H, C(O)–NH–CH_2_), 7.42 (br, 1H, –NH–CH_2_–CH=), 6.24 (br, 1H, –CH=CH–S), 5.68 (br, 1H, –CH=CH–S), 3.67 (br, 2H, –O–CH_2_–CH_2_–), 3.02 (1H, –CH–CH_2_–S), 2.84 (1H, –CH–CH_2_–S).

#### Hyperbranched PFPE–PEG (15)

2.0 g (0.339 mmol, 2 equiv.) of PFPE-propargyl derivative was dissolved in 7 mL of methoxyperfluorobutane. Next, 314 mg (0.285 mmol, 3.78 equiv.*) of glycerol ethoxylate trithiol and 17.4 mg (0.0678 mmol, 0.2 equiv.) of 2,2-dimethoxy-2-phenylacetophenone DMPA were combined and immediately dissolved in 3.5 mL of MeOH. The DMPA/thiol/MeOH solution was then transferred to the previously generated PFPE-propargyl derivative/HFE 7100 solution and the vial sealed with a septum. The sealed vessel was then thoroughly flushed with N_2_ and irradiated at 365 nm under vigorous stirring, at r.t. overnight. After *ca.* 24 h the crude reaction mixture was combined with an equivalent volume of MeOH (10.5 mL) and the phases were allowed to separate. Finally, the fluorous phase was extracted and evaporated *in vacuo* to obtain a clear, colorless oil. *Stoichiometry adjusted to account for the presence of disulfide.

^1^H NMR (C_6_D_6_, 300 MHz) *δ* ppm: 8.68 (br, 1H, C(O)–NH–CH_2_), 7.84 (br, 1H, –NH–CH_2_–CH=), 6.38 (br, 1H, –CH=CH–S), 5.68 (br, 1H, –CH=CH–S), 3.75 (2H, –O–CH_2_–CH_2_–), 3.09 (1H, –CH–CH_2_–S), 2.9 (2H, –CH–CH_2_–S).

### Molecules obtained and chemical characterization

#### Measurements

##### Nuclear magnetic resonance (NMR)

^1^H NMR and ^19^F NMR were performed at 300 MHz and 282 MHz respectively on a Varian Mercury system equipped with a H/F/X PFG probe. ^19^F NMR data processing (baseline correction, phasing, *etc.*) was performed in NUTS from Acorn NMR Inc. Final plotting, analysis (assignments, peak picking, integration) were performed in MestreNova (Mnova) from Mestrelab Research. PEG samples were prepared in CDCl_3_*ca.* 30 mg mL^−1^ and PFPE samples *ca.* 300 mg mL^−1^ in either 90/10 C_6_F_6_/C_6_D_6_ or FC 3283 with C_6_D_6_ coaxial insert.

##### Fourier-transform infrared spectroscopy (FT-IR)

IR was collected on neat, thin films deposited on NaCl using a Perkin-Elmer Spectrum 100 FT-IR spectrometer using default settings. Data processing and plotting was performed in Essential FTIR.

##### Pendent drop method

The pendent drop method was implemented on a custom goniometer comprising a backlight, a syringe and its holder, a horizontally mounted camera (Sony XCV60) on an inspection zoom monocular microscope (Amscope, H800-CL). The recording system was controlled by a program developed under Labview (National Instrument).

Drops of oil and surfactant were generated manually in de-ionized water contained in a spectrometer cuvette using a disposable 1 mL plastic syringe fitted with a 1.08 mm diameter 5 μL glass capillary (Drummond Scientific Company, 2-000-005) to minimize wetting effects at the tip. The drop profile was extracted with ImageJ and interpreted into interfacial energy using the plug-in “Pendent drop”^45^ after spatial calibration of the system. Each curve fitting was validated before the interfacial energy was recorded. The kinetics of the interfacial energy was assessed first to obtain the equilibrium time for each surfactant at 3% wt in HFE 7500 (ESI Fig. 1, Table 2†). Each measurement was performed after this equilibrium was reached, which should apply to the whole range of concentrations characterized.^46^ The equilibrium timescales were obtained by fitting the evolution of the interfacial energy over time with an exponential decay. Each dilution series was prepared beforehand starting with the highest surfactant concentration in pure HFE 7500. Each dilution series was measured three times in increasing concentration, and the interfacial energy between pure HFE 7500 and water was measured at the end of each series to check for contamination (ESI Fig. 2†).

### Emulsion generation and stability

The stability of emulsions generated with different formulations was first tested after encapsulation of D-PBS into the fluorinated phase using a simple PDMS-glass hybrid droplet generator manufactured by soft-lithography.^47,48^ The nozzle dimension was 40 μm wide, the oil inlets 30 μm wide and their depths 50 μm. The fluids were actuated with a custom-built pressure system controlled with a software developed under Labview environment (National Instruments). The microfluidic devices were mounted onto an inverted microscope (Nikon, Diaphot TMD) equipped with a monochrome camera (Sony, XCV60). Using image-based method developed under ImageJ, we reported the volume fraction and the droplet size as a function of pressure sets. We compared the synthesized surfactants to the PFPE–PEG surfactant using those parameters (ESI Fig. 3†). We collected the emulsions generated at 3 psi oil −3 psi D-PBS into 200 μL PCR microtube, and incubated them at room temperature for several weeks (ESI Fig. 4†). Emulsion stability was assessed quantitatively by analyzing the size of individual droplets (*n* > 800) after re-injecting an aliquot of the emulsion into a large channel (width from 300 to 600 μm wide and 100 μm deep) where droplets assumed a spherical shape.

### Cloning and PCR reaction

A 250 bp fragment of the proto-oncogene KRAS mRNA (accession number # NG_007524, nt 148-397) was cloned into the PCR2.1-TOPO TA cloning vector (plasmid KRAS-PCR2.1) (Eurofins Genomics). A 150 bp KRAS fragment was amplified from plasmid KRAS-PCR2.1 using Platinum PCR SuperMix High Fidelity kit (Invitrogen) and primers KRAS-FWD-(5′-GAGTGCCTTGACGATACAGCT) and KRAS-REV (5′-GCACTGTACTCCTCTTGACC). The resulting PCR product was sequenced to verify the specificity of the reaction. PCR reactions were performed on a SimpliAmp thermocycler (Applied Biosystems) with 25 thermocycles with the following temperature pattern: 95 °C, 55 °C and 68 °C each for 30 seconds. Tetronic 1307 (BASF, stock at 5% weight in water) was added at a final concentration of 1% weight in PCR reactions when used.

### In-droplet PCR experiment

Droplets were generated similarly as described for the encapsulation of D-PBS. The PCR master mix and emulsions were maintained at 4 °C (digital dry bath, Torrey Pines Scientific) during droplet generation. Emulsions were collected into PCR tubes. The majority of the fluorous phase was removed before thermocycling.

The emulsion stability after PCR was first reported qualitatively, after visual inspection, following a grading system where a macroscopically stable emulsion is 1, an apparent intermediate stability as judged by the presence of a white gradients that indicates the presence of large droplets at the top of the emulsion is 0.5, and a massive collapse defined as a clear portion of the emulsion being a single phase (even if the remaining is still an apparent emulsion) is 0 (ESI Fig. 5†). Specific emulsions were assessed quantitatively following the protocol described for D-PBS emulsions by analyzing an aliquot with the image-based protocol.

DNA amplification was verified by electrophoresis on a 2% agarose electrophoresis gel (E-gel electrophoresis system, Thermofisher) after emulsions were broken *en masse* using perfluoro-octanol (Alfa-Aesar) (1 : 1 volume ratio).

### In-droplet helicase dependent isothermal amplification (HDA)

HDA isothermal DNA amplification was performed using the IsoAmp Universal tHDA kit (biohelix), plasmid KRAS-PCR2.1 and the KRAS-FWD and KRAS-REV primers. The HDA reaction was prepared before its encapsulation with the different surfactants at 3% wt in HFE 7500. The emulsions were collected into PCR tubes and the oil phase was removed before incubation at 65 °C for 90 min. The emulsions were broken *en masse* using an equal volume of perfluoro-octanol and the 150 bp amplification product was observed by electrophoresis on a 2% agarose gel (ESI Fig. 6†). Emulsion stability was performed quantitatively as described above.

### Molecular transport test

To test for molecular transport from the aqueous phase to the fluorinated phase,^11,20,49,50^ we used the partitioning of Sytox orange (Thermo Fisher), a DNA staining dye. The dye gives a pink coloration to the phase it partitions into. Molecular transport would be indicated by a pink coloration of the oil phase (bottom phase, ESI Fig. 7†). Surfactants are dissolved at 3% wt in HFE 7500 and used in vortex generated emulsions containing Sytox orange, before being briefly centrifuged with a benchtop centrifuge. We used the PFPE–PEG and the ionic surfactant (ammonium Krytox) as controls for absence and presence of molecular transport respectively.

### Overlay experiments for biocompatibility of surfactants with CHO cells

A series of 15 (3 × 5) 3 mm diameter microwells were made of a PDMS slab bonded to a glass slide after activation by oxygen plasma. This slab was surrounded by another deeper piece of PDMS to create a pool chamber (ESI Fig. 10†). This allowed to run overlay experiments with as low as 10 μL of each oil formulation per condition. The oil phases were first dispensed in their respective wells before cell seeding at a density of 12 000 cells per microwell. The microwell slab was then submerged by cell medium to avoid evaporation. The device was mounted into a custom-designed environmental chamber to maintain a water-saturated 5% CO_2_ atmosphere at a constant 37 °C temperature. Microwells were imaged overtime using an automated Nikon (Eclipse Ti) microscope. The CHO cell line expressing nuclear-tagged EGFP under the control of the constitutive CMV promoter^51^ was graciously donated by the authors (Dr Balazsi). We used these cells to probe cell viability overtime and biocompatibility of the fluorinated surfactants by click approaches. Each surfactant was tested diluted 3% in mass into HFE 7500, controls included conditions without a fluorinated phase, HFE7500 oil only as well as the ammonium Krytox surfactant (ionic surfactant) that is known to induce rapid cell death.^11^ CHO-GFP cells were incubated into F-12 medium complemented with 10% fetal bovine serum and streptomycin antibiotics under 5% CO_2_ atmosphere at 37 °C. Cells were tested negative for mycoplasma using a mycoplasma PCR kit (e-Myco Mycoplasma PCR Detection Kit, iNtRON Biotechnology).

## Results and discussion

### Strategy

One shortcoming of existing non-ionic surfactant systems is their relatively lengthy synthesis, which requires some skills in the art of synthetic chemistry and necessitates the use of specialized techniques and equipment. Our strategy is based on converting a moisture sensitive intermediate typically used in the synthesis of the triblock PFPE–PEG surfactant into a stable and click ready molecule that can easily be coupled with different surfactant heads.

**Fig. 1:**
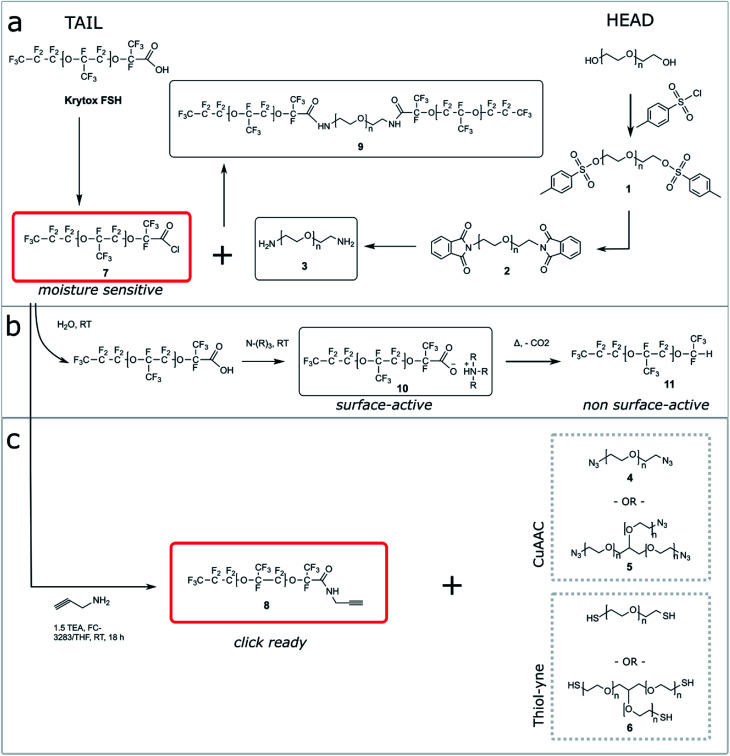
Simplified synthesis of fluorinated surfactants by click approaches. (a) PFPE–PEG surfactant usually results from the combination of the reactive but moisture-sensitive acid chloride of Krytox (7) with a PEG diamine (3). (b) The acid chloride can easily react with water and decompose into surface-active salts (10). (c) Our strategy consists in creating a stable click ready Krytox propargyl amine intermediate (8) (from the acid chloride of Krytox) that can be easily reacted with azide or thiol functionalized PEG or glycerol ethoxylate.

**Fig. 2:**
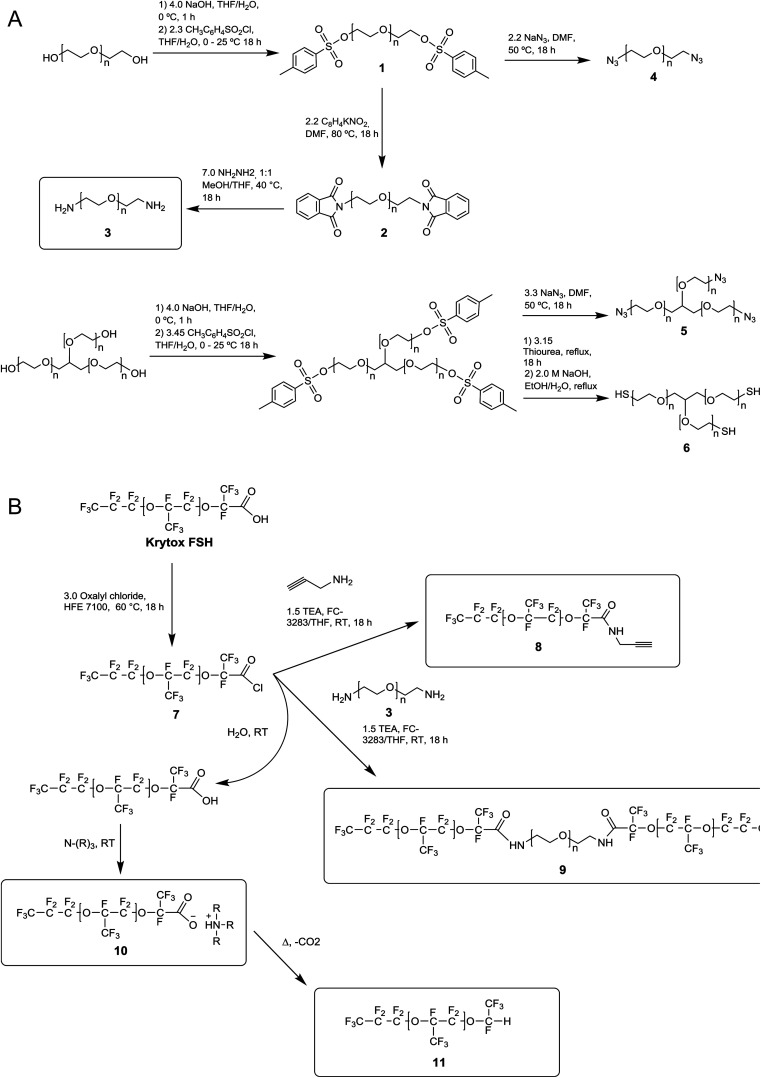
Detailed reactions and experimental conditions of for the surfactants heads (A) and tail (B). Treatment of PFPE carboxylic acid with 3.0 equiv. (COCl)_2_ in refluxing HFE 7100 yields the reactive PFPE acid chloride intermediate (7). Amidation of (7) is performed in ∼3 : 1 FC 3283 : THF in the presence of 1.5 equiv. TEA with the desired amine coupling partner at RT to obtain (8, 9). During/or in preparation of the amidation reaction, reactive intermediate (7) is subject to hydrolysis reforming the parent carboxylic acid. The resultant carboxylic acid then undergoes acid–base reaction with available amines to form ammonium salts (10). Ammonium salts (10) can also undergo decarboxylation to PFPE-HEC (11) on heating.

The synthesis of the amide-linked PFPE–PEG surfactant (9) begins with conversion of commercial PEG 600 diol to diamine by means of tosylation (1) followed by Gabriel amine synthesis (2,3) (Fig. 1a–2a). Commercial PFPE carboxylic acid (Krytox 157 FSH, referred to as Krytox) is then converted to the corresponding acid chloride by treatment with a suitable chlorinating agent (Fig. 1a–2b). Finally, the acid chloride 7 is reacted with lyophilized PEG 600 diamine 3 to obtain 9 at a purity of ∼95% (∼80% purity is otherwise obtained^18^); appropriate steps must be taken to exclude water at other critical stages of the reaction as well. As such, Schlenk line operations and other air/moisture-free manipulations must be employed to obtain analytically pure materials. When water is present, the highly electron deficient fluorinated acid chloride 7 is rapidly hydrolyzed to the parent carboxylic acid. This carboxylic acid then undergoes further reaction resulting in the production of carboxylate salts 10, which are themselves prone to decarboxylation to 11.^52^ The thermal stability of **10** is dependent upon the identity of the counterbalancing cation (Fig. 1b and 2b). This decarboxylation is evidenced and can be monitored by the appearance and/or intensification of ^1^H and ^19^F NMR peaks; often observed during work-up (^2^*J*_H–F_ ≈ 53 Hz, ^1^H (*δ*) 6.08 ppm and ^19^F (*δ*) −146.7 ppm; for an anisotropic mixture of 90/10 C_6_F_6_/C_6_D_6_).

**Fig. 3:**
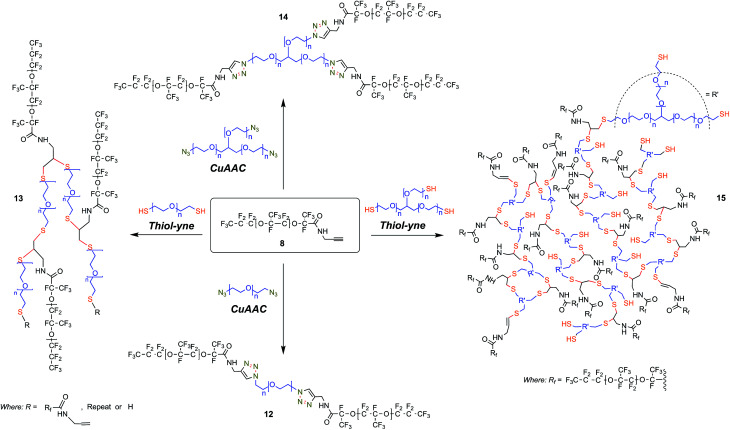
Surfactant synthesis and resulting structure diversity highlighting the central role played by the intermediate Krytox propargyl amine (8) that can be combined with either a homo-bifunctional PEG or a 3-arm homo-functional glycerol ethoxylate by CuAAC or thiol–yne reactions. Those reactions permit the generation of PFPE–PEG surfactants with a variety of structures. CuAAC (12): 10 mol% Cu(OAc)_2_, 20 mol% sodium ascorbate, 16 mol% neocuproine; 1 : 1 HFE 7100 : 1 : 1 MeOH/H_2_O, 60 °C, 48 h, thiol–yne (13): 0.2 equiv. DMPA, UV 365 nm; 2 : 1 HFE 7100 : MeOH, RT, 48 h, CuAAC (14): 15 mol% Cu(OAc)_2_, 35 mol% sodium ascorbate, 30 mol% TBTA; 1 : 1 HFE 7500 : 2 : 1 MeCN/H_2_O, 80 °C, 48 h, thiol–yne (15): 0.2 equiv. DMPA, UV 365 nm; 2 : 1 HFE 7100 : MeOH, RT, 48 h.

In contrast, we prepared a stable click intermediate PFPE propargyl derivative (8) that can be used as a basis to elaborate a library of materials with linear, brush-like and hyperbranched polymer architectures (Fig. 1c and 2b). In total, we generated four structurally distinct surfactants by coupling the PFPE propargyl derivative 8 to either a homo-bifunctional PEG or a 3-arm homo-functional glycerol ethoxylate by CuAAC or thiol–yne reaction (Fig. 3). Starting with intermediate 8, these materials are accessed in a single step without the need for specialized training, techniques or equipment. Another decisive advantage of this approach is the commercial availability of click ready moieties that present numerous opportunities for the construction of designer interfaces.

We attempted to maintain a constant hydrophilic–fluorophilic balance to directly compare the synthesized materials and evaluate their structure–property relationship. Previous molecular weight studies have shown that the combination of 600 g mol^−1^ PEG with 6000 g mol^−1^ PFPE performed best in droplet formation and long-term droplet stability.^18^ In choosing reaction conditions for this work, we strove to fulfill the basic criteria commonly ascribed to ‘click’ reactions. We elected to utilize solvent systems that would allow for facile product isolation to provide operational simplicity. As presented, products are isolated directly from the crude reaction mixture by simple phase separation. Propargyl derivative 8 was heated to 80–90 °C in a vacuum oven for 72 h to convert any 10 present to non-surface active 11. The outcome of the different reactions is reported in Table 1.

**Table 1:** Outcome of the CuAAC and photo-activated thiol–yne reactions. Note: calculations exclude glycerol backbone, and only account for PEG MW. See ESI for total compositional analysis

Structure	Entry	PEG MW/PFPE Chain	PFPE *M*_n_ (^19^F NMR)	Conversion(^1^H NMR)	Physical aspect at 30% wt in HFE 7500
Linear PFPE–PEG triazole linked triblock	(12)	∼300 g mol^−1^	5840	95%	Cloudy and yellowish with streaks
Brush-like sulfide linked PFPE–PEG	(13)	∼300 g mol^−1^	5840	71%	Clear and yellowish
3-arm star PFPE–PEG triazole linked tetrablock	(14)	∼300 g mol^−1^	5840	70%	Clear and very pale yellow
Hyperbranched sulfide linked PFPE–PEG	(15)	∼600 g mol^−1^	5840	65%	Cloudy and yellowish with streaks

### CuAAC reaction

CuAAC reaction conversion was determined by comparing integrated values of propargyl derivative 8 CH_2_–C≡CH and O–CH_2_–C≡ ^1^H NMR resonances to those of the resultant triazole function and neighboring α protons. Using this molecular characterization protocol, we conclude that *ca.* 95% conversion to the desired structure was obtained in the case of linear triblock copolymer 12, whereas *ca.* 70% was achieved for 3-arm star tetrablock copolymer 14. FT-IR spectra of 12 and 14 also exhibit salient features which permit monitoring of reaction progress, notably bands present at 2375 and 3307 cm^−1^ likely corresponding to the –C≡C– stretch and –C≡C–H stretch respectively.

We tested diverse fluorinated solvents that exhibit a range of coordination strength and miscibility with organic solvent: (1) a perfluorotrialkylamine (perfluorotripropylamine, FC 3283), which displays immeasurably small interactions with ions, negligible basicity^53^ and is immiscible with organic solvents, did not permit solvent tuning and does not possess favorable volatility; (2) fluorinated alcohols hexafluoroisopropanol (HFIP) and trifluoroethyl alcohol (TFE), which possess high ionizing power, unique hydrogen bonding capability, miscibility with organic solvents and are known to provide rate acceleration of certain reactions,^54,55^ were unsuccessful when utilized as co-solvents; (3) among perfluorinated ethers, which exhibit experimentally significant ion interactions,^53^ the hydrofluoroethers methoxyperfluorobutane (HFE 7100) and 2-(trifluoromethyl)-3-ethoxydodecafluorohexane (HFE 7500) proved successful and were generally preferred due to their miscibility with certain organic solvents. Solvent systems containing perfluoromethoxybutane are limited by its relatively low boiling point of 60 °C and for glycerol ethoxylate triazide, a more challenging substrate, we used the higher boiling HFE 7500 (130 °C); however, HFE 7500 is not as easily removed from viscous materials such as the 3-arm star PFPE–PEG 14.

In addition to increasing the availability and catalytic activity of copper species, ligands serve to improve the stability of the Cu(i) oxidation state. Neocuproine, which has proven effective for support of CuAAC when used in the appropriate ligand : Cu ratio,^43^ was found sufficient in the case of linear triblock copolymer 12. In contrast, three-arm star tetrablock copolymer 14 required the use of TBTA, a commercially-available ligand that can also be easily produced following published procedures.^43^

One of the challenges of the proposed synthesis is that reactions must occur in fluorinated media. Indeed, the propargyl derivative 8 is not appreciably surface active (unable to form even transiently stable hand shake emulsions), and is likely to preferentially reside in the fluorous phase. Consequently, in the case of CuAAC we assume the generation of copper(i) acetylides either needs to occur in a predominately fluorous phase or is a kinetically constrained interfacial process. Notably, molecules generally need 60% or better fluorine content to have acceptable partition coefficients for the fluorous phase;^56^ this limits the concentration of the Cu(i) catalyst in this phase. However, fluorinated solvents which display miscibility with conventional organic solvents may serve to circumvent this requirement to some degree. Nonetheless, we cannot eliminate the possibility that the availability of the transition metal catalyst in the fluorous phase limits the reaction efficiency. This assumption is supported by the observation that supporting ligands were necessary to achieve quantitative end group conversion in the case of 12. This is compounded by the fact that copper(i) acetylides in the CuAAC tend to oligomerize^57^ and that the amphiphilicity of the PFPE acetylide itself could potentially result in the formation of metastable aggregates. Accordingly, careful consideration was given to the choice of solvent, whose role was to optimize conversion efficiency while being readily removable to fulfill one of the click criteria.

Another possible factor limiting the success of CuAAC is the increased solubility of oxygen in fluorinated solvents which could promote oxidation of the active catalyst.^58^

### Thiol–yne reactions

Hyperbranched polymers display distinct behaviors from their linear analogues, and we sought simple ways to synthesize such molecules starting with the propargyl derivative 8 to probe structure–property relationships. As reported by Perrier *et al.* in 2009 (ref. 38) thiol–yne chemistry allows for the creation of hyperbranched polymers under UV irradiation in the presence of the photo-initiator DMPA at room temperature. In addition, thiol–yne chemistry can be used to produce brush-like or comb-like polymers, materials that provide an intermediate point relative to their linear block copolymer type analogues.^59,60^

Similar to the CuAAC reactions, the fluorous environment created unique challenges for the thiol–yne reactions. Though the presence of oxygen is generally well tolerated for thiol–yne reactions,^37^ an increased disulfide formation could result from the unique solubility of oxygen in fluorocarbon solvents.^61^ Further, undissolved DMPA was often observed in both trial and scale reactions, which reveals limited solubility of the photo-initiator. Despite these potential limitations, these reactions were successful and did not require further optimization.

In the case of structures 13 and 15 potential exists for attachment of up to two PEG polymer chains per alkyne moiety with a vinyl sulfide resulting from a single addition and a 1,2-disubstituted adduct from two. Peaks attributable to the presence of vinyl sulfide can be clearly observed in the ^1^H NMR spectra of 13 and 15 at ∼6.3 and 5.7 ppm which is consistent with prior reports. Cook *et al.*^62^ report a method to ascertain degrees of branching; they cite an equation first introduced by Hawker and Fréchet,^63^ degrees of branching (DB) = (*D* + *T*)/(*D* + *T* + *L*), noting that in the case of alkyne/thiol monomer addition degree of branching can be fairly easily determined. In this case, integration of the region of interest in the ^1^H NMR spectrum is convoluted by a doublet at 5.78 ppm originating due to the presence of 11. In our calculations, we slightly modified this method to account for the presence of other interfering signals originating from the PEG polymer repeat units which overlap with the C(O)–NH–CH_2_ resonance. We utilized the averaged integral values of –CH_2_–C≡CH and C(O)–NH–CH_2_ signals occurring at 2.1 ppm and 6.76 ppm respectively to determine parameter *T*. Substituting for *D*, *T* and *L* we obtained values of 0.92 and 0.74 DB for materials 13 and 15 respectively (see ESI†). Similarly, we derived values of 71% and 65% conversion for our brush-like 13 and hyperbranched 15 architectures, respectively. The low overall degree of branching observed for 15 can likely be reconciled based upon structural arguments such as steric crowding.

Our click approach to the synthesis of fluorinated surfactants yielded four different structures that are based on the same tail and heads with very similar properties in different configurations. Those surfactants offer a unique opportunity to analyze their structure–function relationship. To date, only the effect on the interfacial energy of different hydrophilic heads on a fluorinated tail has been reported.^17^

### Physico-chemical characterization

**Fig. 4:**
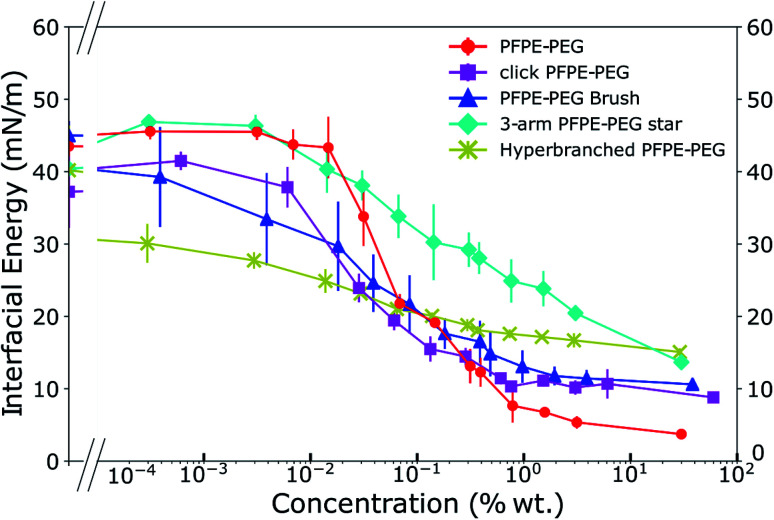
Interfacial energies of the click fluorinated surfactants in HFE 7500 as a function of their weight concentrations reveal a wide range of behaviors.

We characterized the interfacial energy of de-ionized water with HFE 7500 containing the different click surfactants and used the PFPE–PEG surfactant as a reference point. As previously noted,^11,29^ the use of HFE 7500 yields more stable droplets compared to other fluorinated oils such as FC 40 or FC 3283. We implemented a pendent drop method to obtain the interfacial energies (Fig. 4), adsorption kinetics (ESI Fig. 1†), and the critical micelle concentration (cmc) of each surfactant (ESI Fig. 2†). Those characteristics are presented in Table 2.

**Table 2:** Physico-chemical characterization of surfactants dissolved in HFE 7500 obtained by the pendent drop method with de-ionized water. HFB: Hydrophilic–Fluorophilic Balance

Surfactant in HFE 7500	HFB	*τ*_equilibrium_ (min)	*γ*_CMC_ (% wt)	*γ*_CMC_ (mN m^−1^)
PFPE–PEG	0.98	0.2	0.6	5.1
Click PFPE–PEG	0.98	0.9	0.3	8.6
3-arm PFPE–PEG star	1.08	0.2	>30	<16.4
Brush-like PFPE–PEG	1.02	3.4	1.3	10.6
Hyperbranched PFPE–PEG	2.05	5.6	1.5	16.4

The kinetics analysis reveals that the PFPE–PEG 9 and the 3-arm star PFPE–PEG 14 adsorb onto the interface at a similar rate, while the click PFPE–PEG 12 is slightly delayed and the bulkier brush-like 13 and hyperbranched PFPE–PEG 15 reach the interface at a longer time-scale. The cmc are in the same range for the PFPE–PEG 9 and click PFPE–PEG 12, slightly higher for the brush-like 13 and hyperbranched 15 surfactants while the interfacial energy in presence of increasing concentrations of the 3-arm star surfactant 14 does not reach a plateau at concentration as high as 30% in weight. The asymptotic interfacial energies in presence of PFPE–PEG 9 and click PFPE–PEG 12 are comparable and lower than the brush-like surfactant 13 while the 3-arm star 14 and the hyperbranched 15 structures generate similar but much higher values. In all cases, the decrease in interfacial energy is at least 20 mN m^−1^ compared to the pure oil, which qualifies those surfactants as good surfactant for droplet microfluidics following previously established criteria.^17^ The value of the interfacial energy of the PFPE–PEG surfactant 9 is slightly higher than we previously reported^64^ but in the range reported by others;^8^ however, the value of its critical micellar concentration is one order of magnitude higher than previously reported by the same group. During synthesis, we have degraded any impurities into non surface-active impurities through decarboxylation. Hence our measurement of interfacial energy should not be affected by those species.

We can observe a trend in the curves that describe the interfacial energies as a function of concentration (Fig. 4) where the click PFPE–PEG 12 surfactant behaves similarly to the PFPE–PEG 9, but the curves tend to flatten with the surfactant complexity. The 3-arm star PFPE–PEG 14 exhibits behavior that departs from this observation because it does not reach any saturation point below 30% wt concentration. This may be explained by the rigid structure of this surfactant, which possesses three arms that prevent the amphiphilic moieties to easily segregate across the interface.

The hyperbranched structure can be considered as the functional equivalent of a micelle; the important distinction is that it is unimolecular and therefore kinetically stable. Based on molecular structure, we can attempt to explain the observation that the ultimate surface tension of 15 is higher than its linear 9, 12, 3-arm star 14 and brush-like 13 counterparts, where its bulky, highly branched globular structure likely yields less ordered and efficient packing, leading to lower overall density at the interface. It is likely that all the PEG moieties of the branched surfactant do not point toward the aqueous phase when the molecules are at the interface but that most of them are caged within an intramolecular micelle. That stability is also reflected in the equilibration time because the more branched surfactants exhibit longer kinetics to reach interfacial equilibrium.

### Performance of surfactants for droplet microfluidics

We tested the ability of the surfactants to support droplet microfluidic applications by performing stability, molecular and cellular compatibility experiments.

We first generated emulsions of Phosphate Buffer Saline (PBS) in HFE 7500 fluorinated oil containing 3% wt, which is above the cmc, of the different surfactants. Using a droplet generator with pressure control, we observed that all the surfactants enabled stable droplet generation with volume fractions and droplet volumes similar to those of the PFPE–PEG surfactant 9 (ESI Fig. 3†). In addition, after collecting and storing the emulsions at room temperature we quantitatively observed that the surfactants yielded stable emulsions at 30 days post-generation with the click PFPE–PEG 12 performing as well as the PFPE–PEG 9 surfactant with over 97% stability while a stability of 92% was recorded for the other surfactants (ESI Fig. 4†).

### Compatibility with molecular reactions

We tested the compatibility of the surfactants with an isothermal reaction and a polymer chain reaction (PCR) by testing their capacity in supporting the amplification of a portion of the oncogene KRAS cloned into a plasmid. We successively generated emulsions of 280 pL droplets containing the reactions within HFE 7500 oil containing 3% wt of the surfactants, and reported emulsion stability and amplification outcome. Emulsion stability assessment was either qualitative following a scoring method based on visual observation (ESI Fig. 5†), or quantitative after measuring the percentage of intact droplets.

For the isothermal molecular amplification, all but the 3-arm star PFPE–PEG surfactant 14 yielded qualitatively stable emulsions during the isothermal reaction at 65 °C for 90 min. The isothermal reaction yielded DNA amplification only for the PFPE–PEG surfactant 9 (*n* = 2). Surprisingly, even the 3-arm star PFPE–PEG surfactant 14 despite its greatly reduced surface to volume ratio due to the major collapse of the emulsion did not support DNA amplification. Vortex generated emulsions indicated that only the use PFPE–PEG surfactant 9 resulted into a robust amplification (*n* = 2, ESI Fig. 6†). A phase of HFE 7500 oil only, which has the lowest surface over volume ratio, also resulted in poor amplification. We also checked that the surfactants did not promote molecular transport into the fluorinated phase^11,20,50^ by testing the partition of DNA staining dye, Sytox Orange, in handshake emulsions and using the ionic surfactant as a positive control (ESI Fig. 7†). This result suggests that sequestration of ions such as Mg^2+^ into micelles is unlikely with those surfactants. To reconcile those observations, we hypothesized that the presence of an accessible interface partially inhibited the isothermal amplification as previously observed in other settings.^65^ The hypothesis was further supported by the fact that the addition of Tetronic 1307 resulted in very robust amplification with all the different surfactants. Interestingly, the addition of Tetronic 1307 in the aqueous phase did not improve emulsion stability that remained quantitatively stable for the PFPE–PEG and the click PFPE–PEG surfactants at 94% and 92% respectively (ESI Fig. 8†).

PCR thermocycling is known to be a challenging application for emulsion stability. In our experiments, the addition of Tetronic 1307 was required to obtain even qualitatively stable emulsions (ESI Table 1†). In terms of stability, the PFPE–PEG surfactant 9 surpassed the brush-like PFPE–PEG 13 and the click PFPE–PEG surfactants 12. The 3-arm star PFPE–PEG 14 and hyperbranched PFPE–PEG 15 yielded emulsions marginally stable during thermocycling. We tested the ability of the 3-arm star PFPE–PEG 14 and the brush-like PFPE–PEG 13 surfactants to replace the Tetronic 1307 as a stabilizing co-surfactant for PCR applications. The addition of the 3-arm star PFPE–PEG 14 to the click PFPE–PEG 12 surfactant occasionally yielded improved stability. The other combinations did not show any improvement in emulsion stability. Overall, the click PFPE–PEG 12 did not exhibit satisfactory stability for PCR applications, its best performance consisted in a 75% stable emulsion with the use of Tetronic 1307. The click PFPE–PEG 12 is not appreciably different structurally from the PFPE–PEG 9 but did not exhibit the same level of performance. Emulsion made with PFPE–PEG 9 were consistently over 90% stable in the presence of Tetronic 1307 (ESI Fig. 9†). The click surfactants, except for the brush-like version 13, were synthesized with polydisperse PEG molecules that are thought to be beneficial to colloidal stabilization. We hypothesize that the phase separation step of the work-up procedure resulted in preferential extraction of MW species possessing more favorable higher HFB values; these species have proven to be advantageous for stabilization during thermal-cycling (unpublished data). This is known to be problematic in the case of the PFPE–PEG surfactant 9 and formalized synthetic procedures often avoid the use of filtration media such as Celite and/or exclude steps such as liquid–liquid extraction to prevent the selective removal of specific MW fractions. Liquid–liquid extraction was warranted in the case of the click PFPE–PEG surfactant to remove the catalyst system because Cu traces could generate toxic reactive oxygen species.^66^ This hypothesis could be further tested by generating a similar click PFPE–PEG surfactant with higher MW PEG molecules.

### Surfactant biocompatibility with mammalian cells

**Fig. 5:**
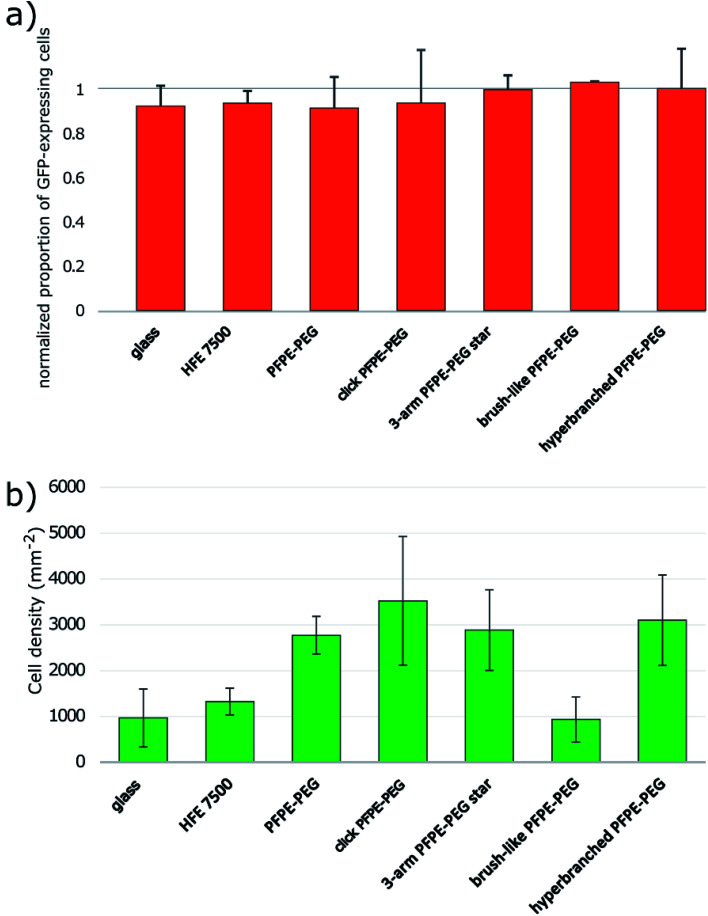
(a) All surfactants exhibit biocompatibility with CHO cells in overlay assays after 16 hours of incubation. The proportion of GFP expressing cells is normalized by the value at time 0. (b) Cell aggregation is promoted by all the surfactants except the brush-like PFPE–PEG after 1 hour of incubation.

We tested the effect of the surfactant on mammalian cells by performing overlay assays^11,12^ modified to minimize the amount of surfactant used per experiment (ESI Fig. 10†). We observed Green Fluorescent Protein (GFP) expressing Chinese Hamster Ovary (CHO) cells seeded onto different oil formulations over the course of several hours and used the GFP signal as a proxy for cell viability. We normalized the rate of GFP expressing cells after 16 hours of incubation with the initial rate at time 0. Those data highlight that all surfactants are biocompatible at this timescale (Fig. 5a). In addition, we observed that the ability of cells to cluster at the center of the microwells varied as a function of the surfactant. We checked that the curvatures of the interfaces generated with the different formulations were similar both in orientation and amplitude and could not simply explain the observation. All surfactants except the brush-like PFPE–PEG (13) surfactant promote cell aggregation (Fig. 5b, ESI Fig. 11†). Cells seeded onto the brush-like PFPE–PEG (13) remained dispersed similarly to cells onto HFE 7500 oil only or on the glass slide. This effect may be due to a low density of PEG moieties effectively at the interface, which then behaves as a “naked” interface from the cells' point of view. Alternatively, those surfactants molecules may have a limited motility within the interface and act as anchors in a similar fashion to Pickering nanoparticles.^26^ The addition ot Tetronic 1307 that absorbs onto the interface enabled cell aggregation in overlays with the brush-like PFPE–PEG (13) surfactant. Those observations have practical implications because we have shown that microfluidic droplets can be used to generate highly controlled cell aggregates for high-throughput screening.^67^ Remarkably, a simple overlay experiment can reveal some properties of surfactants at molecular scale at the interface.

Our results indicate that the click-PFPE–PEG (12) performs better than the 3-arm star PFPE–PEG (14), the brush-like PFPE–PEG (13) and the hyperbranched PFPE–PEG (15) to stabilize microfluidic droplets. Droplet stability hinges on the repulsive effects of interfaces, which are mediated by the tails of surfactant molecules. The emulsion stability thus depends on the coverage of the interface by the surfactant. Isothermal amplification experiments showed a lower coverage of the interface by the 3-arm star PFPE–PEG (14), the brush-like PFPE–PEG (13) and the hyperbranched PFPE–PEG (15). The results with cells further indicate that the density of PEG molecules at the interface is low for the brush-like PFPE–PEG (13). Altogether, these results suggest a deficit of interface coverage by those surfactants.

Our hypothesis is that the 3-arm star PFPE–PEG (14) has a 3-dimensional structure that makes it difficult to have the three PFPE chains point in the same direction, which would limit its ability to cover an interface as demonstrated by the absence of a clear cmc even at concentration up to 30% wt in HFE 7500. The brush-like PFPE–PEG (13) and the hyperbranched PFPE–PEG (15) are bulkier than the click-PFPE–PEG (12) and their kinetics of adsorption and interfacial energy above cmc are less favorable to droplet stability. The brush-like PFPE–PEG (13) cannot adsorb efficiently at the interface, possibly because the PEG molecules create an intramolecular micelle, while the hyperbranched PFPE–PEG (15) is likely to behave as a molecular micelle with a limited fraction of PEG moieties available to lower the interfacial energy.

## Conclusion

In this work, CuAAC and thiol–yne click reactions provided access to polymer architectures previously untested in droplet microfluidics. PEG and related analogues served as recurrent structural motifs for the synthesis of a diverse library of materials. In the case of CuAAC, unexpectedly low overall reaction efficiencies were observed in trial reactions where ligands were not employed. Notably, the use of supporting ligands was shown to afford higher levels of conversion. The observed inefficiency may arise from limited availability of catalytically active species; potentially attributable to fluorous phase solubility and/or oxidative instability of Cu(i) given the solubility of oxygen in fluorinated media. Thiol–yne reactions were found to be amendable to the fluorous phase as well, with the added benefit that they required relatively little refinement of reaction conditions. Nonetheless, continued testing and development of catalyst systems will likely be necessary to ensure the presented method is sufficiently general; it is presumed that those more directly applicable to fluorinated environments will prove to be superior. Furthermore, it is suspected that some benefit might be derived from suitable fluorinated photoinitiators as well; reports of such materials already exist for 2-hydroxy-2-methylpropiophenone.^68^ Alternatively, development of a surface active click capable intermediate could serve to obviate some of these considerations. This contrasts with previous report of click coupling of PFPE–PEG molecules for energy storage applications.^41^ Our synthetic route to sulfide linked PFPE–PEG materials also differed from previous work^40^ because we used photoinduction and not thermal induction to initiate the reaction. This difference could prove beneficial for the attachment of sensitive biomolecules.

The surfactants exhibited physico-chemical properties in agreement with their branching complexity with extended equilibrium time and higher cmc with increasing complexity. Only the 3-arm star PFPE–PEG 14 did not conform to this behavior, certainly due to its structure that prevents efficient adsorption to the interface. The utility of the click synthesized surfactants for droplet microfluidics is supported by the long-term stability of PBS emulsions. The click PFPE–PEG 12 surfactant also performed very well during isothermal amplification reaction in conjunction with Tetronic 1307 yielding robust amplifications and stable emulsions. The PCR application revealed too challenging for the surfactants we tested but supported the hypothesis that the extraction step during synthesis work up removes molecules with high HFB values that are partially responsible for emulsion stability during thermocycling. Finally, cell interaction experiments revealed strikingly different behaviors depending on the surfactant structures. The brush-like structure 13 did not support cell aggregation like the other surfactants. The differential aggregation behavior of the different surfactants was an unexpected observation from the real-time overlay experiment and may inform on the way the surfactant molecules absorb onto the interface at the molecular scale.

In this report, we successfully demonstrated the synthesis of novel fluorinated PFPE–PEG based surfactants using click chemistries that enabled the simple derivation of structural variants that were tested for their use in droplet microfluidics. This strategy greatly simplifies the development of designer interfaces for droplet microfluidics and will empower investigators to explore novel surfactants.

## Conflicts of interest

There are no conflicts to declare.

## Supplementary Material

RA-008-C8RA01254G-s001
