# Correction: Click chemistry approaches to expand the repertoire of PEG-based fluorinated surfactants for droplet microfluidics

**DOI:** 10.1039/c8ra90097c

**Published:** 2019-01-02

**Authors:** Randall Scanga, Lucie Chrastecka, Ridhwan Mohammad, Austin Meadows, Phenix-Lan Quan, Eric Brouzes

**Affiliations:** Department of Chemistry, Massachusetts Institute of Technology Cambridge Massachusetts USA randallscanga@yahoo.com; Department of Biomedical Engineering, Stony Brook University Stony Brook New York USA eric.brouzes@stonybrook.edu; Laufer Center for Physical and Quantitative Biology, Stony Brook University Stony Brook New York USA

## Abstract

Correction for ‘Click chemistry approaches to expand the repertoire of PEG-based fluorinated surfactants for droplet microfluidics’ by Randall Scanga *et al.*, *RSC Adv.*, 2018, **8**, 12960–12974.

The authors regret that during production of the published version of their article the bold formatting in the NMR data to indicate the nuclei of interest was lost. The correctly formatted Synthesis section of the Materials and methods is presented below.

## Synthesis

### PFPE acid chloride (7)

The procedure for preparation of the PFPE acid chloride adheres to the previously published method with a few minor alterations^18,44^ (see ESI). Starting with the commercially available end functional PFPE carboxylic acid Krytox FS(H) 157 (DuPont), the corresponding acid chloride is obtained by treatment with oxalyl chloride in refluxing methoxyperfluorobutane (HFE 7100, 3M). The newly generated acid chloride is isolated *in situ* under reduced pressure and redissolved in FC 3283. The resultant solution/suspension is then filtered under inert atmosphere and used without further purification in the subsequent amidation reaction.

^19^F NMR (C_6_D_6_/C_6_F_6_, 282 MHz) *δ* ppm: −80.67 (m, 5F, O–CF(C**F**_**3**_)–C**F**_**2**_), −81.96 (m, 3F, C**F**_**3**_–CF_2_–CF_2_), −82.57 (m, 3F, C**F**_**3**_–(CF)–C=O), −125.68 (m, 1F, CF_3_–(C**F**)–C=O), −130.48 (s, 2F, CF_3_–C**F**_**2**_–CF_2_), −144.87 (m, 1F, O–C**F**(CF_3_)–CF_2_).

### PFPE amides (8 and 9)

The amidation reaction is performed in a biphasic solvent system comprised of FC 3283 and THF in the presence of TEA which serves as an HCl scavenger (see ESI and Holtze *et al.*^18^). The desired amine coupling partner dissolved in THF is added dropwise over *ca.* 30 min to the fluorocarbon phase resulting in the formation of a fine emulsion. After *ca.* 24 h the crude reaction mixture is isolated *via* rotary evaporation and once again redissolved in FC 3283 and filtered to obtain a clear, viscous, yellow oil.

Propargyl derivative (8) ^1^H NMR (C_6_D_6_/C_6_F_6_, 300 MHz) *δ* ppm: 6.58 (br, 1H, C(O)–N**H**–CH_2_), 4.12 (m, 2H, O–C**H**_**2**_–C≡), 2.18 (t, 1H, –C≡**H**).

^19^F NMR (C_6_D_6_/C_6_F_6_, 282 MHz) *δ* ppm: −80.74 (m, 5F, O–CF(C**F**_**3**_)–C**F**_**2**_), −82.54 (m, 3F, C**F**_**3**_–CF_2_–CF_2_), −83.72 (m, 3F, C**F**_**3**_–(CF)–C=O), −130.47 (s, 2F, CF_3_–C**F**_**2**_–CF_2_), −133.39 (m, 1F, CF_3_–(C**F**)–C=O), −144.86 (m, 1F, O–C**F**(CF_3_)–CF_2_).

## CuAAC reactions

### PFPE–PEG triazole linked triblock copolymer (12)

3.0 mL of methoxyperfluorobutane (HFE 7100) was added to 3.0 g (0.51 mmol) of PFPE-propargyl derivative and sonicated to obtain a clear, homogeneous solution. A solution of 164 mg (0.27 mmol) of PEG 600 diazide in 1.5 mL of MeOH was prepared and sonicated. Next, 0.0102 g (0.051 mmol, 10 mol%) Cu(OAc)_2_ and 17 mg of neocuproine (0.0815 mmol, 16 mol%) were added to the previously generated PEG 600 diazide/MeOH solution and further sonicated.

20.2 mg (0.102 mmol) of sodium ascorbate was then combined with 1.5 mL of DI H_2_O, mixed and transferred to the PEG 600 diazide/Cu(OAc)_2_/neocuproine/MeOH solution. Finally, the combined solution was added to the PFPE-propargyl derivative/HFE 7100 solution in a single portion, and stirred at 1200 rpm with a 1′′ × 1/2′′ PTFE coated stir bar at room temperature (r.t.) for *ca.* 1 h before heating to 60 °C. After *ca.* 48 h, 6.0 mL of MeOH was added to the crude reaction mixture and stirred vigorously for *ca.* 5 min. The resulting destabilized emulsion was left to stand until complete phase separation was noted. The fluorocarbon phase was then extracted and dried over MgSO_4_ under stirring for *ca.* 30 min before filtering through a 0.2 μm PFTE syringe filter. Finally, solvent was removed under reduced pressure.

^1^H NMR (C_6_D_6_, 300 MHz) *δ* ppm: 8.49 (br, 1H, C(O)–N**H**–CH_2_), 7.9 (s, 1H, –CH_2_–CN=C**H**–), 4.67 (br, 2H, –NH–C**H**_**2**_–CN=), 4.55 (br, 2H, –N–C**H**_**2**_–CH_2_–), 3.9 (br, 2H, –N–CH_2_–C**H**_**2**_–), 3.6 (br, 2H, –O–C**H**_**2**_–CH_2_–). FTIR *ν* cm^−1^: 2874 (–C–H), 1721 (C(NH)=O), 1533 (amide II), 1306–1130 (C–F, C–O, overlapping).

### PFPE–PEG triazole linked tetrablock copolymer (14)

A solution of 1.5 mL 2-(trifluoromethyl)-3-ethoxydodecafluorohexane (HFE 7500) and 1.5 g (0.254 mmol, 3.0 eq.) of PFPE-propargyl derivative was prepared. 95.6 mg (0.0889 mmol, 1.05 eq.) of glycerol ethoxylate triazide and 0.975 mL of MeCN was prepared and sonicated. Next, 0.00761 g (0.0381 mmol, 15 mol%) Cu(OAc)_2_ and 40.8 mg of tris-(benzyltriazolylmethyl)amine (TBTA) (0.0815 mmol, 16 mol%) were added to the previously generated glycerol ethoxylate triazide/MeCN solution and sonicated. 17.6 mg (0.0889 mmol) of sodium ascorbate was then combined with 0.525 mL of DI H_2_O, mixed and transferred to the glycerol ethoxylate triazide/Cu(OAc)_2_/TBTA/MeCN solution. Finally, the combined solution was added to the PFPE-propargyl derivative/HFE 7500 solution in a single portion, and stirred at 1200 rpm with a 1′′ × 1/2′′ PTFE coated stir bar at r.t. for *ca.* 1 h before heating to 80 °C. After *ca.* 48 h, 6.0 mL of MeOH was added to the crude reaction mixture and further stirred for *ca.* 5 min. Stirring was discontinued and the destabilized emulsion was left to phase separate. The fluorocarbon phase was then isolated and dried over MgSO_4_ under stirring for *ca.* 30 min before filtering through a 0.2 μm PFTE syringe filter. Finally, solvent was removed under reduced pressure at elevated temperature (70–80 °C).

^1^H NMR (C_6_D_6_, 300 MHz) *δ* ppm: 9.15 (br, 1H, C(O)–N**H**–CH_2_), 7.97 (br, 1H, –CH_2_–CN=C**H**–), 4.57 (br, 2H, –N–C**H**_**2**_–CH_2_=), 3.54 (br, 2H, –O–C**H**_**2**_–CH_2_–).

## Thiol–yne reactions

### Brush-like PFPE–PEG polymer (13)

2.0 g (0.34 mmol, 1 equiv.) of PFPE-propargyl derivative was dissolved in 7 mL of methoxyperfluorobutane. Next, 106.8 mg (96 μL, 0.34 mmol, 1.0 equiv.) of hexa(ethylene glycol)dithiol and 17.6 mg (0.068 mmol, 0.2 equiv.) of 2,2-dimethoxy-2-phenylacetophenone DMPA were combined and immediately dissolved in 3.5 mL of MeOH. The DMPA/thiol/MeOH solution was then transferred to the previously generated PFPE-propargyl derivative/HFE 7100 solution and the vial sealed with a septum. The sealed vessel was then flushed under positive pressure of N_2_ and irradiated at 365 nm while stirring at *ca.* 1200 rpm, at r.t. overnight. The following day the crude reaction mixture was combined with an equivalent volume of MeOH (10.5 mL) and allowed to stand until complete phase separation was noted. The fluorous phase was then isolated and directly evaporated *in vacuo* to obtain a clear, colorless oil.

^1^H NMR (C_6_D_6_, 300 MHz) *δ* ppm: 8.35 (br, 1H, C(O)–N**H**–CH_2_), 7.42 (br, 1H, –N**H**–CH_2_–CH=), 6.24 (br, 1H, –C**H**=CH–S), 5.68 (br, 1H, –CH=C**H**–S), 3.67 (br, 2H, –O–C**H**_**2**_–CH_2_–), 3.02 (1H, –C**H**–CH_2_–S), 2.84 (1H, –CH–C**H**_**2**_–S).

### Hyperbranched PFPE–PEG (15)

2.0 g (0.339 mmol, 2 equiv.) of PFPE-propargyl derivative was dissolved in 7 mL of methoxyperfluorobutane. Next, 314 mg (0.285 mmol, 3.78 equiv.*) of glycerol ethoxylate trithiol and 17.4 mg (0.0678 mmol, 0.2 equiv.) of 2,2-dimethoxy-2-phenylacetophenone DMPA were combined and immediately dissolved in 3.5 mL of MeOH. The DMPA/thiol/MeOH solution was then transferred to the previously generated PFPE-propargyl derivative/HFE 7100 solution and the vial sealed with a septum. The sealed vessel was then thoroughly flushed with N_2_ and irradiated at 365 nm under vigorous stirring, at r.t. overnight. After *ca.* 24 h the crude reaction mixture was combined with an equivalent volume of MeOH (10.5 mL) and the phases were allowed to separate. Finally, the fluorous phase was extracted and evaporated *in vacuo* to obtain a clear, colorless oil. *Stoichiometry adjusted to account for the presence of disulfide.

^1^H NMR (C_6_D_6_, 300 MHz) *δ* ppm: 8.68 (br, 1H, C(O)–N**H**–CH_2_), 7.84 (br, 1H, –N**H**–CH_2_–CH=), 6.38 (br, 1H, –C**H**=CH–S), 5.68 (br, 1H, –CH=C**H**–S), 3.75 (2H, –O–C**H**_**2**_–CH_2_–), 3.09 (1H, –C**H**–CH_2_–S), 2.9 (2H, –CH–C**H**_**2**_–S).

The Royal Society of Chemistry apologises for these errors and any consequent inconvenience to authors and readers.

## Supplementary Material

