# Supplementary material for: Click chemistry approaches to expand the repertoire of PEG-based fluorinated surfactants for droplet microfluidics
Source: RSC Adv. 2018 Apr 6;8(23):12960–74. doi: 10.1039/c8ra01254g (PMC6779154; doi:10.1039/c8ra01254g)

Electronic Supplementary Material (ESI) for RSC Advances.
This journal is © The Royal Society of Chemistry 2018

Click Chemistry Approaches to Expand the Repertoire of PEG-based Fluorinated Surfactants for Droplet Microfluidics

Supplemental information
Table of content

Supplemental Figure 1	Pendent drop kinetics	p. S2
Supplemental Figure 2	Determination of surfactant cmc	p. S3
Supplemental Figure 3	D-PBS droplet generation	p. S4
Supplemental Figure 4	D-PBS emulsion stability at 30 days	p. S5
Supplemental Figure 5	Scoring system for qualitative assessment of emulsion stability	p. S5
Supplemental Figure 6	Electrophoresis of HDA products	p. S6
Supplemental Figure 7	Molecular transport test	p. S6
Supplemental Figure 8	Emulsion stability after isothermal HDA	p. S7
Supplemental Figure 9	Emulsion stability after PCR	p. S7
Supplemental Figure 10	Schematic of the device for low volume overlay experiments	p. S8
Supplemental Figure 11	Clustering behavior of CHO cells on various materials	p. S8
Supplemental table	Qualitative assessment of emulsion stability after PCR	p. S9
Supplemental Information 1	Experimental conditions and NMR characterization of the click-based surfactant synthesis	p. S10-S35
Supplemental Information 2	NMR characterization of the PFPE-PEG surfactant synthesis	p. S36-S42

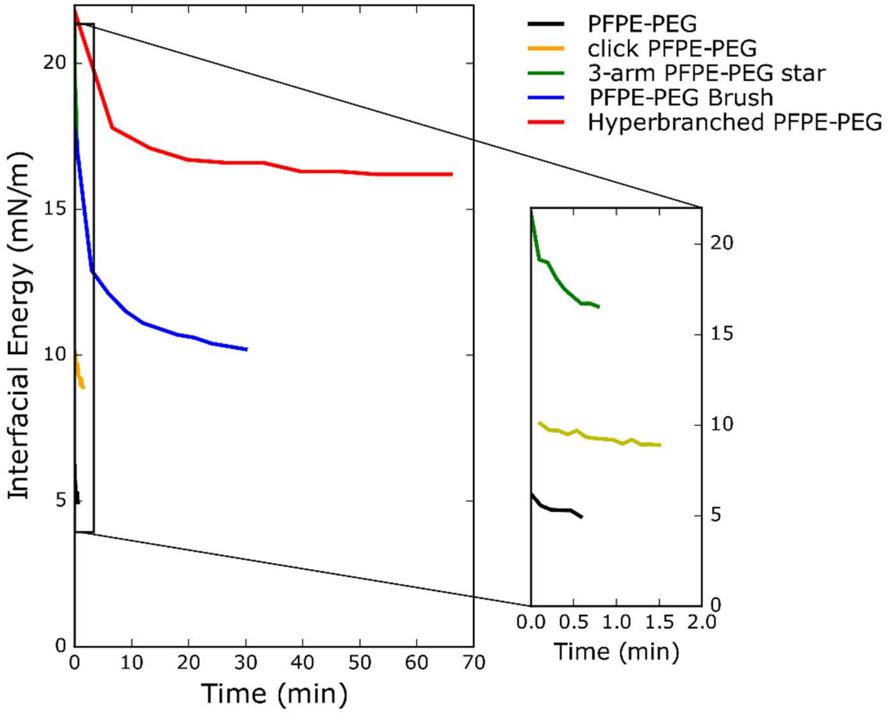

Supplemental Figure 1. Kinetics of interfacial energy equilibrium for each surfactant at 3% wt. in HFE 7500.

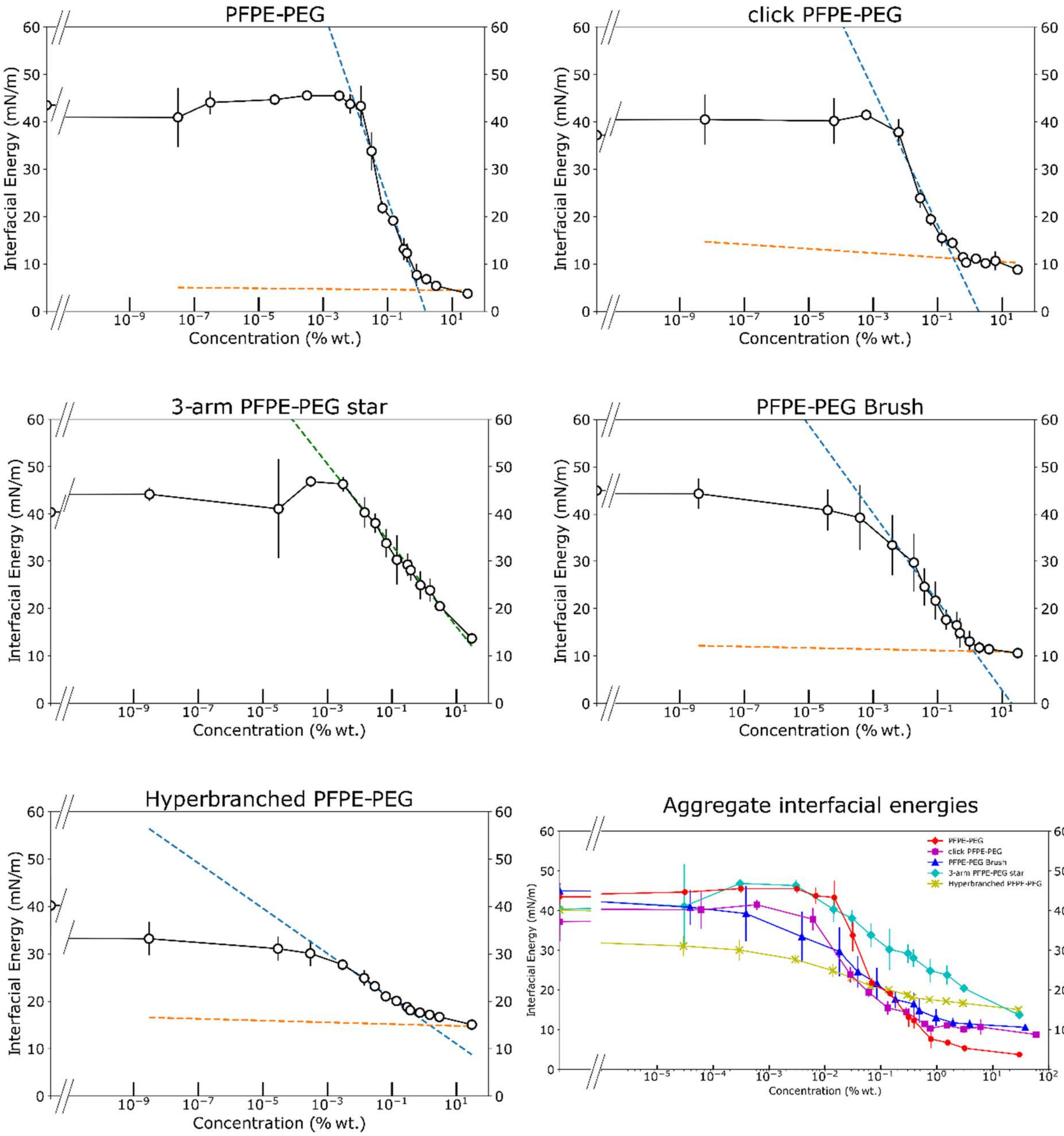

Supplemental Figure 2. Determination of cmc values of the different surfactants in HFE 7500. We used direct fitting of the decreasing slope and plateau to obtain their intersection that determines the cmc value. Surfactant concentrations range from 10^{-9} to about 30% in weight and their associated interfacial energies were determined with the pendent drop method after reaching the equilibrium determined in Supplemental Figure 1.

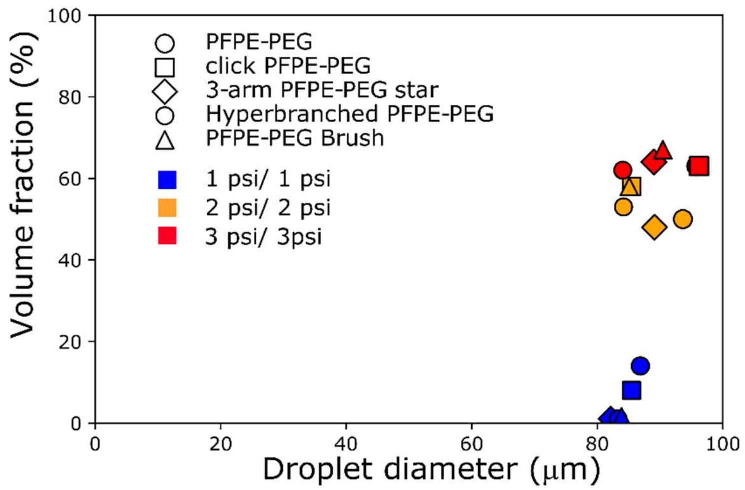

Supplemental Figure 3. Characterization of the generation of PBS containing droplets with the different surfactants dissolved at 3% weight in HFE 7500 for three characteristic pressure sets used to flow the oil and the aqueous phases.

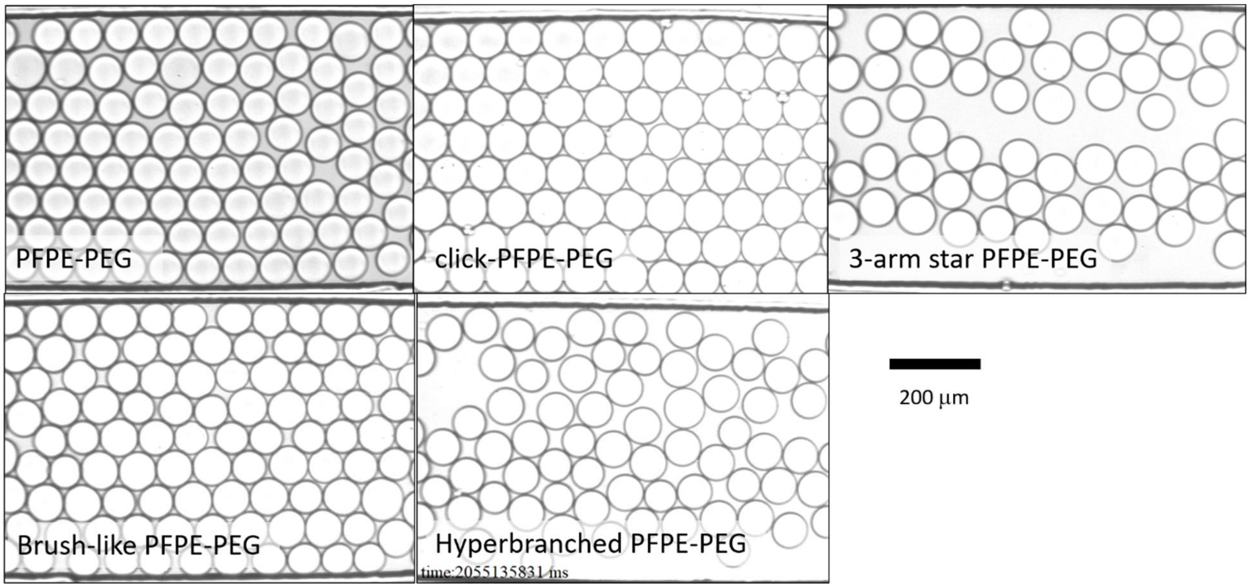

Supplemental Figure 4. Representative images of emulsions containing a PBS buffer stored at room temperature for 30 days. Each surfactant was dissolved at 3 % wt. in HFE 7500.

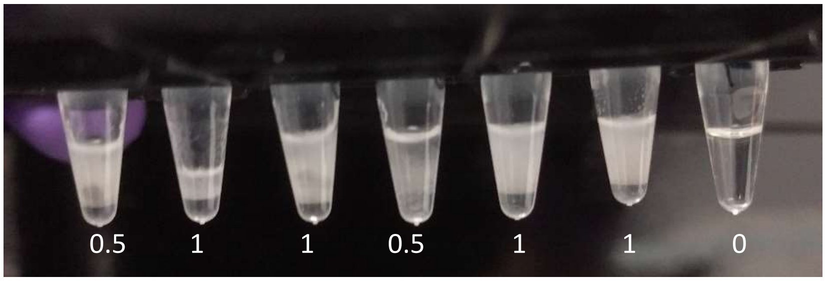

Supplemental Figure 5. Qualitative grading of emulsion stability. This picture displays the outcome of one a PCR reaction and serves as reference for emulsion stability scoring an example of scoring. From left to right: In order (with corresponding score underneath), emulsion after PCR reaction containing Tetronic 1307 in HFE 7500 with 3% wt. dissolved of 1) brush-like PFPE-PEG, 2) click PFPE-PEG, 3) 3-arm star PFPE-PEG, 4) hyperbranched PFPE-PEG, 5) PFPE-PEG, 6) PFPE-PEG PCR negative control, 7) no emulsion PCR positive control. Corresponding stability score shown underneath each tube.

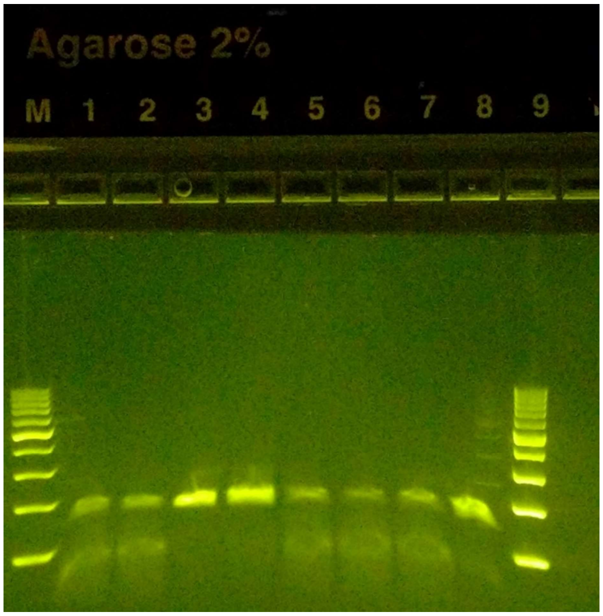

Supplemental Figure 6. Example of an electrophoresis of HDA products amplified from plasmid KRAS-PCR2.1 using the IsoAMP II Universal tHDA system and different surfactants. Lanes 3, 4 and 8 show a robust amplification of a 150-bp target sequence, while lanes 1,2, 5, 6 and 7 reveal a sub-optimal 150 bp amplification product with primer-dimer products (lower band, below 100 bp). The molecular weight marker is a 100-bp DNA ladder (Gene ruler, ThermoFisher).

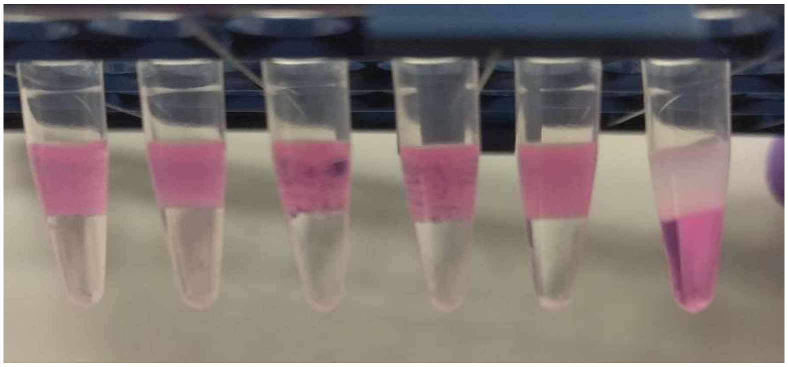

Supplemental Figure 7. Test of partitioning of Sytox orange, a DNA staining dye. The dye gives a pink coloration to the phase it resides in. Molecular transport would be indicated by a pink coloration of the oil phase (bottom phase). Data show an absence of molecular transport into the fluorinated phase in the case of the novel surfactants (tubes 1 through 5, see below). From left to right, the emulsions are made in HFE 7500 and 1) PFPE-PEG, 2) click PFPE-PEG, 3) 3-arm star PFPE-PEG, 4) brush-like PFPE-PEG, 5) hyperbranched PFPE-PEG, and the 6) ammonium Krytox ionic surfactant used as a positive control.

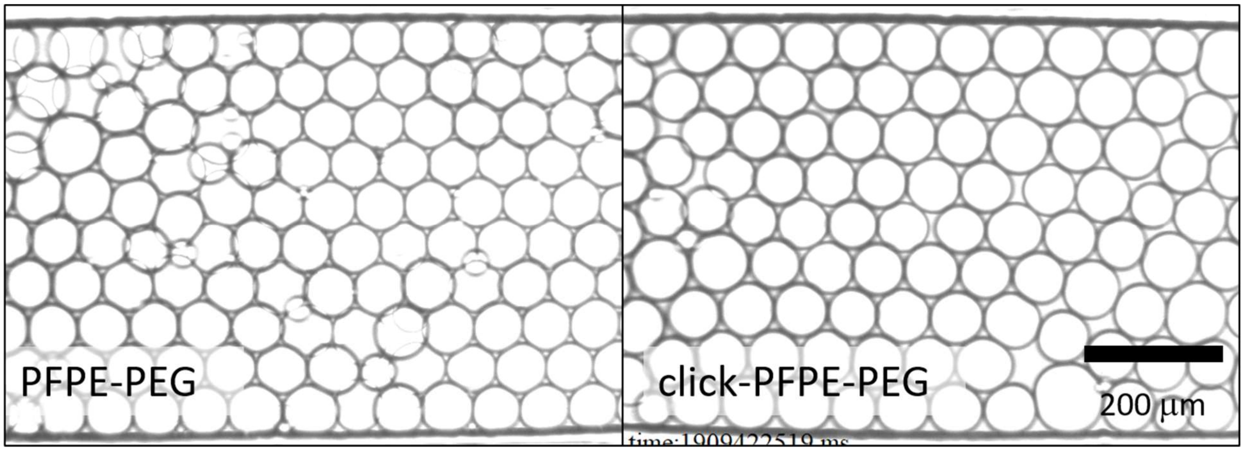

Supplemental Figure 8. Representative images of emulsions after isothermal HDA reaction. Each surfactant was dissolved at 3 % wt. in HFE 7500.

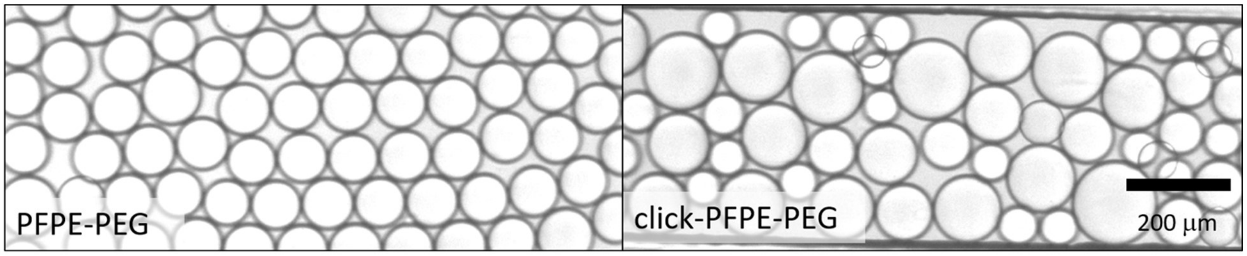

Supplemental Figure 9. Representative images of emulsions after Polymerase Chain Reaction (PCR). Each surfactant was dissolved at 3 % wt. in HFE 7500.

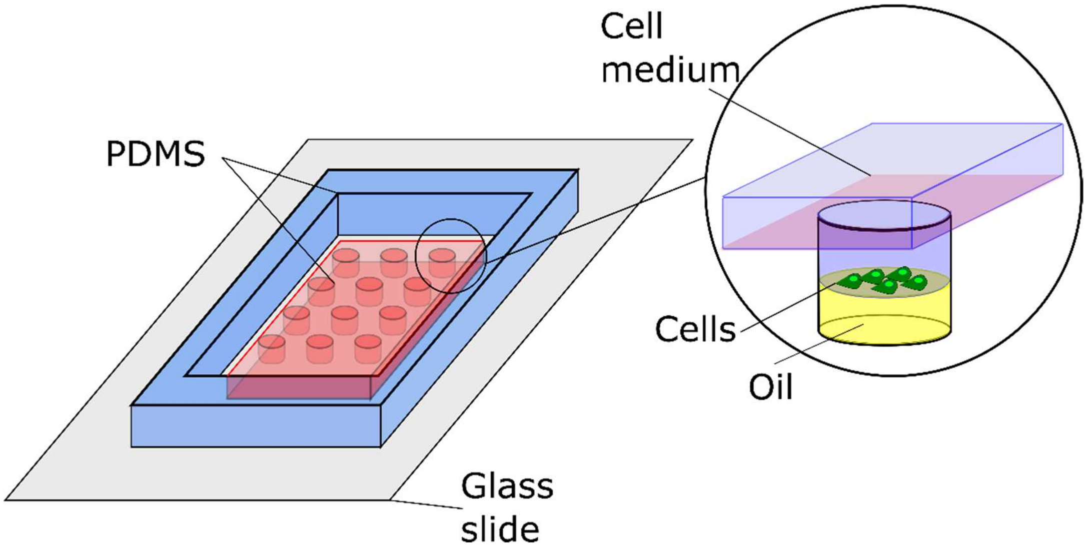

Supplemental Figure 10. Schematic of the PDMS chip to perform cell overlay experiments with very low volume of oil (10 μL). A series of 3 mm diameter microwells are punched into a PDMS slab (red) bonded to a glass slide (gray). An additional PDMS wall (blue) surrounds the central microwells to create a vast reservoir for the cell medium and avoid evaporation. The oil volumes (yellow) are first dispensed, followed by the cell medium (purple) and finally cell (green) seeding to obtain a homogenous number of cells across the microwells (insert).

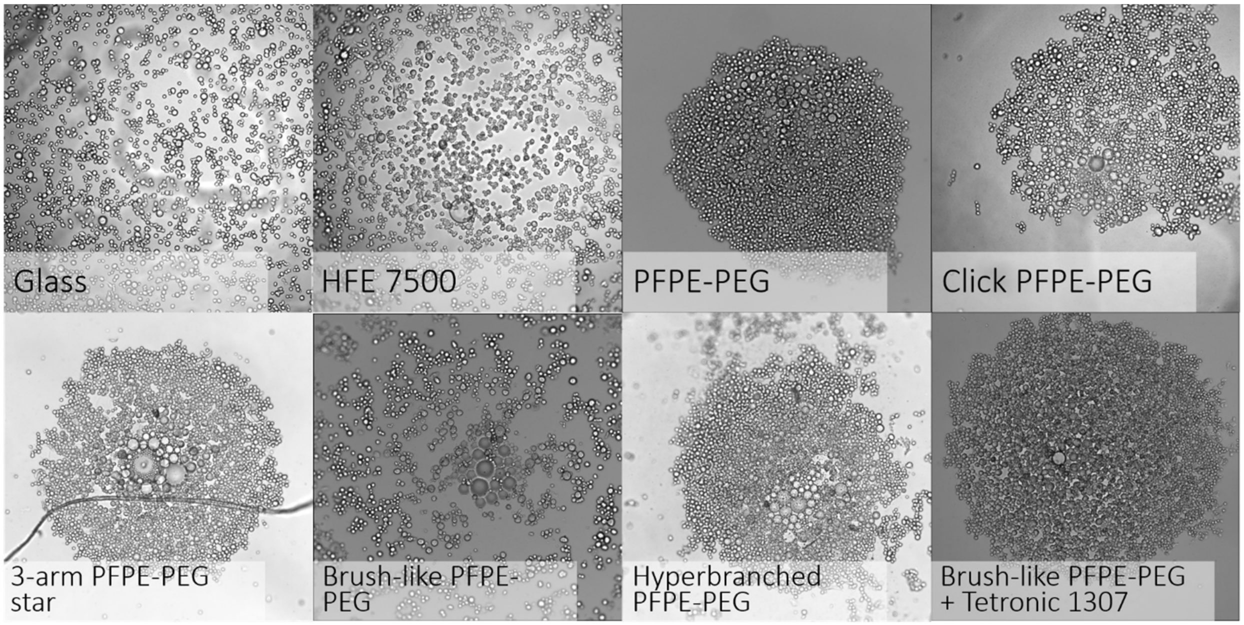

Supplemental Figure 11. CHO cells clustering in overlay experiments after 1 hr incubation on top of different materials.

Surfactant (3% wt. in HFE 7500)	No additive	Tetronic 1307
PFPE-PEG	0.125 (n=4)	0.94 (n=8)
Click PFPE-PEG	0 (n=2)	0.375 (n=4)
3-arm star PFPE-PEG	0 (n=2)	0 (n=3)
Brush-like PFPE-PEG	0 (n=2)	0.5 (n=4)
Hyperbranched PFPE-PEG	0 (n=2)	0 (n=3)
PFPE-PEG +<br>3-arm star PFPE-PEG	0 (n=3)	-
Click PFPE-PEG +<br>3-arm star PFPE-PEG	0.33 (n=3)	-
PFPE-PEG +<br>Brush-like PFPE-PEG	0 (n=3)	-
Click PFPE-PEG +<br>Brush-like PFPE-PEG	0.17 (n=3)	-
3-arm star PFPE-PEG +<br>Brush-like PFPE-PEG	0 (n=3)	-

Supplemental Table 1. Stability of emulsions after PCR reaction graded qualitatively (see **Supplemental Figure 1**) in presence or absence of Tetronic 1307. n indicates the number of repeats. See main text for quantitative assessment of some of the emulsions.

Supplemental Information 1- Experimental conditions and NMR characterization of the click based surfactant synthesis

PFPE acid chloride

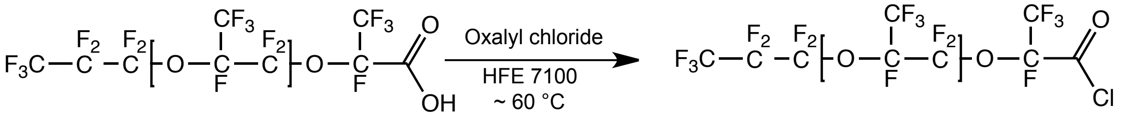

Reagent Table:

Name	MW	Moles/Equiv.	Amount used	Other (density, purity, safety, misc.)
Krytox FSH	5840	12.89 mmol (1 eq)	75.25 g	
Oxalyl Chloride	126.93	39.4 mmol (3.05 eq)	3.44-mL (5.00 g)	ρ = 1.455 g/mL; b.p. 62-65°C
HFE 7100	250.06		125-150 mL	b.p. 61°C; ρ = 1.52 g/mL

Precedent: J. Polym. Sci. B; **43** (2005) 3685.

Experimental Procedure: A flame-dried three-necked 14/20 RBF was loaded with 75.25 g of Krytox FSH (12.89 mmol), a 1” x 5/16” PTFE coated stirbar was added and the flask was then fitted with a rubber septum, a 14/20 thermometer adapter/thermocouple and a Liebig condenser. The reaction vessel was then thoroughly flushed with N2 before adding 150-mL of HFE 7100; the mixture was vigorously stirred to obtain a clear, colorless solution. Finally, 3.44-mL (5.0 g, 39.4 mmol, 3.05 equiv) of oxalyl chloride was added and the mixture was brought to reflux. After ca. 18-hrs, the solution was found to be turbid. Isolation of the acid chloride was then performed in-situ; the reaction vessel was fitted with a vacuum adapter under positive N2. The apparatus was then connected to a vacuum trap, the trap was cooled in (l) N2 and vacuum applied and the crude reaction mixture isolated in-vacuo under continued heating/stirring. A ca. 300 mg sample of the crude isolate was dissolved in 1.0 mL of 88:12 hexafluorobenzene:benzene-d6 and 19F NMR collected. On confirming complete conversion to the corresponding acid chloride the crude reaction product was dissolved in 90 mL of FC 3283 and filtered under inert conditions.

^{19}F NMR (282 MHz): -79.66, -80.16, -81.95, -82.57, -125.68, -130.48, -144.87.

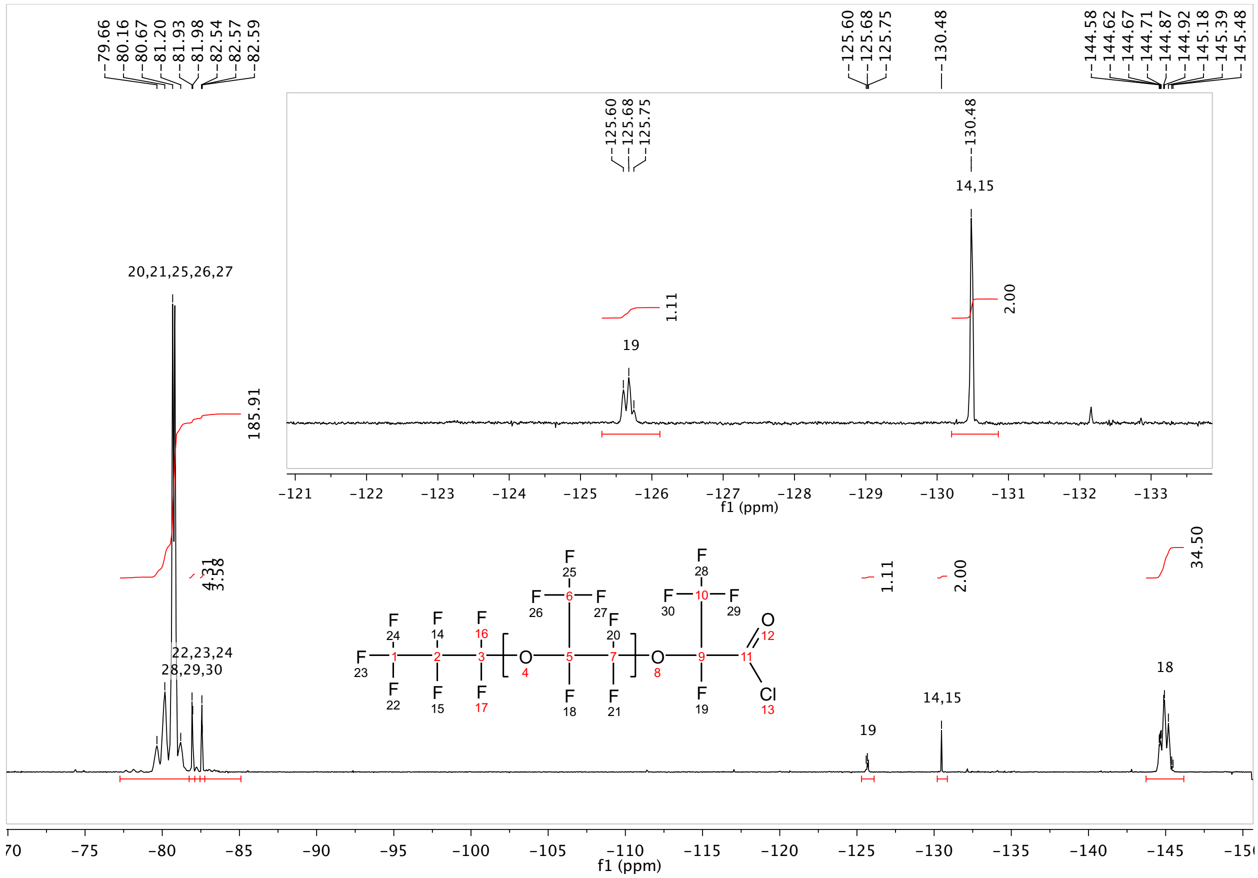

Kytox FSH PFPE acid chloride M_n determination: 34.5 repeats, 34.5 repeats x 166.02 g/mol repeat = 5730 g/mol; 5730 g/mol + 169.02 g/mol *(propyl tail)* + 179.47 g/mol *(acid chloride terminus)* = 6078.49 g/mol; M_n = 6080 g/mol

PFPE propargyl derivative

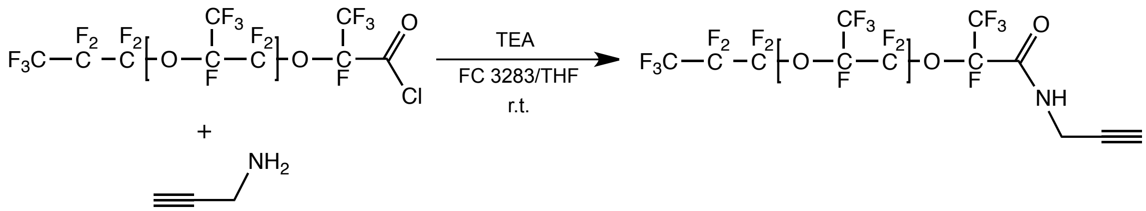

Reagent Table:

Name	MW	Moles/Equiv.	Amount used	Other (density, purity, safety, misc.)
PFPE acid chloride	5860	12.89 mmol (1 eq)	75.53 g	
Propargyl Amine	55.08	13.5 mmol (1.05 eq)	0.865-mL (0.744-g)	ρ = 0.860 g/mL; b.p. 83°C (lit.)
FC 3283	---	---	90-mL	b.p. 130°C, ρ = 1.82 g/mol
Tetrahydrofuran	72.11	---	35-mL	ρ = 0.889 g/mL at 25°C (lit.) ; b.p. 65-67°C (lit.)
Triethylamine	101.19	19.3 mmol (1.5 eq)	2.70-mL (1.96-g)	ρ = 0.726 g/mL at 25°C (lit.) ; b.p. 88.8°C (lit.)

Experimental Procedure:

A 250 mL 3-necked 14/20 RBF equipped with a 5/16" x 1" PTFE coated stir bar, was fitted with a 60 mL addition funnel, a 14/20 glass stopper and rubber septum; assembled apparatus flame dried under vacuum. The previously generated PFPE acid chloride/FC 3283 solution was then loaded into the reaction vessel addition funnel. A solution consisting of 2.7 mL (19.3 mmol, 1.96 g, 1.5 equiv.) TEA, 0.865-mL (13.05 mmol, 0.744 g, 1.05 equiv.) propargyl amine and 35-mL THF was then prepared in a flame-dried 50-mL 14/20 RBF fitted with a rubber septum under N2. The resultant solution was then transferred to the 250-mL reaction vessel under inert atmosphere. To this solution was added the acid chloride/FC 3283 solution under vigorous stirring; the reaction mixture was noted to emulsify immediately. After ca. 18-hrs the crude reaction mixture was transferred to a 250 mL 24/40 RBF and evaporated to dryness by means of rotary evaporation. The as isolated crude oil was then redissolved FC 3283 and filtered before concentrating once more to obtain a viscous, clear yellow oil. 1H and 19F NMR were then collected at ca. 300 mg/mL in 88:12 HFB:C6D6.

^{1}H NMR (300 MHz, Benzene): 6.53, 4.07, 2.13.

^{19}F NMR (282 MHz): -79.66, -80.17, -82.54, -83.72, -83.77, -130.47, -133.40, -144.90.

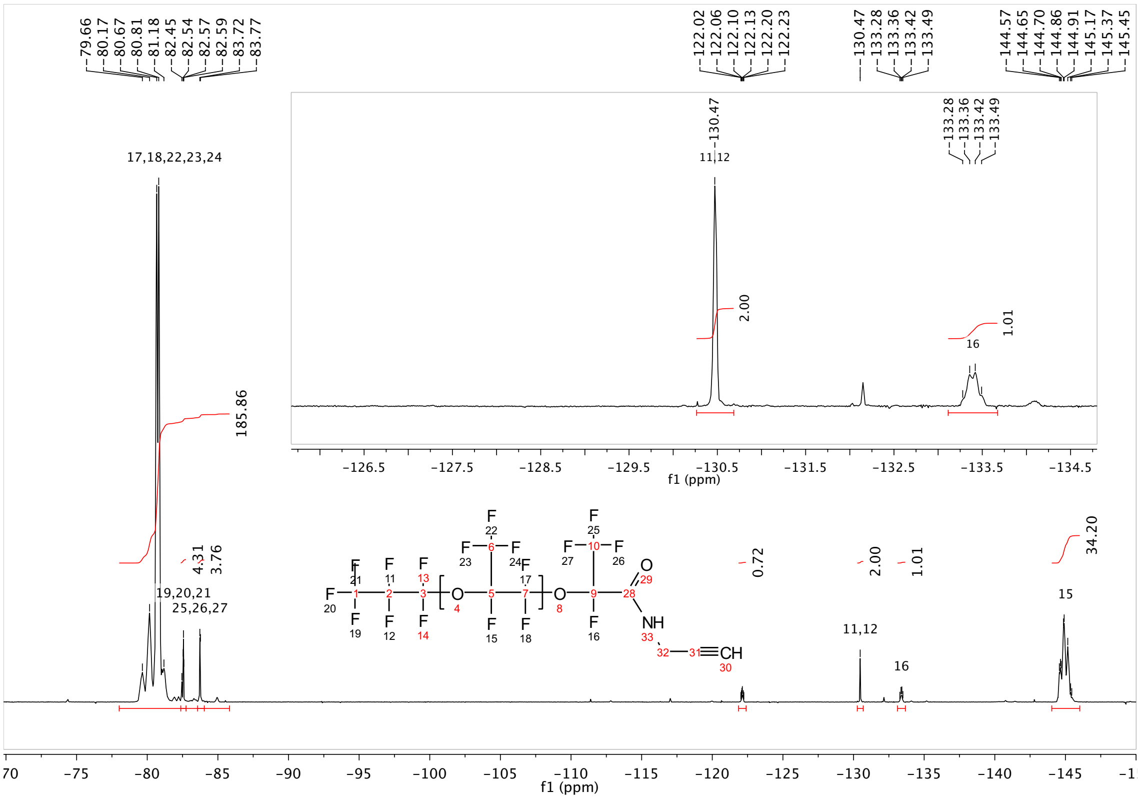

Kytox FSH PFPE propargyl derivative Mn determination: 34.2 repeats x 166 g/mol repeat = 5680 g/mol; 5680 g/mol + 169 g/mol *(propyl tail)* + 198.1 g/mol *(amide linked propargyl terminus)* = 6047.45 g/mol; M_n = 6050 g/mol ***Note:*** Peak(s) -122.02, -122.06, -122.10, -122.13, -122.20, -122.23 correspond to perfluorotripropylamine (FC 3283)

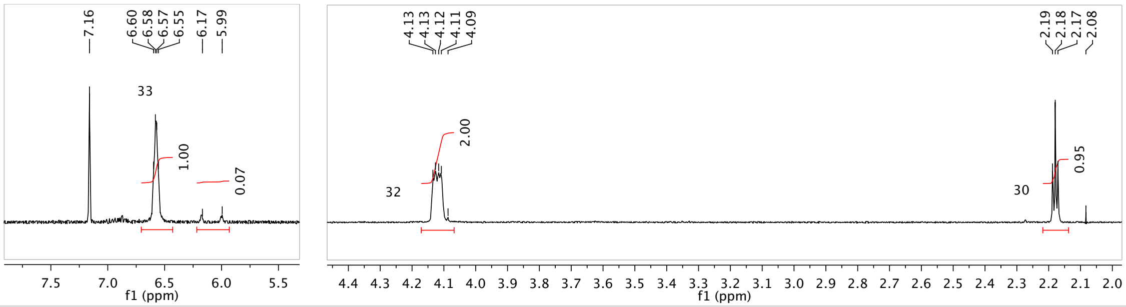

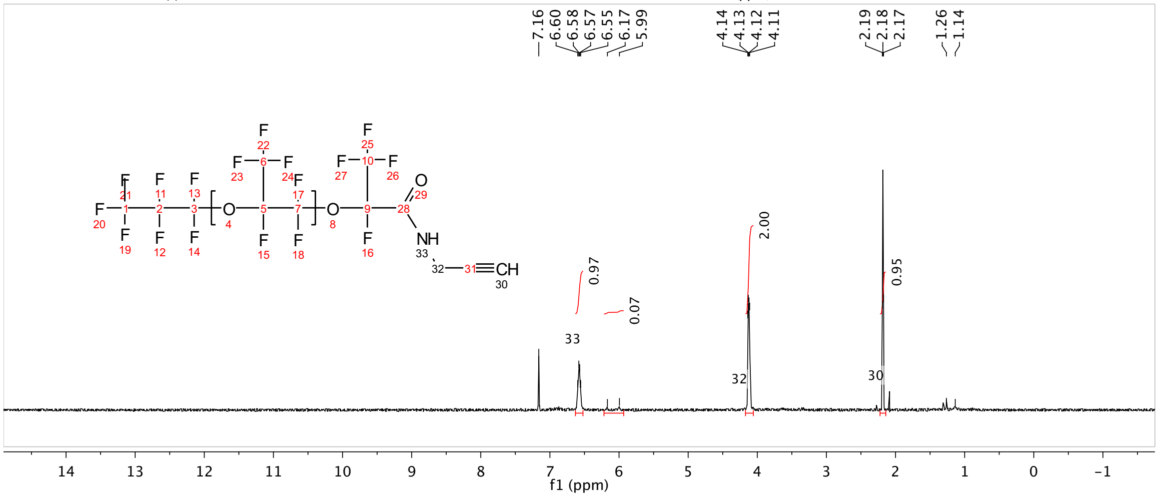

PEG 600 ditosylate

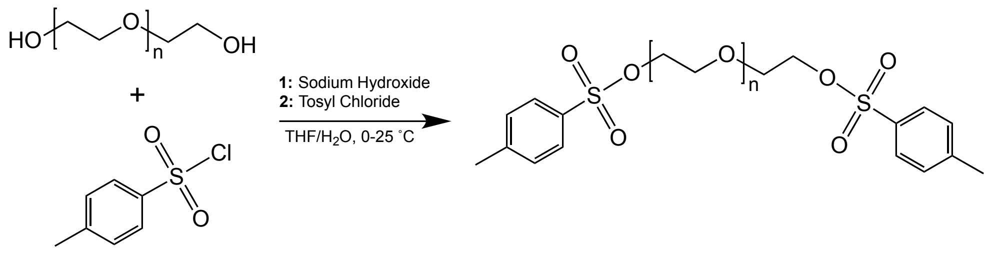

Reagent Table:

Name	FW	Moles/Equiv.	Amount used	Other (density, purity, safety, misc.)
Poly(ethylene glycol)	~600	0.0833 mol (1 eq)	50.0 g	
p-Toluene Sulfonyl chloride	190.65	0.191 mol (2.3 eq)	36.41 g	
Tetrahydrofuran	---	---	420-mL	b.p. 66°C
Water	---	---	102.5-mL	
Sodium Hydroxide	39.997	0.333 mol (4.0 eq)	13.32-g	~ 3.25 M
Brine	---			

Experimental Procedure:

13.32 g (0.333 mol; 4.0 eq) of NaOH was dissolved in 102.5 mL H_2O (3.25 M). Solution cooled in an ice-bath to ~ 0°C; once cooled, a solution of 50.0 g (0.0833 mol; 1 eq, 2 eq of hydroxyl) PEG 600 diol in 200 mL THF was added drop-wise (maintained T ≤ 5°C). After completing addition reaction mixture was stirred while warming to r.t. (t ≈ 1 hr). After ~1-hour, the reaction mixture was once again cooled to ~0°C and a solution of 36.41 g (0.191 mol; 2.3 eq) tosyl chloride in 220 mL THF was added drop-wise (T ≤ 5°C). The reaction was left to stir while warming to room temperature for ca. 18 hrs. The reaction mixture was then transferred to a 1.0-L separatory funnel, aqueous and organic layers were separated. THF was evaporated from organic layer under reduced pressure (30" Hg vacuum) to obtain 75.27 g of a viscous milky-white liquid. The crude product was then dissolved in 750 mL EtOAc and washed with 125-mL DI H_2O 2x, followed by 100 mL of brine, isolated and dried over $MgSO_4$ for ~1-hr. Finally, the product was evaporated to dryness in-vacuo via rotary evaporation to obtain a viscous, clear oil.

^{1}H NMR (300 MHz, $CDCl_3$): 7.77, 7.31, 4.12, 3.66, 3.60, 2.42.

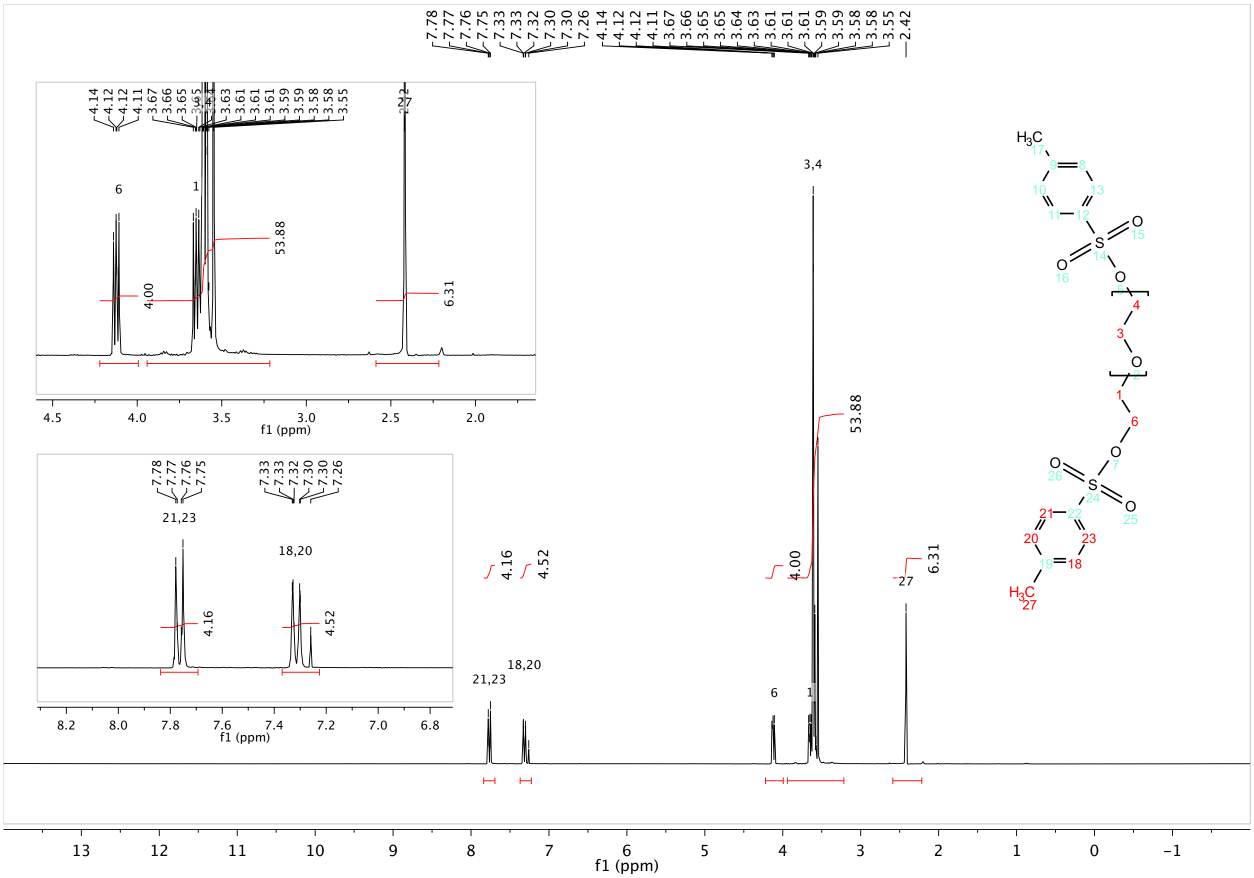
21,23
18,20
4.16
4.52
6
1
3,4
4.00
53.88
27
6.31
f1 (ppm)

PEG 600 diazide

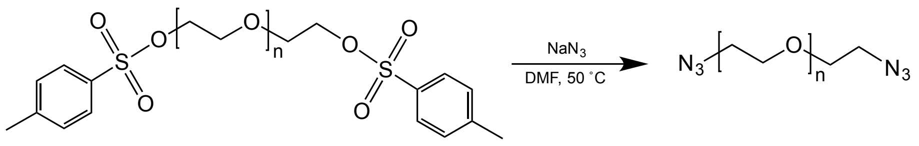

Reagent Table:

Name	FW	Moles/Equiv.	Amount used	Other (density, purity, safety, misc.)
PEO 600 ditosylate	865	0.0058 (1 eq)	5.00-g	
Sodium Azide,	65.01	0.0128 mol (2.2 eq)	0.832 g	
Dimethylformamide	---	---	15-mL	

5.00-g (5.8 mmol, 1 equiv) PEO 600 ditosylate was transferred to a 20 mL vial, followed by 15-mL of DMF and ½" x 5/16" PTFE coated stir bar and mixed. Finally 832-mg of sodium azide was added as a solid in a single portion and the reaction mixture stirred at r.t. ca. 90-min before heating to 50 C for ca. 18-hrs. After ~18 hrs the crude reaction mixture was noted to be a fine, white suspension. The suspension was then filtered filtrate to obtain a clear, homogeneous yellow-brown filtrate. DMF was removed by rotary evaporation and the resultant crude oil triturated with 100-mL of EtOAc. The mixture was then sonicated for ~15-minutes and filtered using 30-mL medium fritted glass funnel, the filtrate was noted to be clear and mostly colorless. The solution was then washed twice with 10-mL DI H2O and once with 10-mL brine; the collected organic layer was dried overnight over MgSO4.

^{1}H NMR (300 MHz, $CDCl_3$): 3.66, 3.63, 3.37.

^{13}C NMR (75 MHz, $CDCl_3$): 70.84, 70.24, 50.89.

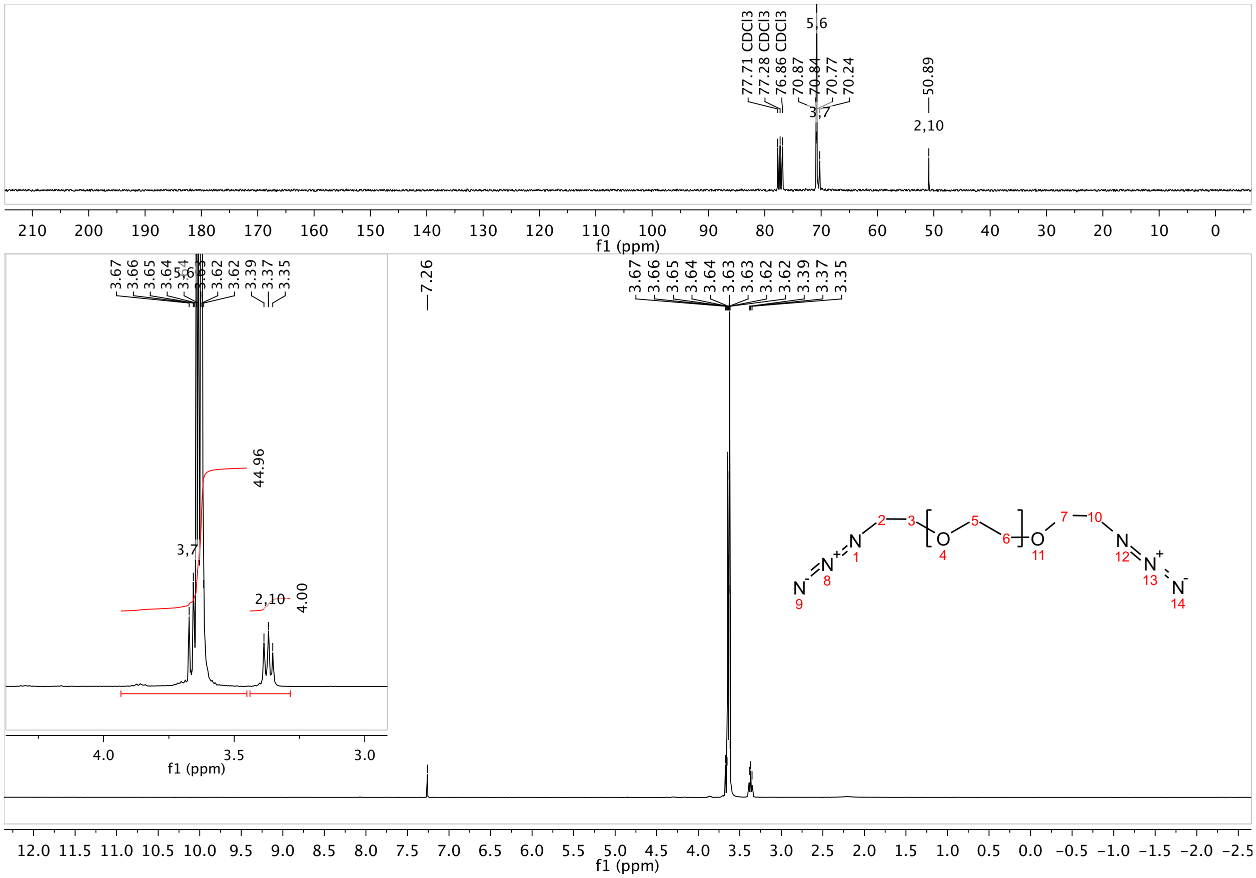
77.71 CDCl3
77.28 CDCl3
76.86 CDCl3
70.87
70.84
70.77
70.24
50.89
5,6
3,7
2,10
f1 (ppm)
3.67
3.66
3.65
3.64
3.64
3.63
3.63
3.62
3.62
3.39
3.37
3.35
7.26
44.96
4.00
f1 (ppm)

Glycerol ethoxylate tritosylate

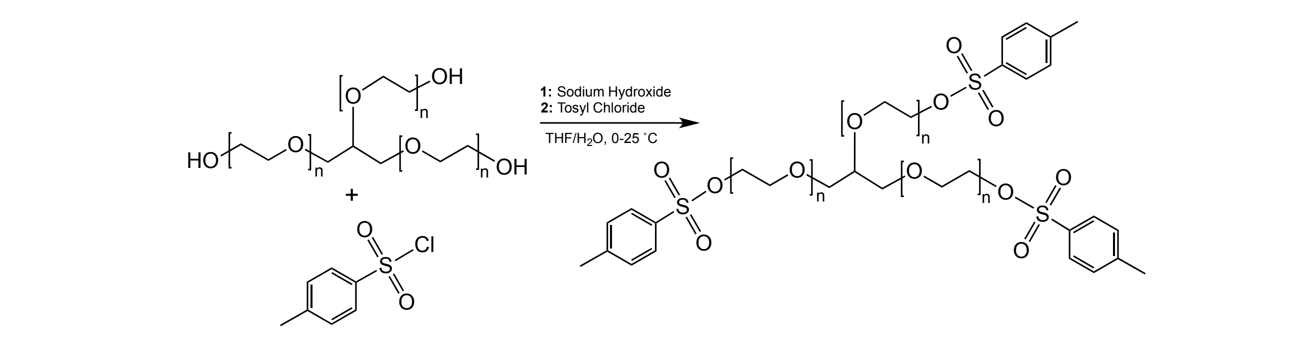

Reagent Table:

Name	FW	Moles/Equiv.	Amount used	Other (density, purity, safety, misc.)
Glycerol Ethoxylate, Average Mn = 1000	~1000	0.020 mol (1 eq)	20.0 g	
p-Toluene Sulfonyl chloride	190.65	0.069 mol (3.45 eq)	13.15 g	
Tetrahydrofuran	---	---	250-mL	b.p. 66°C
Water	---	---	25-mL	
Sodium Hydroxide	39.997	0.08 mol (4.0 eq)	3.20-g	~ 3.25 M

3.20 g (0.08 mol; 4.0 equiv.) of NaOH was dissolved in 25 mL H_2O (3.25 M); solution cooled in an ice-bath to ~ 0°C. Once cooled, a solution of 20.0 g (0.020 mol; 1 eq, 3 equiv.) glycerol ethoxylate in 125 mL THF was added drop-wise (T ≤ 5°C). After completing addition reaction mixture was stirred while warming to r.t. for ca. 1 hr. The reaction mixture was then once again cooled to ~0°C and a solution of 13.15 g (0.069 mol; 3.45 eq) tosyl chloride in 125 mL THF added drop-wise (T ≤ 5°C). The reaction was left to stir ca. 18 hrs while warming to room temperature. The crude reaction mixture was then allowed to phase separate and the organic layer separated. The separated organic layer was transferred to a 24/40 500-mL RBF and concentrated via rotary evaporator to obtain a clear, colorless oil. The crude oil was dissolved in 300-mL EtOAc and washed twice with 50-mL H_2O followed by 50-mL of brine before drying over $MgSO_4$ for approximately ca. 1 hr. Finally, the product was isolated under reduced pressure by rotary evaporation to obtain a viscous, clear, yellow oil.

^{1}H NMR (300 MHz, $CDCl_3$): 7.75, 7.73, 4.13, 4.11, 3.64, 2.40.

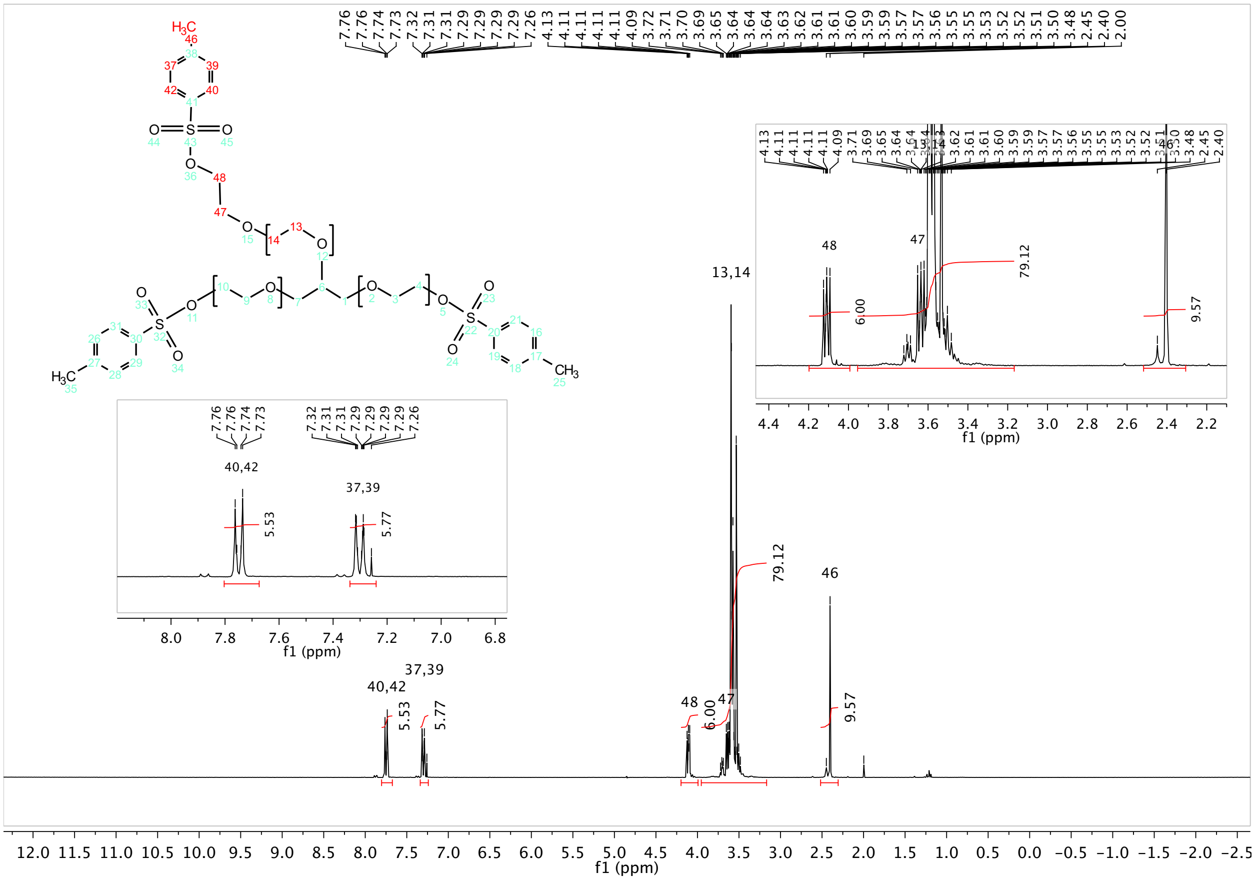
13,14
40,42
37,39
48
47
46
f1 (ppm)

Glycerol ethoxylate triazide

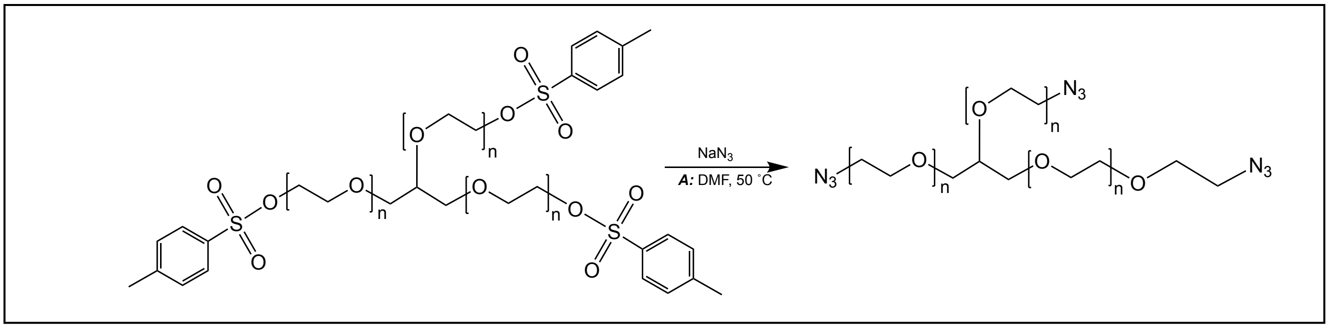

Reagent Table:

Name	FW	Moles/Equiv.	Amount used	Other (density, purity, safety, misc.)
Glycerol Ethoxylate Tri-tosylate	1465	0.00341 (1 eq)	5.00-g	
Sodium Azide,	65.01	0.0113 mol (3.3 eq)	0.735 g	
Dimethylformamide	---	---	30-mL	

5.0-g (0.00341 mol, 1 equiv.) of glycerol ethoxylate tritosylate was added directly to a 100-mL three-necked 14/20 RBF. The flask was fitted with Liebig condenser, a thermocouple and adapter, a 5/16" x 1" PTFE coated stir bar. 30-mL of DMF was added and the resultant mixture stirred vigorously. 0.735-g (0.0113, 3.3 equiv.) of sodium azide was then added as a solid in a single portion. The vessel was then sealed and flushed with N2 before heating to 50 ºC for ca. 16-hrs. The crude reaction mixture was transferred to a 100-mL 24/40 RBF and DMF removed in-vacuo via rotary evaporation. The residue was taken up in 80-mL of EtOAc and vacuum filtered over a medium porosity glass frit; solids rinsed with and additional 25-mL of EtOAc. The EtOAc solution was transferred to a 250-mL separatory funnel and a further 30-mL of EtOAc added before washing twice with 30-mL of DI H_2O and once with 30-mL of brine. The organic layer was isolated and set aside, the aqueous layer was then extracted with 60-mL of EtOAc. The combined organic extracts were dried over $MgSO_4$ under N_2 for ca. 1 hr. Finally, the solution was filtered over 11 cm filter paper into a 250-mL 24/40 RBF and EtOAc removed via rotary evaporation.

^{1}H NMR (300 MHz, $CDCl_3$): 3.60, 3.33.

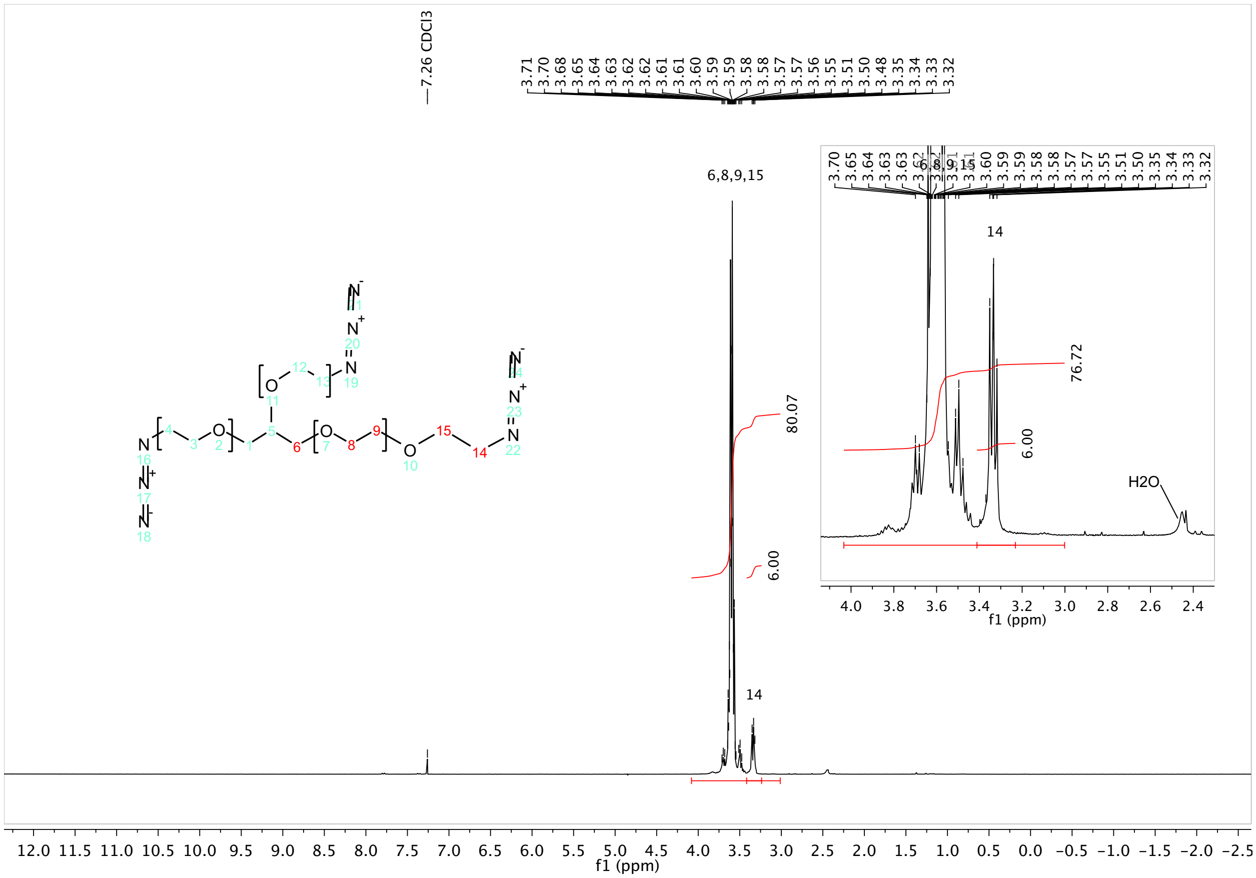
7.26 CDCl3
3.71 3.70 3.68 3.65 3.64 3.63 3.62 3.62 3.61 3.61 3.60 3.59 3.59 3.58 3.58 3.57 3.57 3.56 3.55 3.51 3.50 3.48 3.35 3.34 3.33 3.32
6,8,9,15
14
80.07
6.00
76.72
H2O
f1 (ppm)

Synthesis of Glycerol ethoxylate trithiol

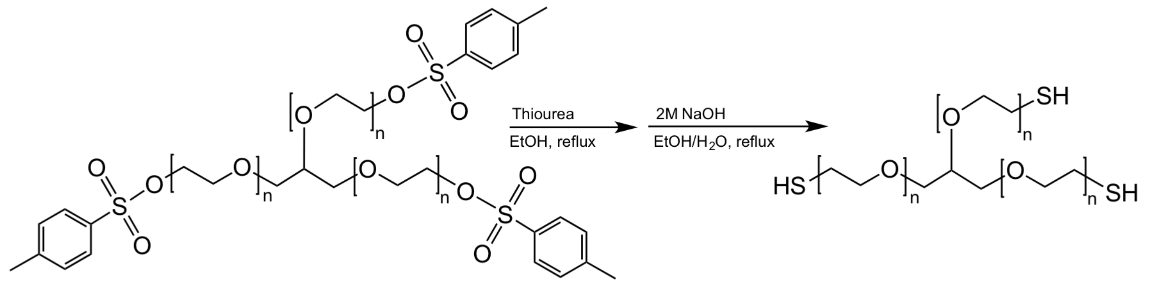

Reagent Table:

Name	FW	Moles/Equiv.	Amount used	Other (density, purity, safety, misc.)
Glycerol ethoxylate tritosylate	1465	0.00683 mol (1 eq)	10.0 g	
Thiourea,	76.12	0.0215 mol (3.15 eq)	1.64 g	
Ethanol	---	---	75-mL (1st volume) 38-mL (2nd volume)	
Sodium Hydroxide	39.997	0.0539 mol (2.63 eq)	2.156 g	
Water	---	---	3-mL	
HCl				

10.0 g (0.00683 mol; 1.0 eq) of glycerol ethoxylate tritosylate was added directly to a three-necked 14/20 RBF followed by 1.64-g (0.0215, 3.15 eq.) of thiourea as a solid. The flask was then equipped with a Liebig condenser, a thermocouple/adapter, a PTFE coated stir bar, and a septum. The system was evacuated under high-vacuum and backfilled for 2 cycles. Next, 75-mL of degassed 200-proof EtOH was added via cannulation under stirring, the resulting suspension/solution was then briefly stirred before being brought to reflux for ca. 24 hrs; steps were taken to protect the reaction vessel from light. After 24-hrs, a degassed solution of 2.156 g NaOH (0.0539 mol) and 38-mL EtOH was added and the mixture further refluxed for ca. 3 hrs before cooling to room temperature. Finally, a series of air-free liquid-liquid extractions employing THF provided the corresponding purified trithiol derivative.

^{1}H NMR (300 MHz, DMSO): 3.49, 3.39, 3.32, 2.59, 2.27.

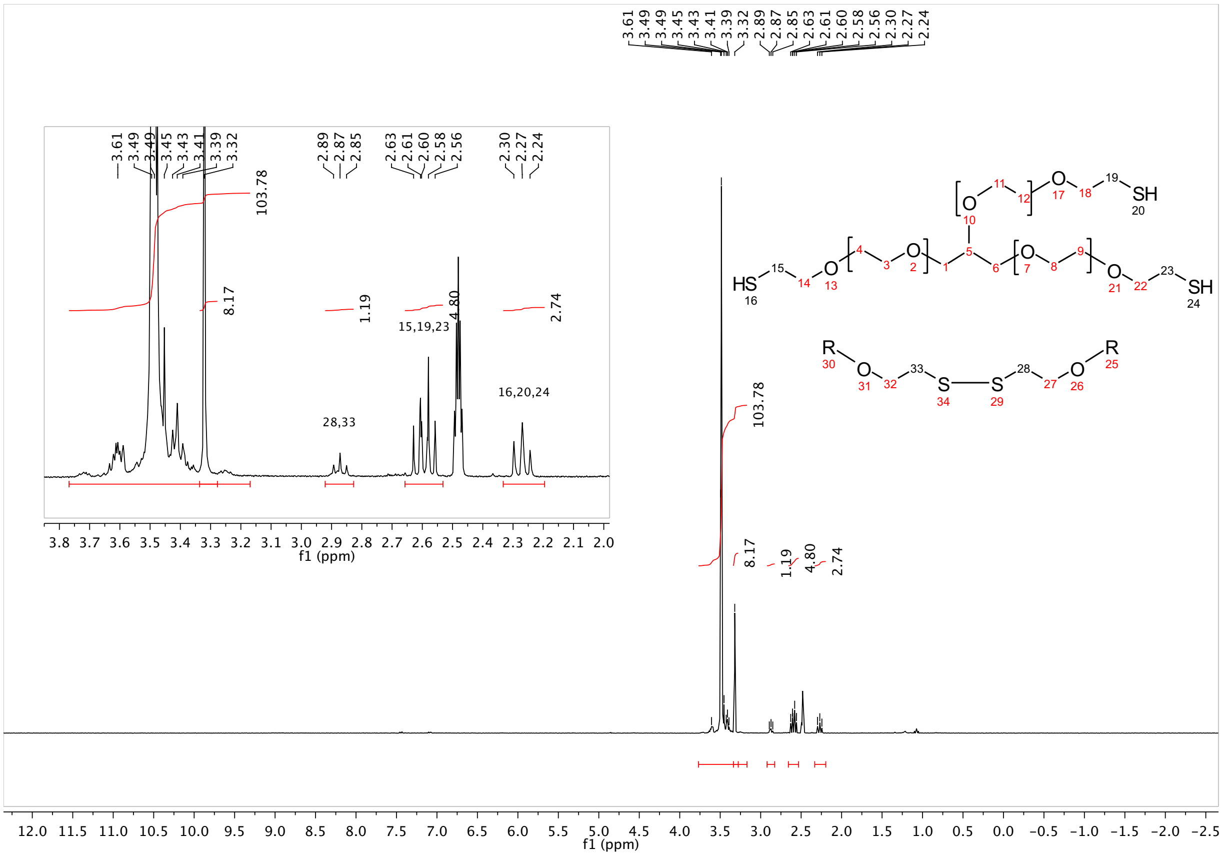
3.61 3.49 3.49 3.45 3.43 3.41 3.39 3.32 2.89 2.87 2.85 2.63 2.61 2.60 2.58 2.56 2.30 2.27 2.24
103.78
8.17
1.19
4.80
2.74
15,19,23
28,33
16,20,24
f1 (ppm)
HS
SH
SH
R
R
S
S
O
f1 (ppm)

PFPE-PEG triazole linked triblock

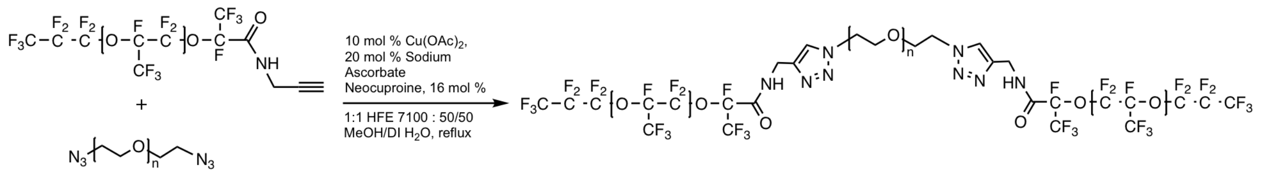

Reagent Table:

Name	FW	Moles/Equiv.	Amount used	Other (density, purity, safety, misc.)
Poly(HFPO) propargyl derivative	5900	0.51 mmol (2 eq)	3.0 g	(yield 2.263)
PEO-600-diazide	607	0.27 mmol (1.05 eq)	164-mg*	*Revised based upon M_n determination (derived from ^{1}H NMR integration)
Water	18.02	-	1.5-mL	
Methanol	-	-	1.5-mL	bp 64.7 °C, ρ = 0.791 g/mL
(+)-Sodium L-ascorbate	198.11	20 mol % (0.102 mmol)	20.2-mg	mp 220 °C
Neocuproine	208.26	0.0815-mmol 16-mol %*	17-mg	CAS # 484-11-7<br>mp 162 – 164 °C<br>soluble in EtOH, acetone, ether, benzene, slightly soluble in H_2O<br>*with respect to alkyne units
HFE 7100 (Methyl nonafluorobutyl ether)	250	-	3.0-mL	bp 61 °C, ρ = 1.5305 g/mL
Copper(II) acetate monohydrate	199.65	0.051 mmol 10-mol %*	0.0102-g	CAS # 6046-93-1,<br>*with respect to alkyne units<br>1.0 M in MeOH

Experimental Procedure:

3.0-g (0.51 mmol equiv.) of poly(HFPO) propargyl derivative was added to a 20-mL scintillation vial followed by 3.0-mL HFE 7100 and sonicated. 164 mg (0.27 mmol, 1.05 equiv.) of PEO 600 diazide was added to a 5-mL vial combined with 1.5-mL of MeOH and sonicated . Next,10.2 mg (0.051 mmol, 10 mol %) of Cu(II)OAc and 17 mg (0.0815 mmol, 16 mol%) were added to the PEO diazide/methanol solution and the mixture once again sonicated. A

solution of 20.2 mg (0.102 mmol, 20 mol %) sodium ascorbate in 1.5-mL DI H2O was generated and added to the PEO 600 azide/Cu(OAc)2/Neocuproine/MeOH solution. Finally, the azide/Cu/ligand/ascorbate solution was combined with the poly(HFPO) propargyl derivative/HFE 7100 solution in a 20 mL vial with a 1” x ½” PTFE coated stir bar and stirred vigorously at r.t. for ca. 1 hr before heating to 50 ˚C for 48-hrs.

^{1}H NMR (300 MHz, Benzene): 8.49, 7.90, 4.62, 4.49, 3.85, 3.56.

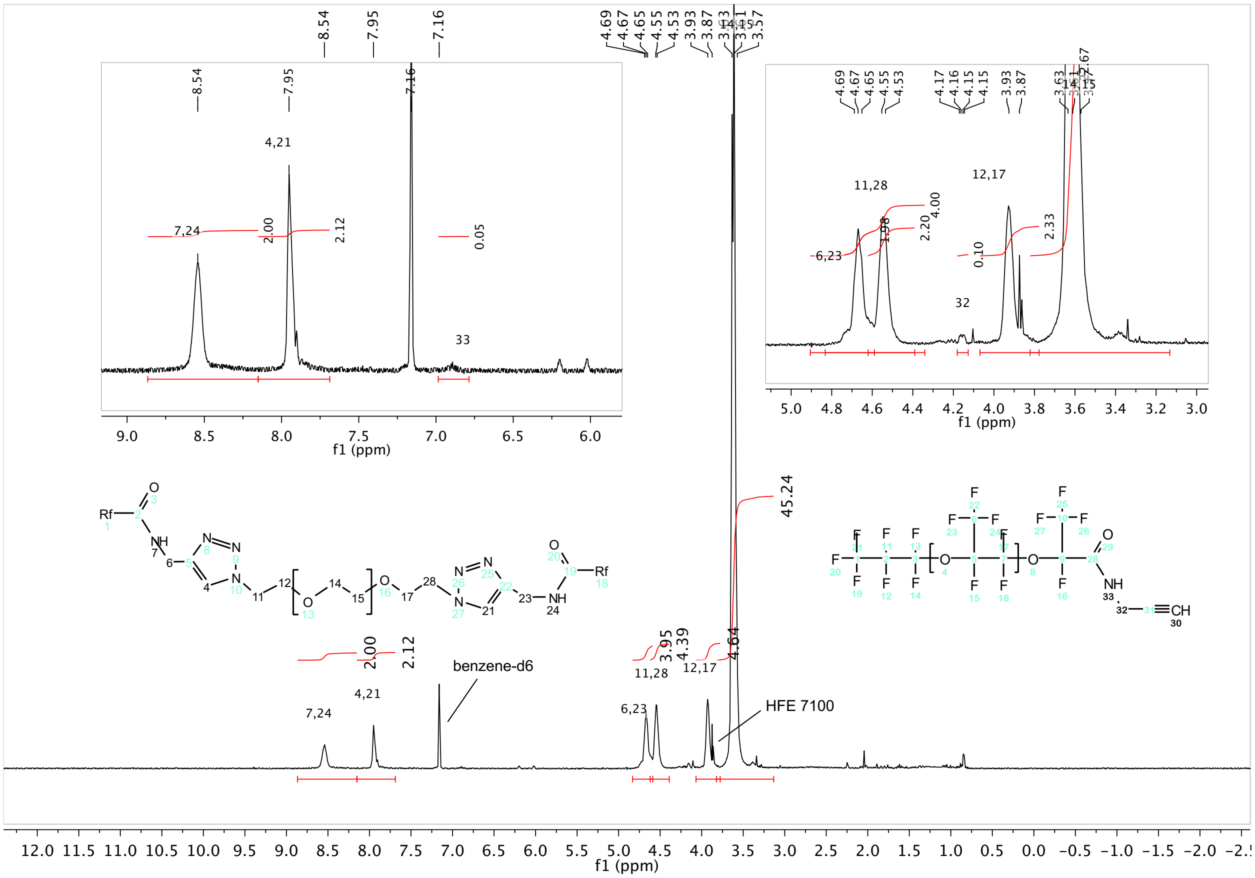

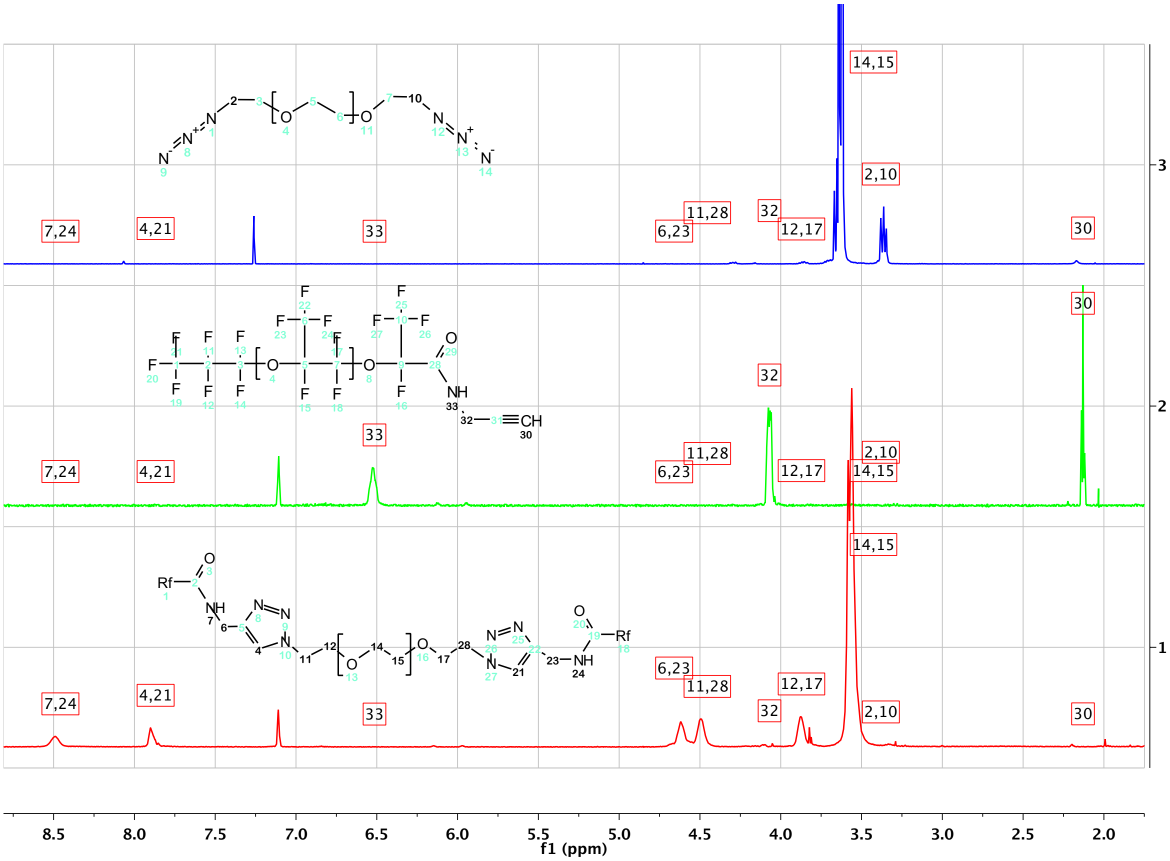

Note: Numbered assignments denote the position of a particular set of protons and not necessarily their presence.

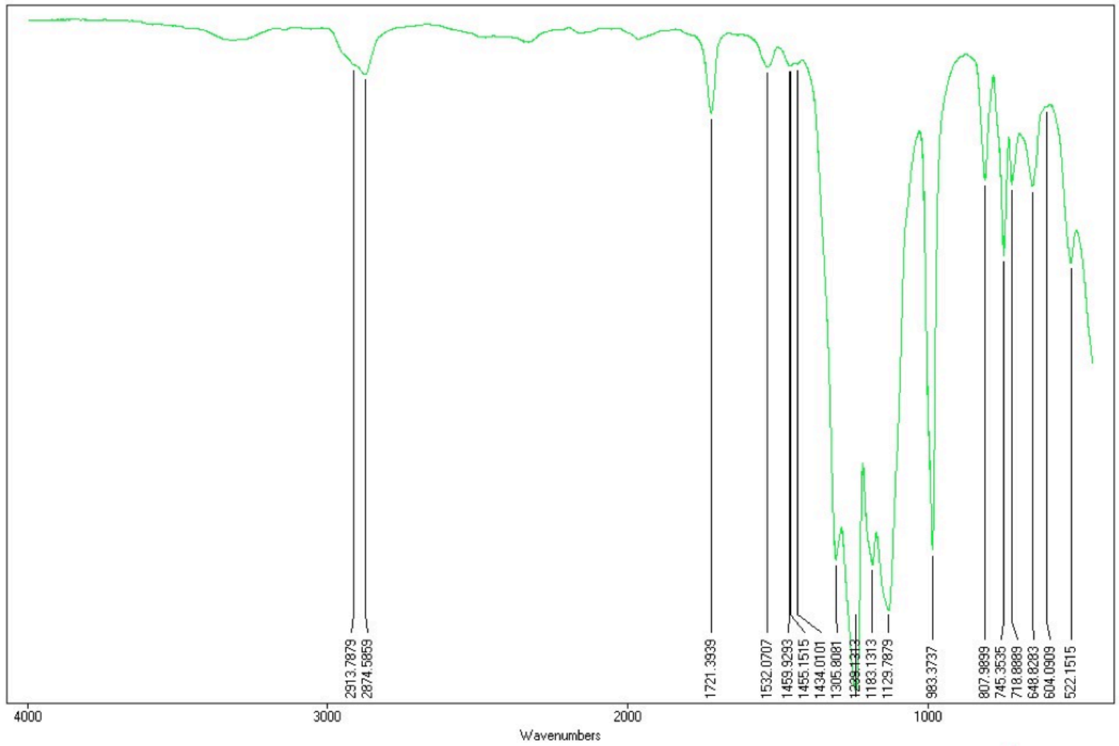

Brush-like PFPE-PEG

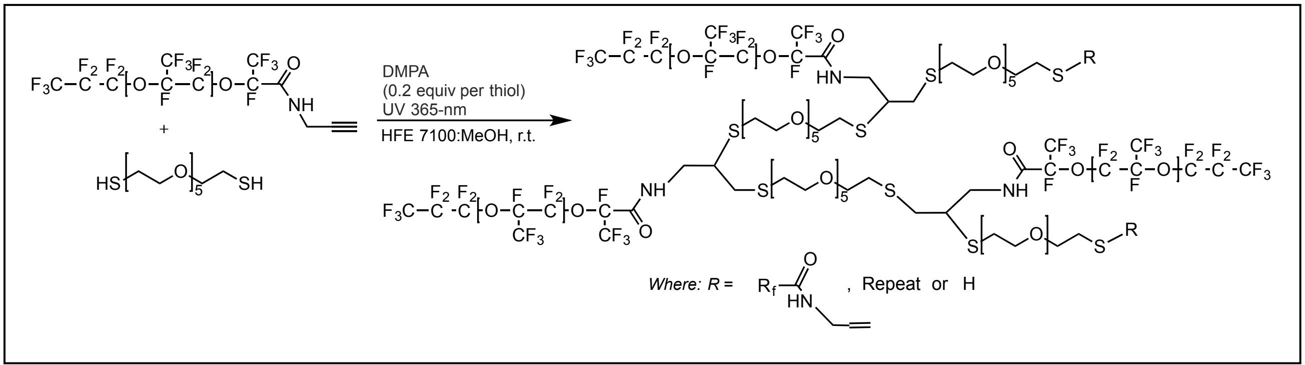

Reagent Table:

Name	FW	Moles/Equiv.	Amount used	Other (density, purity, safety, misc.)
Poly(HFPO) propargyl	5900	0.34 mmol (1 eq)	2.0 g	***Note(s):*** yield = 1.551
Methanol	-	-<br>-	3.5-mL	bp 64.7 °C, ρ = 0.791 g/mL
HFE 7100 (Methyl nonafluorobutyl ether)	250	-<br>-	7-mL	bp 61 °C, ρ = 1.5305 g/mL
2,2-Dimethoxy-2-phenylacetophenone	256.3	0.0068 mmol<br>0.2-eq *	17.6-mg	CAS # 24650-42-8<br><br>*relative to alkyne groups
Hexa(ethylene glycol) dithiol	314.46	0.34 mmol (1 eq)	106.8 mg<br>96-μL	bp 61 °C, ρ = 1.113 g/mL

Experimental Procedure:

2.0-g (0.34 mmol, 1 equiv) of PFPE-propargyl derivative was dissolved in 7-mL of methoxyperfluorobutane. Next, 106.8 mg (96-μL, 0.34 mmol, 1.0 equiv) of hexa(ethylene glycol) dithiol and 17.6 mg (0.068 mmol, 0.2 equiv) of 2,2-Dimethoxy-2-phenylacetophenone DMPA were combined and immediately dissolved in 3.5 mL of MeOH. The DMPA/thiol/MeOH solution was then transferred to the previously generated PFPE-propargyl derivative/HFE 7100 solution and the vial sealed with a septum. The sealed vessel was then flushed under positive pressure of N_2 and irradiated at 365-nm while stirring at ca. 1200-rpm, at r.t. overnight. The following day the crude reaction mixture was combined with an equivalent volume of MeOH (10.5 mL) and allowed to stand until complete phase separation was noted. The fluorous phase was then isolated and directly evaporated in-vacuo to obtain a clear, colorless oil.

^{1}H NMR (300 MHz): 8.29, 6.71, 6.33, 5.68, 3.61, 2.97, 2.82.

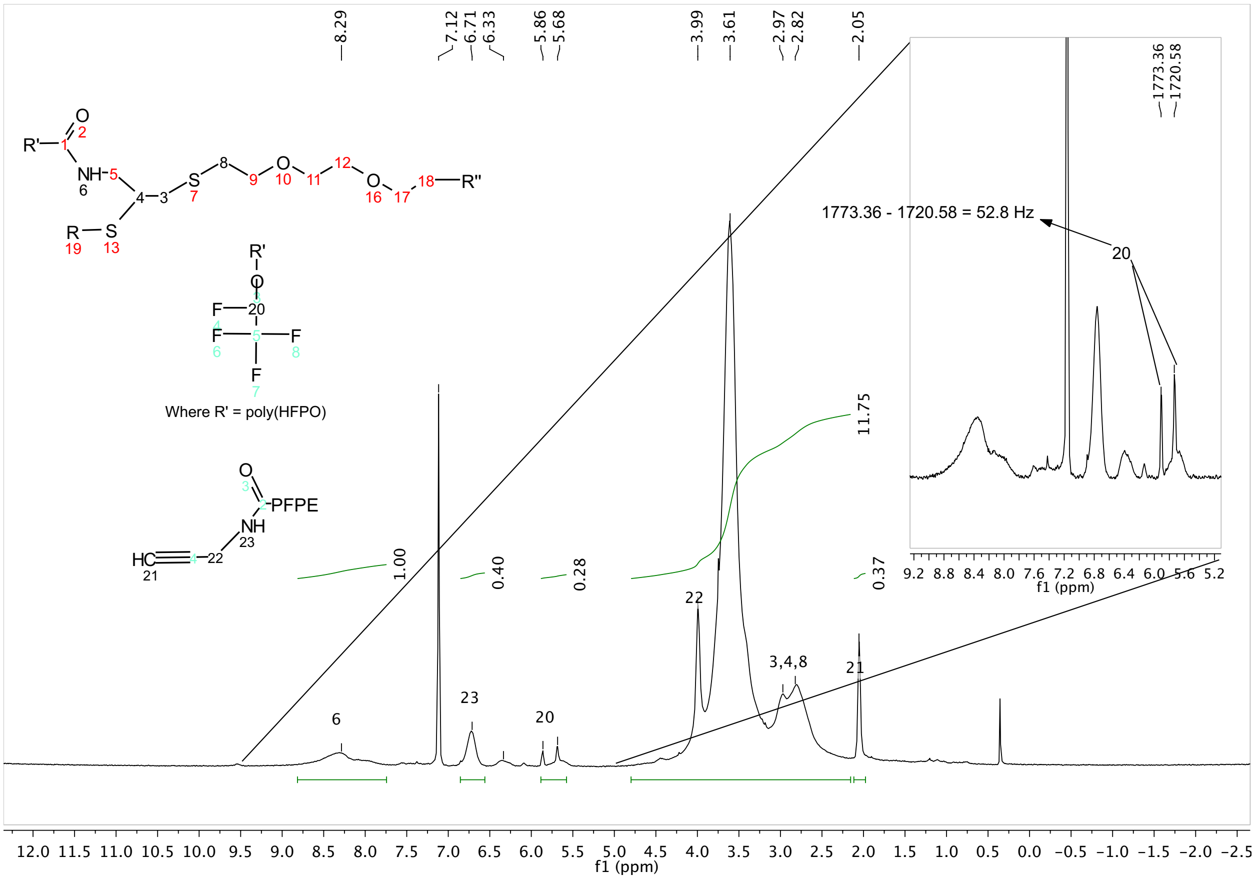
8.29
7.12
6.71
6.33
5.86
5.68
3.99
3.61
2.97
2.82
2.05
Where R' = poly(HFPO)
PFPE
1773.36 - 1720.58 = 52.8 Hz
1773.36
1720.58
1.00
0.40
0.28
11.75
0.37
f1 (ppm)

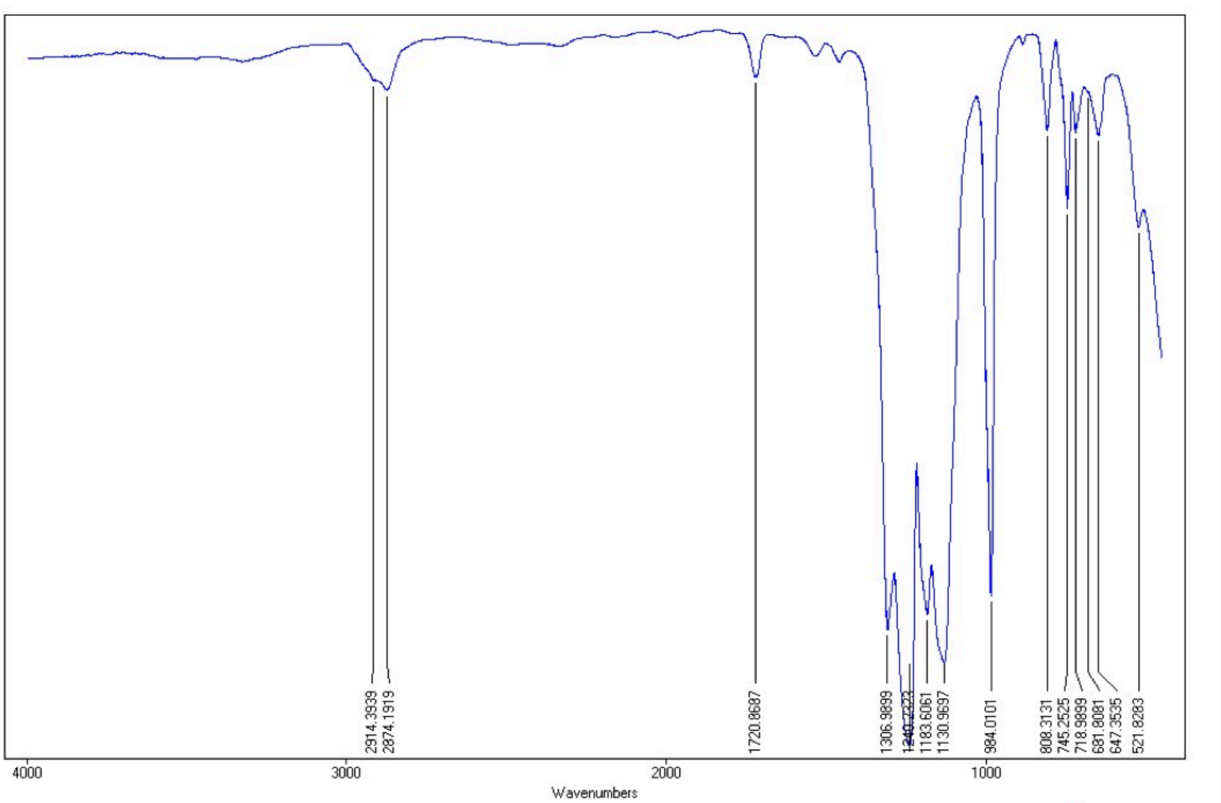
2914.3939
2874.1919
1720.8687
1306.9899
1240.2323
1183.6061
1130.9697
984.0101
808.3131
745.2525
718.9899
681.8081
647.3535
521.8283
Wavenumbers

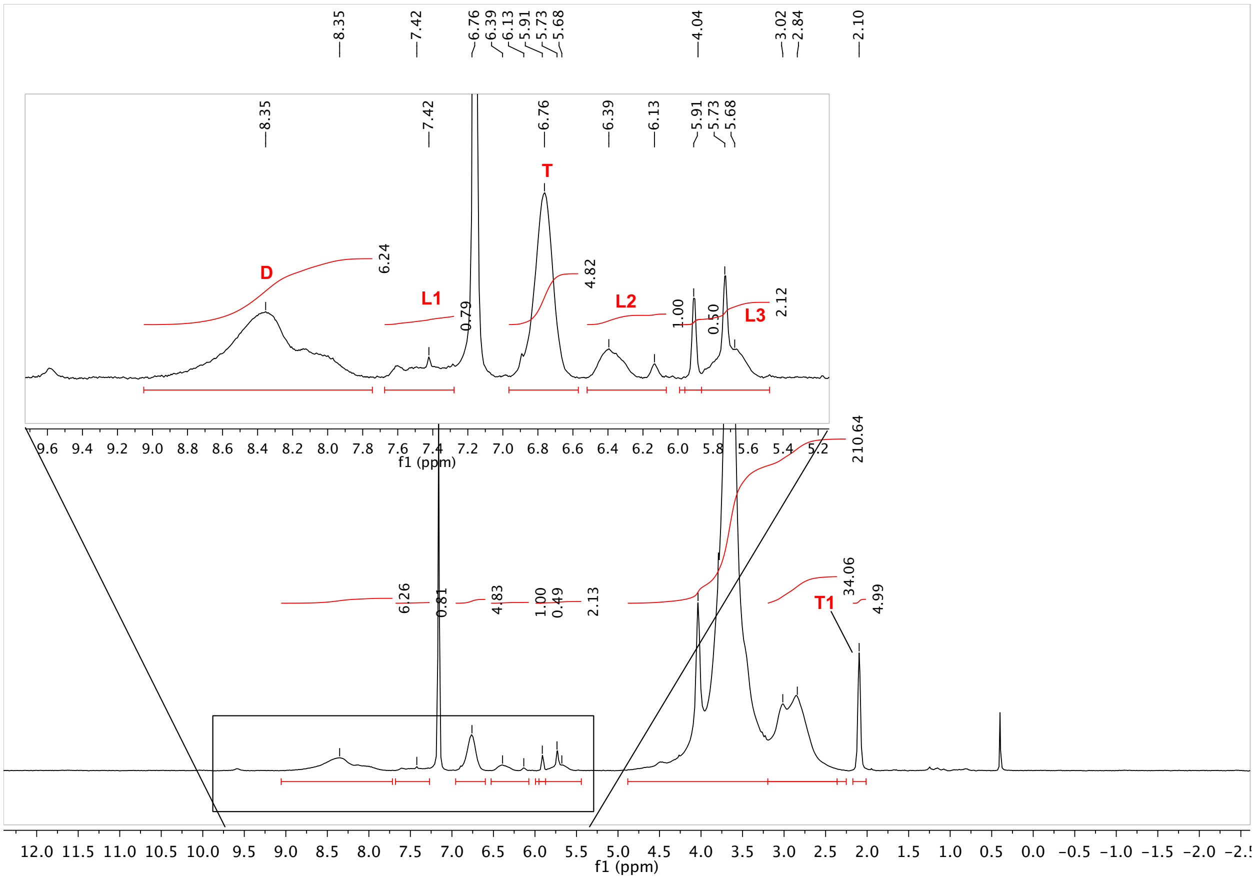

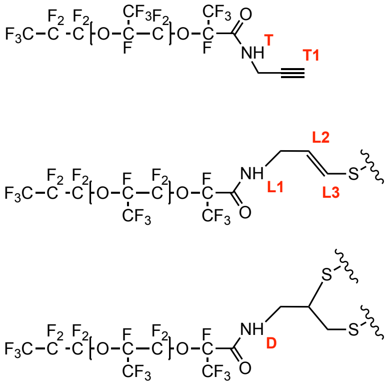

Brush-like sulfide linked PFPE-PEG (**13**): Substituting values: L2 = 1.0, avg. ppm 6.25; L3 = 1.12, 5.68 ppm; L1 = 0.81, 7.36 ppm; D = 6.36, 8.35 ppm; T1 = 4.99, 2.1 ppm; T = 4.82, 6.76 ppm; T_{avg} = 4.91.

$$Degree\ of\ Branching\ (DB)$$

$$DB = \frac{\#\ of\ dendritic\ units + \#\ of\ terminal\ units}{Total\ \#\ of\ units}$$

$$DB = \frac{D + T}{D + T + L}$$

$$DB = \frac{6.24 + 4.82}{6.24 + 4.82 + 1}$$

$$DB = 0.92$$

$$Conversion\ (Conv.) = \frac{Polymer}{Polymer + Monomer}$$

$$Conv. = \frac{D + T + L}{D + T + L + Monomer}$$

$$Conv. = \frac{6.24 + 4.82 + 1}{6.24 + 4.82 + 1.0 + 4.91}$$

$$Conv. = \frac{12.06}{12.06 + 4.91} = 71\%$$

Hyperbranched PFPE-PEG

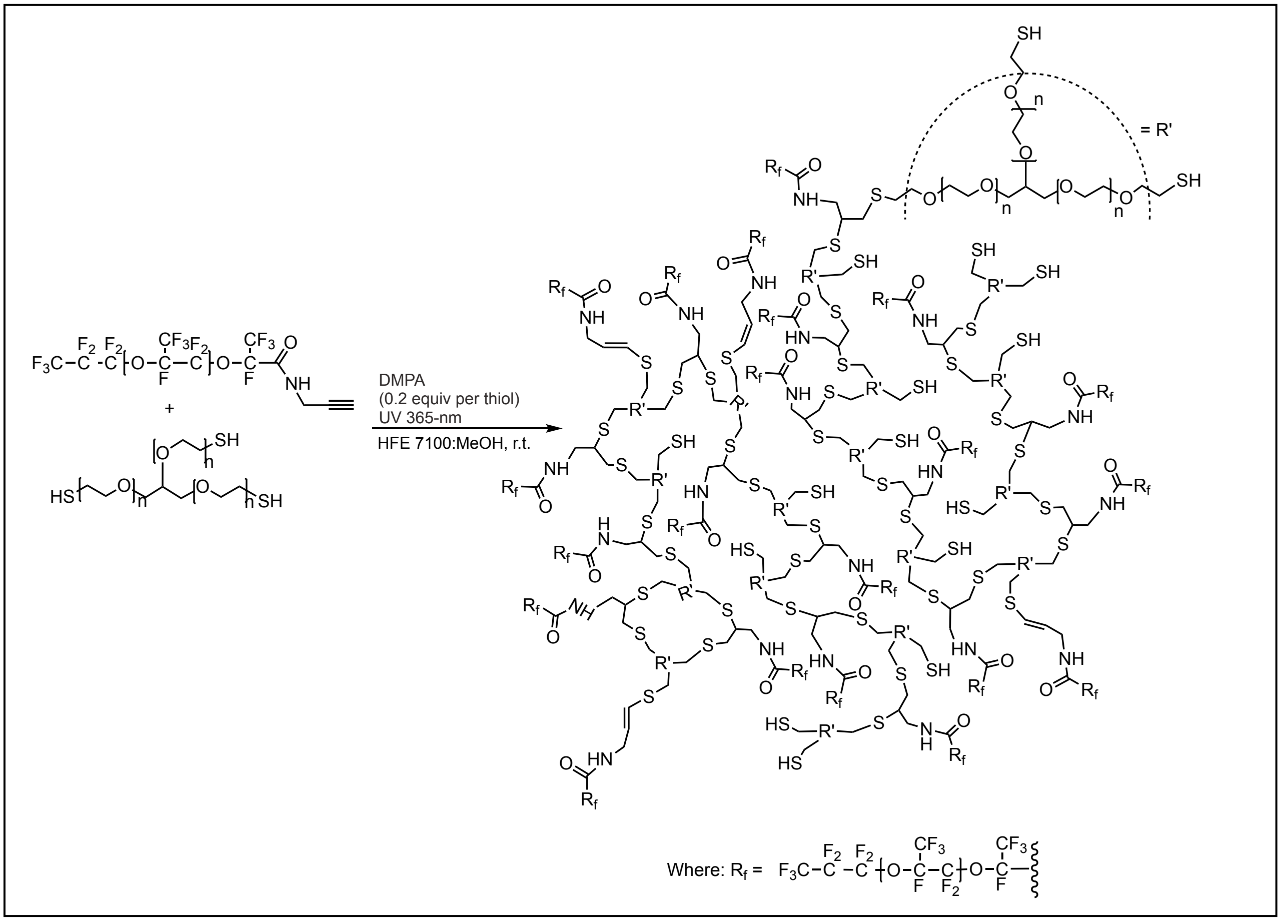

Reagent Table:

Name	MW	Moles/Equiv.	Amount used	Other (density, purity, safety, misc.)
Poly(HFPO) propargyl derivative	5900	0.339-mmol (2 eq)	2.0 g	Yield 1.016
Methanol	-	-<br>-	3.5-mL	bp 64.7 °C, ρ = 0.791 g/mL
HFE 7100 (Methyl nonafluorobutyl ether)	250	-<br>-	7.0-mL	bp 61 °C, ρ = 1.5305 g/mL
2,2-Dimethoxy-2-phenylacetophenone	256.3	0.0678 mmol<br>0.2-eq *	17.4-mg	CAS # 24650-42-8<br>*relative to alkyne groups
Glycerol tthoxylate trithiol	~1100	0.285 mmol (3.78 eq)	313.5 mg	

Experimental Procedure

2.0-g (0.339 mmol, 2 equiv) of PFPE-propargyl derivative was dissolved in 7-mL of methoxyperfluorobutane. Next, 314 mg (0.285 mmol, 3.78 equiv*) of glycerol ethoxylate trithiol and 17.4 mg (0.0678 mmol, 0.2 equiv) of 2,2-Dimethoxy-2-phenylacetophenone DMPA were combined and immediately dissolved in 3.5 mL of MeOH. The DMPA/thiol/MeOH solution was then transferred to the previously generated PFPE-propargyl derivative/HFE 7100 solution and the vial sealed with a septum. The sealed vessel was then thoroughly flushed with N_2 and irradiated at 365-nm under vigorous stirring, at r.t. for ca. 48-hrs. After ca. 48-hrs the crude reaction mixture was combined with an equivalent volume of MeOH (10.5 mL) and the phases were allowed to separate. Finally, the fluorous phase was extracted and evaporated in-vacuo to obtain a clear, colorless oil.

**Stoichiometry adjusted to account for the presence of disulfide.*

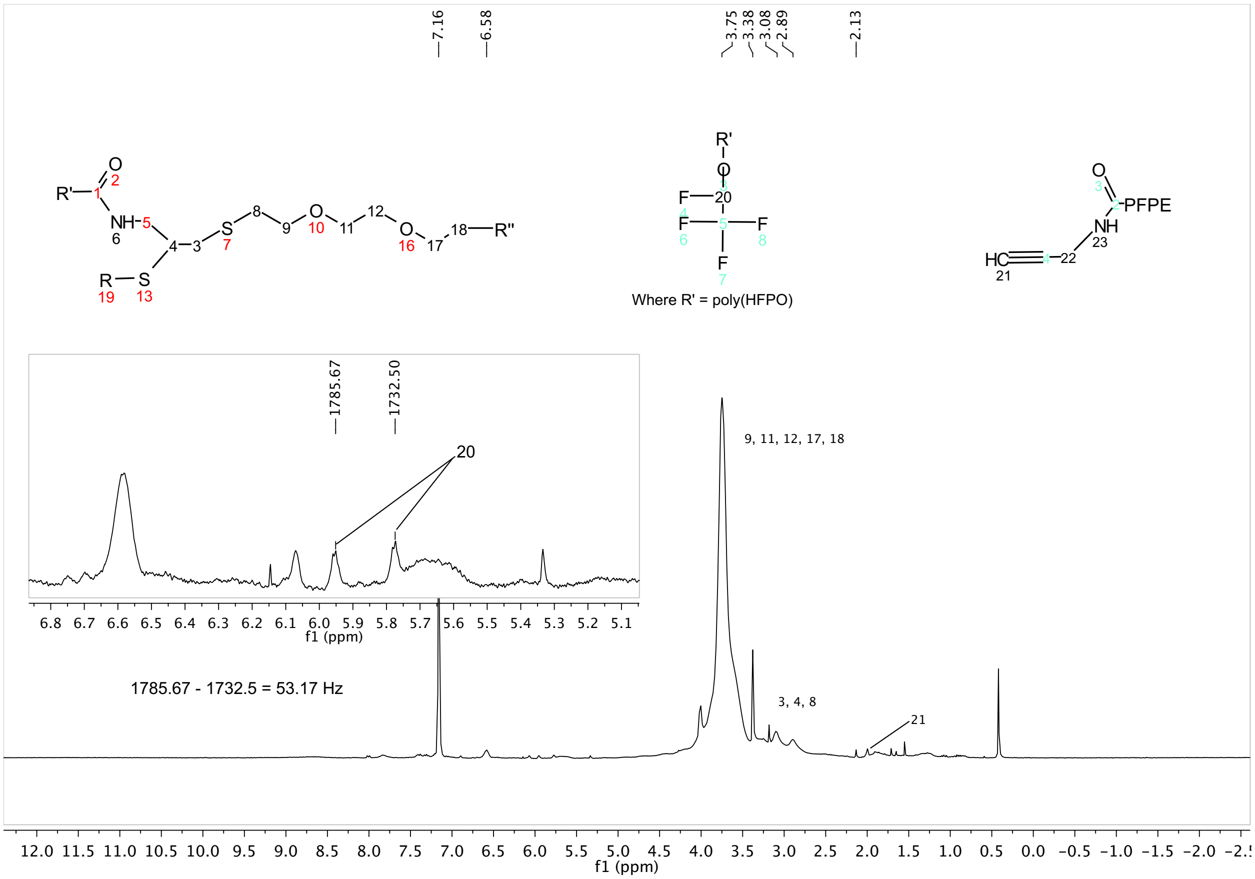

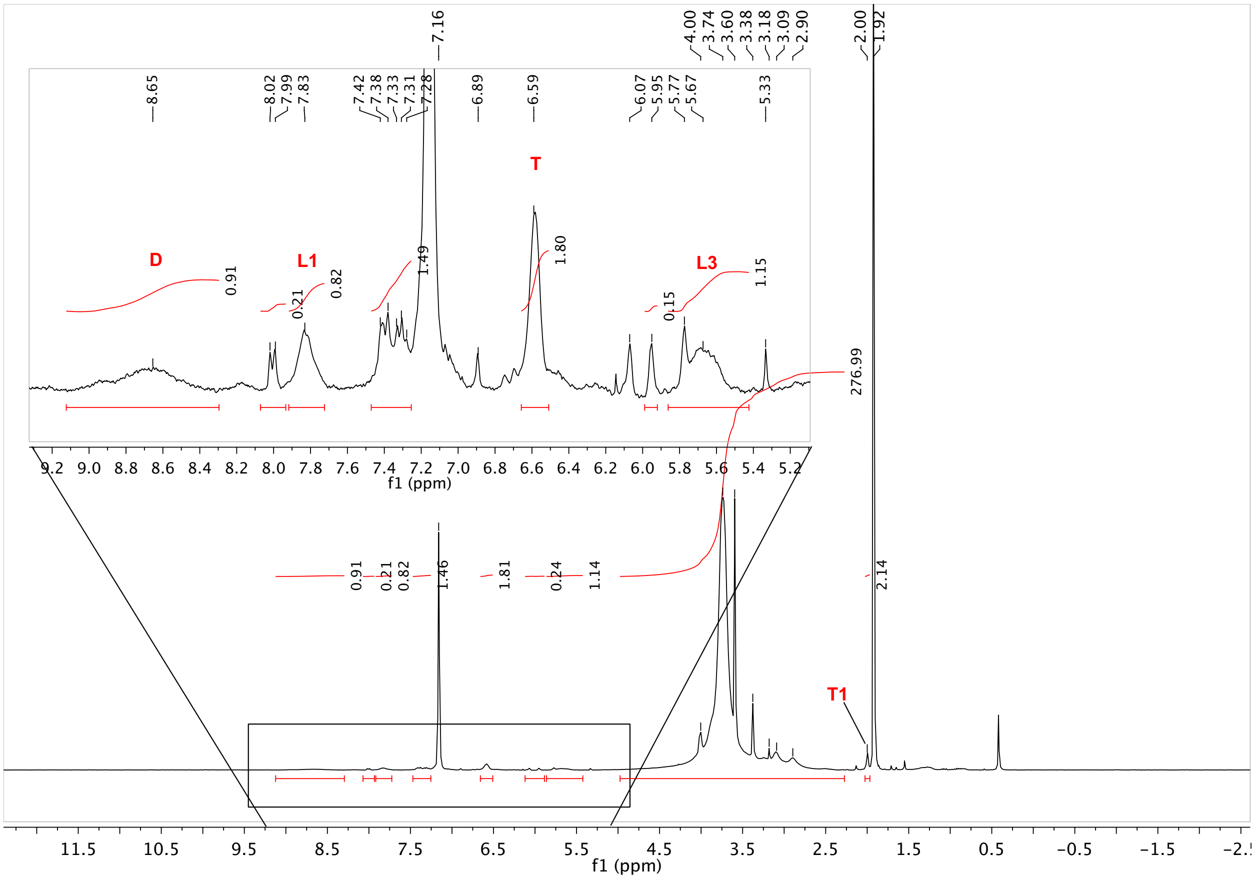

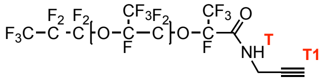

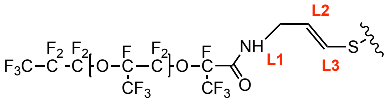

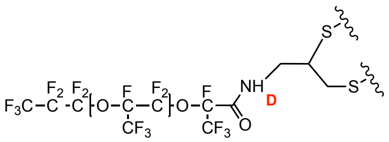

Hyperbranched sulfide linked PFPE-PEG (**15**): Substituting values: L2 = N/A; L3 = 1.0, 5.68 ppm; L1' = 0.82, 7.84 ppm; D = 0.91, 8.68 ppm; T1 = 2.14, 2.0 ppm; T = 1.8, 6.59 ppm; T_{avg} = 1.97.

$$Degree\ of\ Branching\ (DB)$$

$$DB = \frac{\#\ of\ dendritic\ units + \#\ of\ terminal\ units}{Total\ \#\ of\ units}$$

$$DB = \frac{D + T}{D + T + L}$$

$$DB = \frac{0.91 + 1.8}{0.91 + 1.8 + 1}$$

$$DB = 0.74$$

$$Conversion\ (Conv.) = \frac{Polymer}{Polymer + Monomer}$$

$$Conv. = \frac{D + T + L}{D + T + L + Monomer}$$

$$Conv. = \frac{0.91 + 1.8 + 1}{0.91 + 1.8 + 1.0 + 1.97}$$

$$Conv. = \frac{3.71}{3.71 + 1.97} = 65\%$$

PFPE-PEG triazole linked tetra-block

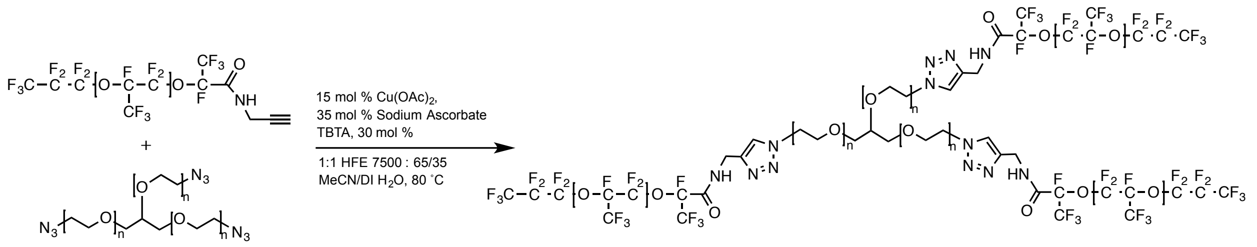

Reagent Table:

Name	FW	Moles/Equiv.	Amount used	Other (density, purity, safety, misc.)
PFPE propargyl derivative	5900	0.254 mmol (3 eq)	1.50 g	Yield = 0.891
Glycerol ethoxylate triazide	1075	0.0889 mmol (1.05 eq)	95.6-mg	
Water	18.02	- - -	0.525-mL	
Methanol	-	-	3.0-mL	bp 64.7 °C, ρ = 0.791 g/mL
Acetonitrile	41.05	---	0.975-mL*	CAS Number 75-05-8, bp 81-82°C (lit.)<br>*Total volume including $Cu(OAc)_2$ stock solution.
(+)-Sodium L-ascorbate	198.11	35 mol % (0.0889 mmol)	17.6-mg	mp 220 °C
HFE 7500	---	- -	1.50-mL	
Copper(II) acetate monohydrate	199.65	0.0381 mmol 15-mol %*	0.00761-g	CAS # 6046-93-1,<br>*with respect to alkyne units
TBTA	530.63	30 mol % (0.0768 mmol)	40.8-mg	

<u>Experimental Procedure</u>:

A solution of 1.5-mL 2-(Trifluoromethyl)-3-ethoxydodecafluorohexane (HFE 7500) and 1.5 g (0.254 mmol. 3.0 eq) of PFPE-propargyl derivative was prepared. 95.6-mg (0.0889 mmol, 1.05 eq) of glycerol ethoxylate triazide and 0.975-mL of MeCN was prepared and sonicated. Next, 0.00761 g (0.0381 mmol, 15 mol %) $Cu(OAc)_2$ and 40.8-mg of tris-(benzyltriazolylmethyl)amine (TBTA) (0.0815 mmol, 16 mol %) were added to the previously generated glycerol ethoxylate triazide/MeCN solution and sonicated. 17.6 mg (0.0889 mmol) of sodium ascorbate was then combined with 0.525 mL of DI H_2O, mixed and transferred to the glycerol ethoxylate triazide/$Cu(OAc)_2$/TBTA/MeCN solution.

Finally, the combined solution was added to the PFPE-propargyl derivative/HFE 7500 solution in a single portion, and stirred at 1200-rpm with a 1” x ½” PTFE coated stir bar at r.t. for ca. 1 hr before heating to 80 °C. After ca. 48 hr, 6.0-mL of MeOH was added to the crude reaction mixture and further stirred for ca. 5 min. Stirring was discontinued and the destabilized emulsion was left to phase separate. The fluorocarbon phase was then isolated and dried over $MgSO_4$ under stirring for ca. 30 min before filtering through a 0.2 μm PFTE syringe filter. Finally, solvent was removed under reduced pressure at elevated temperature (70-80 °C).

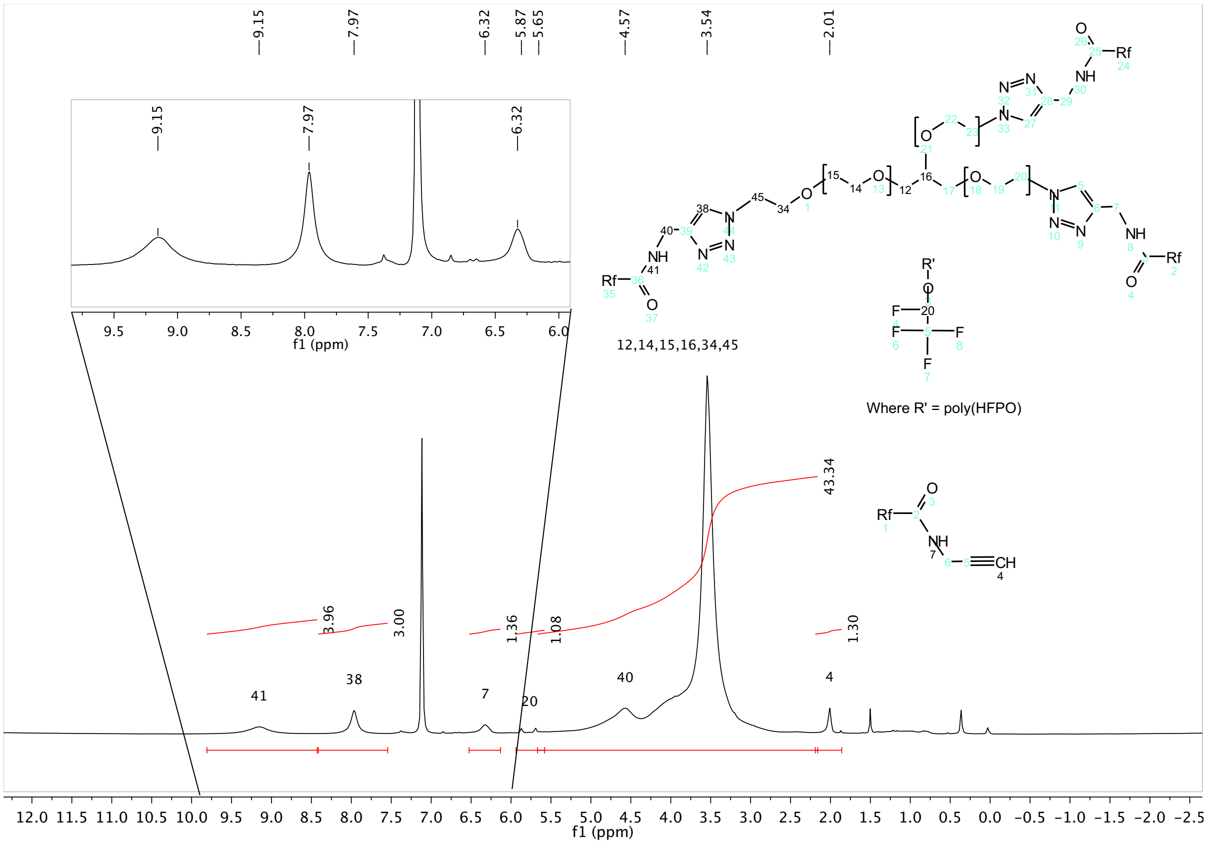

^{1}H NMR (300 MHz, Benzene): 9.15, 7.97, 4.59, 3.55.

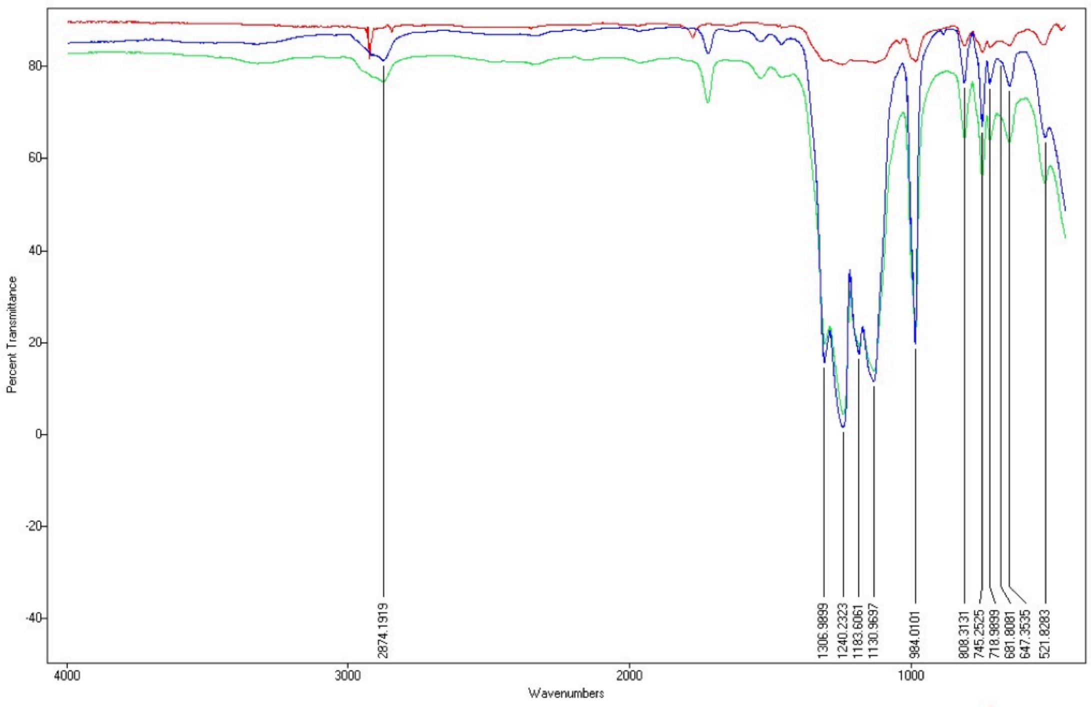

Key:

Red: Krytox FSH carboxylic acid

Green: CuAAC triblock

Blue: Brush-like Thiol-yne

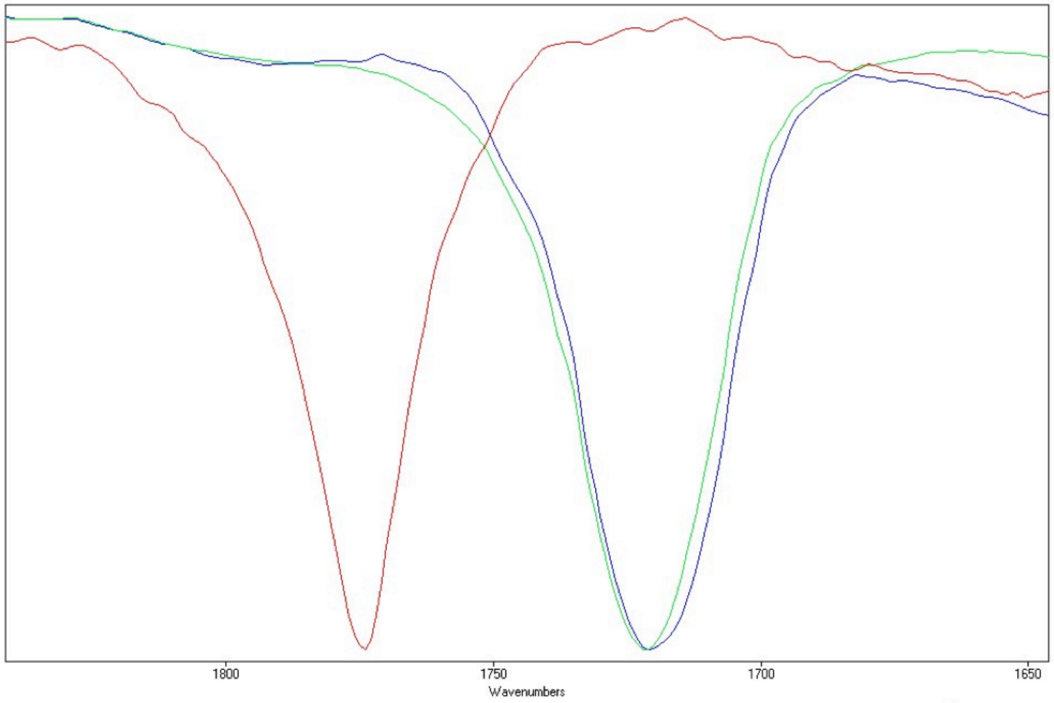

Red: Krytox FSH carboxylic acid C=O stretch 1774.1111 cm^{-1}; Green: CuAAC triblock amide C=O stretch 1721.3999 cm^{-1}; Blue: Thiol-yne brush C=O stretch 1720.8687 cm^{-1}

Supplemental Information 2: NMR characterization of the PFPE-PEG surfactant synthesis

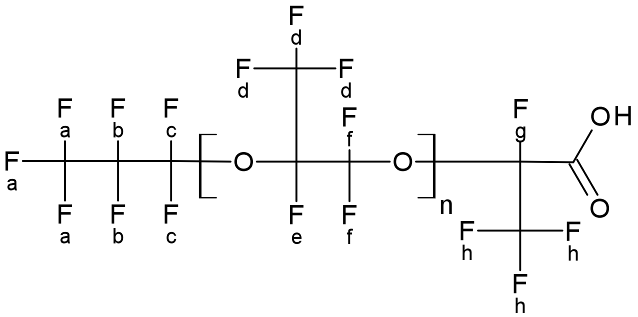

^{19}F NMR Krytox 15(7) FSH (lot K0841): *Poly(Hexafluoropropylene oxide) carboxylic acid*

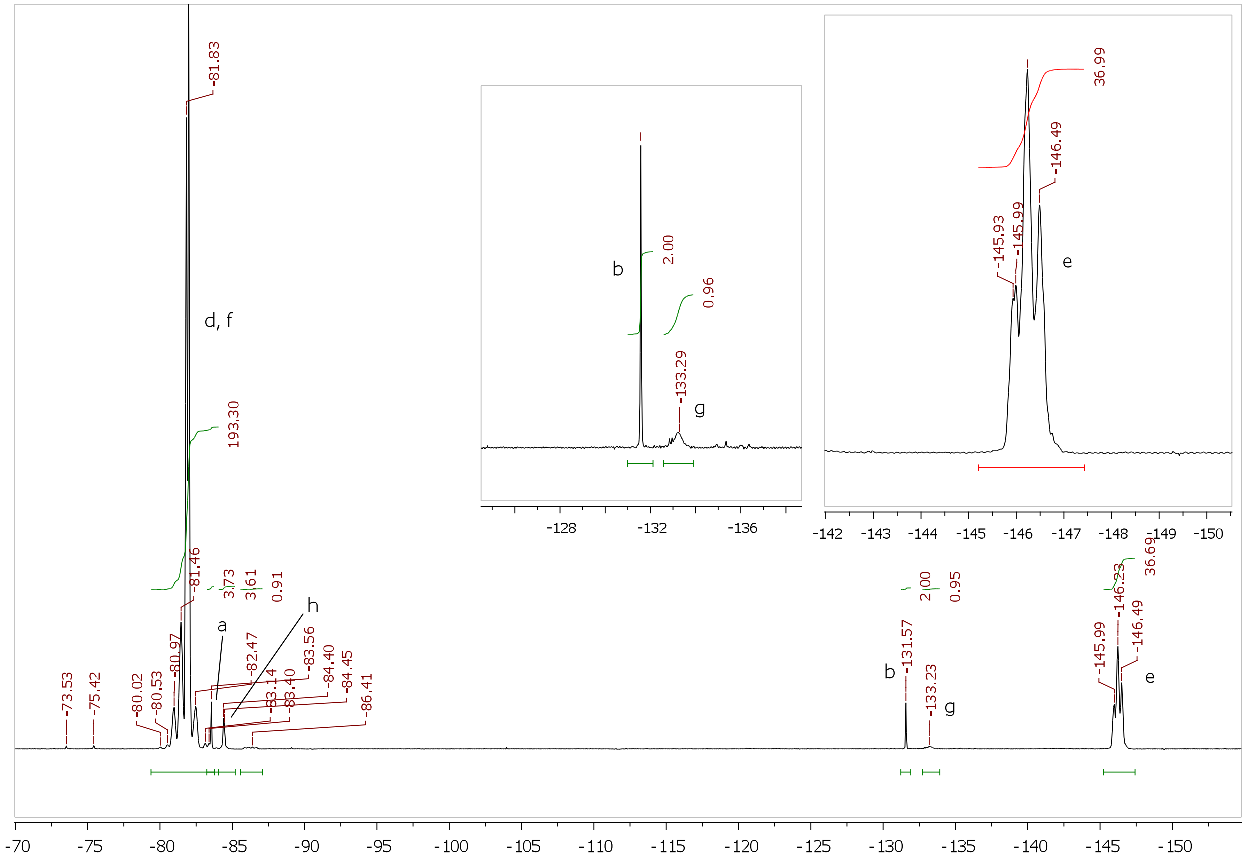

Krytox FSH (Lot K0841) M_n determination: 36.99 repeats, 36.99 repeats x 166 g/mol/repeat = 6140 g/mol; 6140 g/mol + 169 g/mol *(propyl tail)* + 161 g/mol *(carboxylic acid terminus)* = 6470 g/mol; M_n = 6470 g/mol *(assuming three significant figures)*

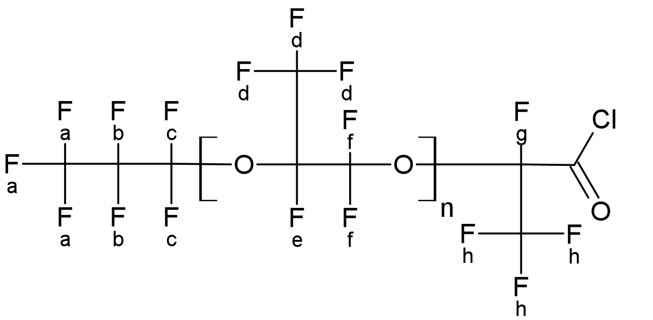

^{19}F NMR *Krytox PFPE acid chloride*

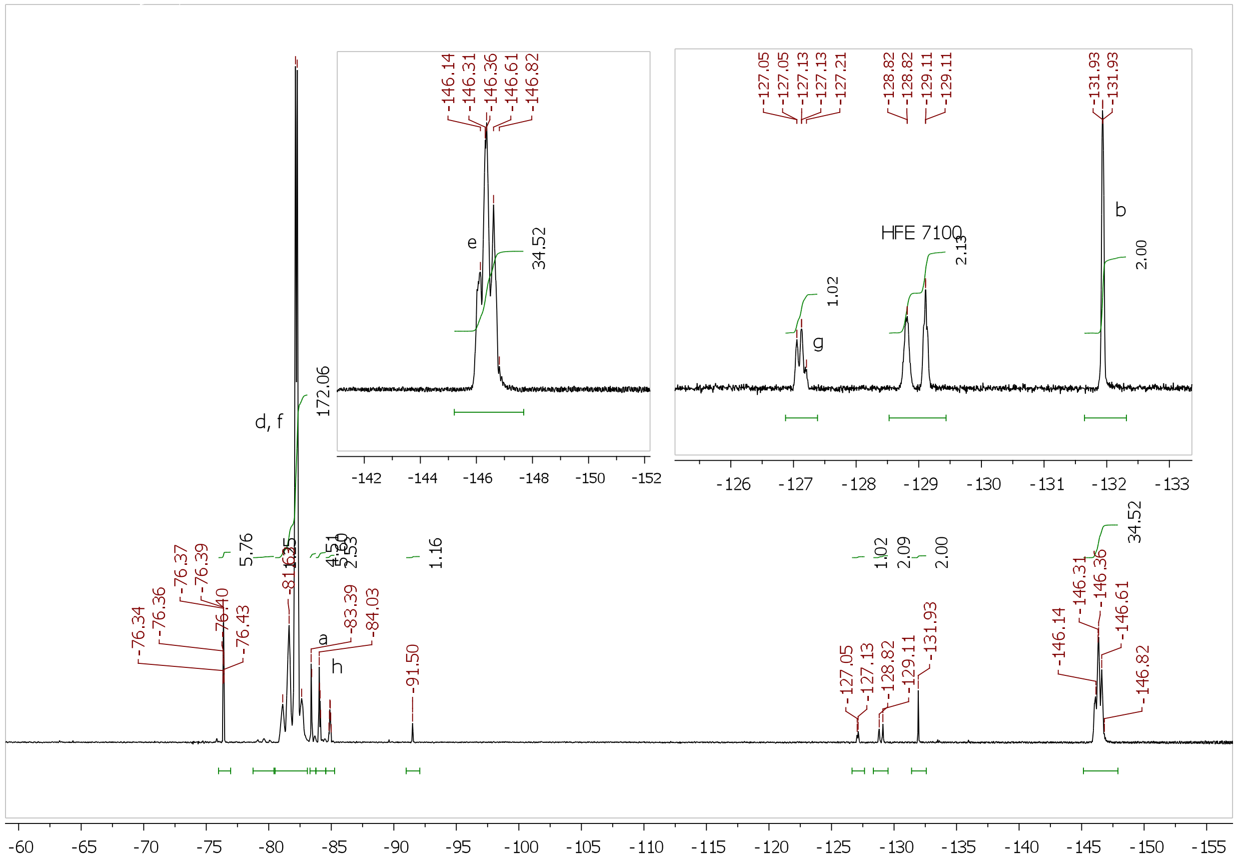

Kytox FSH PFPE acid chloride (Lot RAS-018) M_n determination: 34.52 repeats, 34.52 repeats x 166 g/molrepeat = 5730 g/mol; 5730 g/mol + 169 g/mol *(propyl tail)* + 179.45 g/mol *(carboxylic acid terminus)* = 6078.45 g/mol; M_n = 6080 g/mol *(assuming three significant figures)*

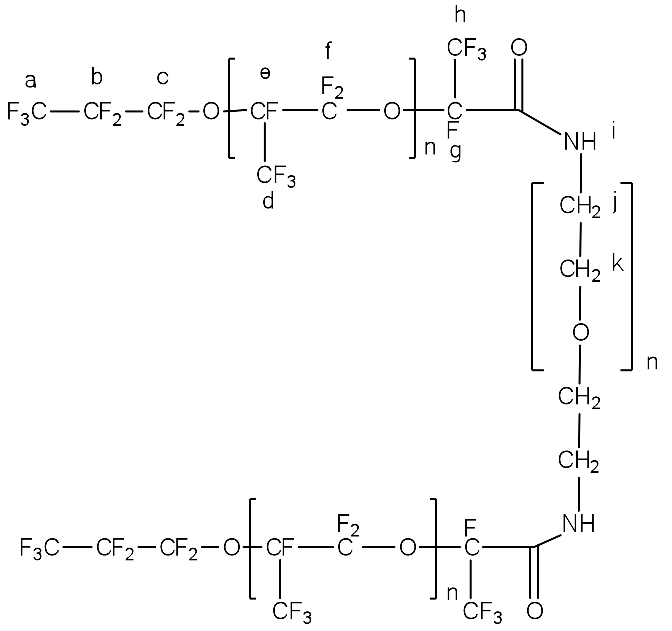

^{19}F NMR *Krytox PFPE/PEG 600 diamide*

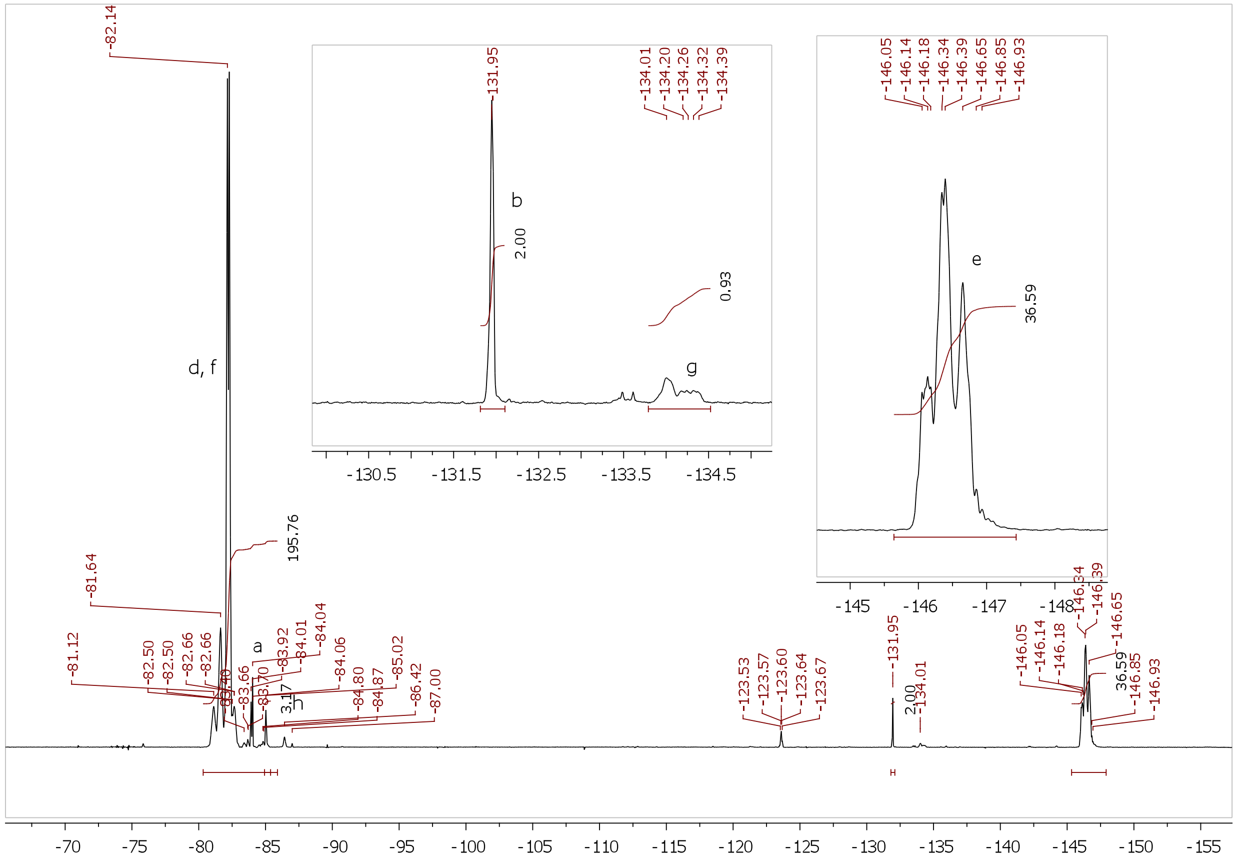

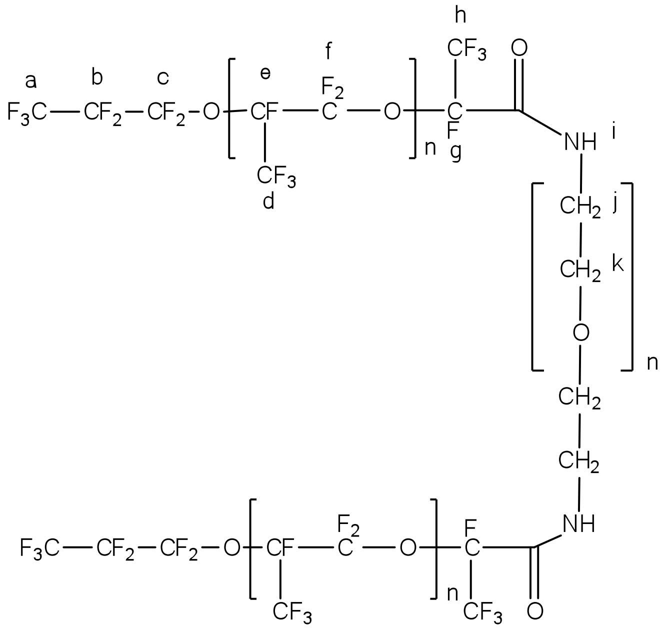

^{1}H NMR *Krytox PFPE/PEG 600 diamide*

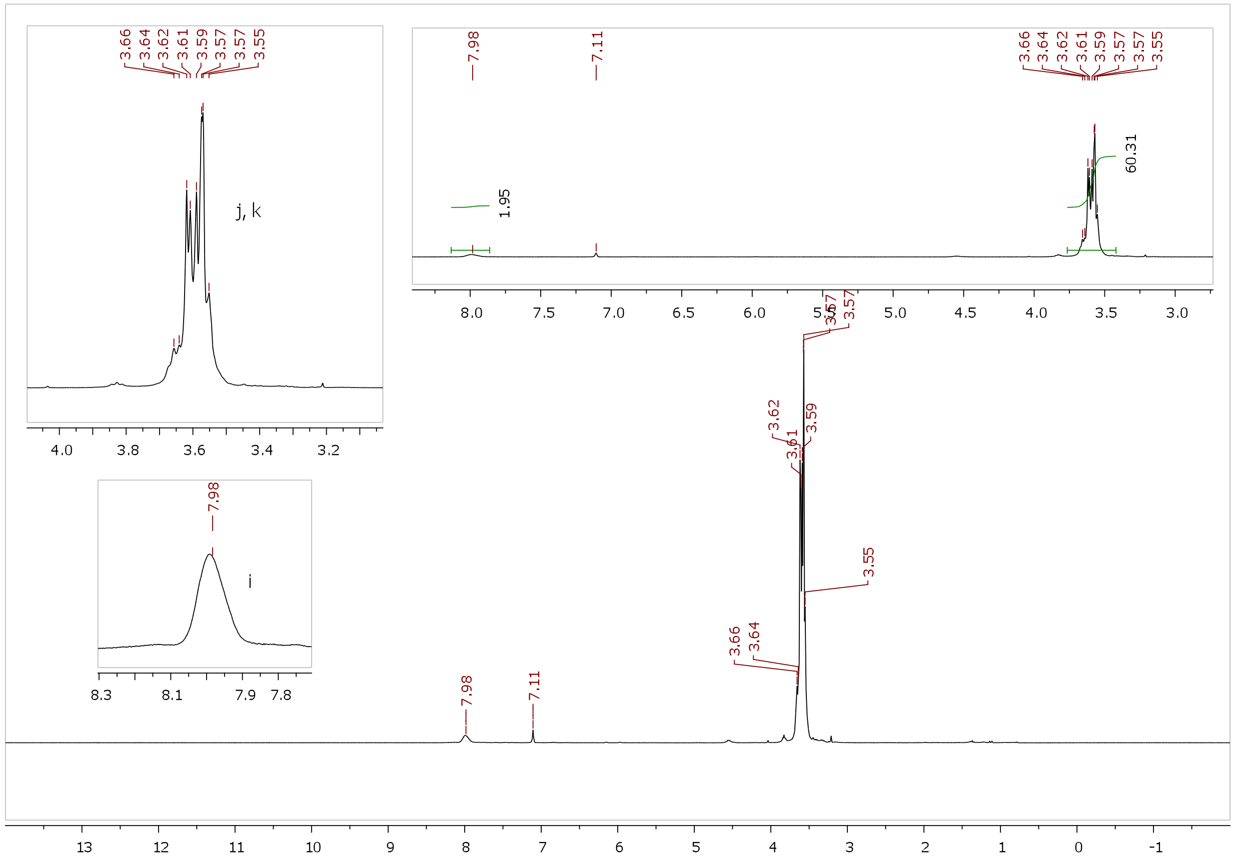

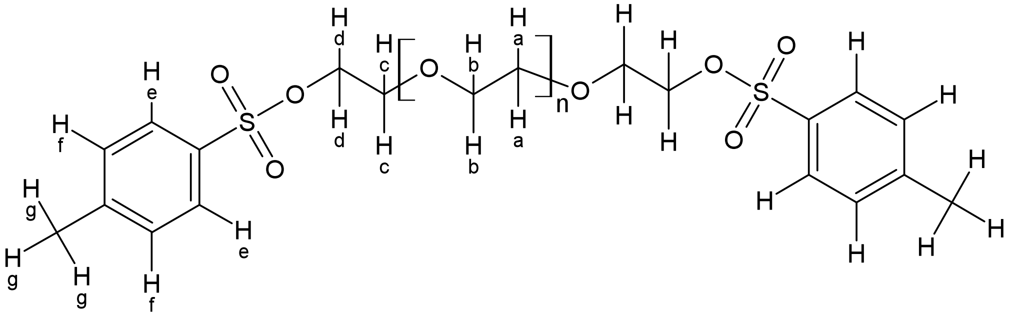

^{1}H NMR *PEG 600 ditosylate*

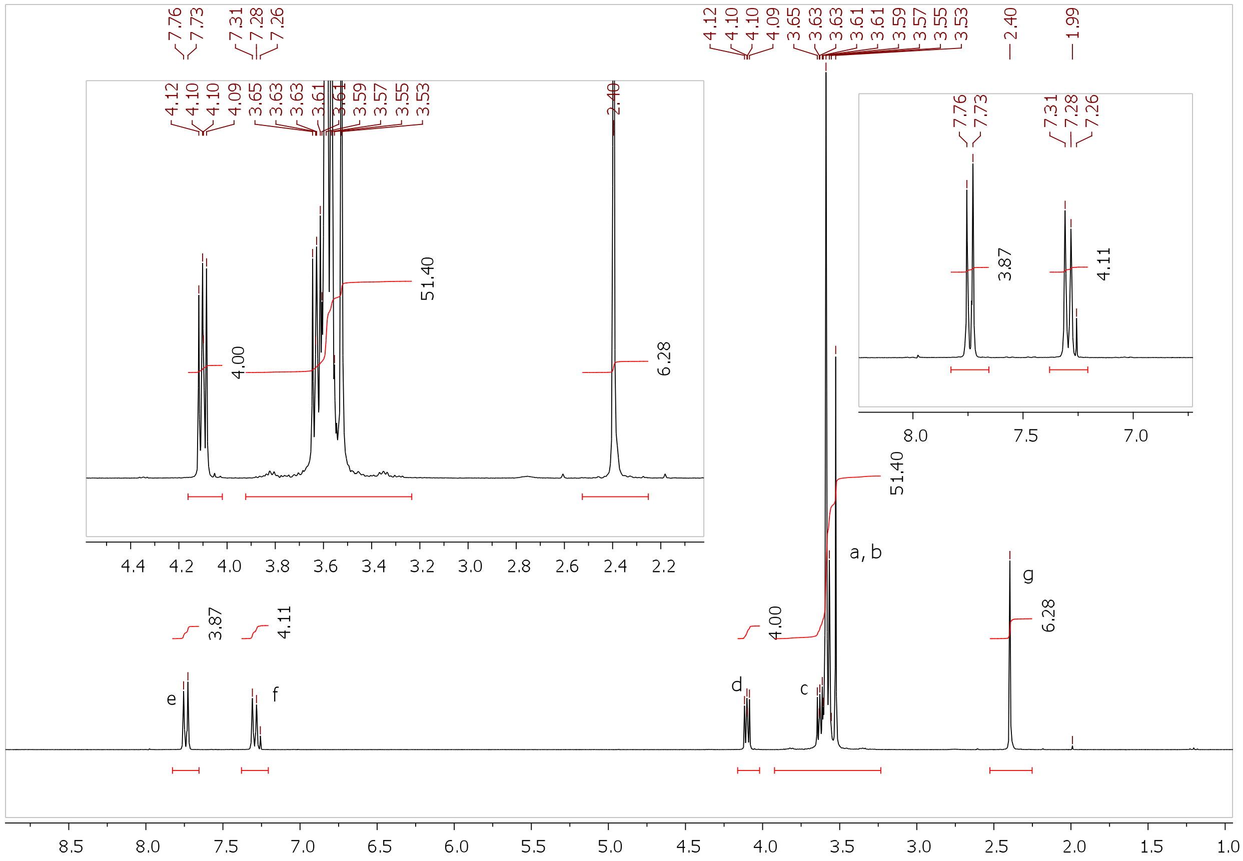

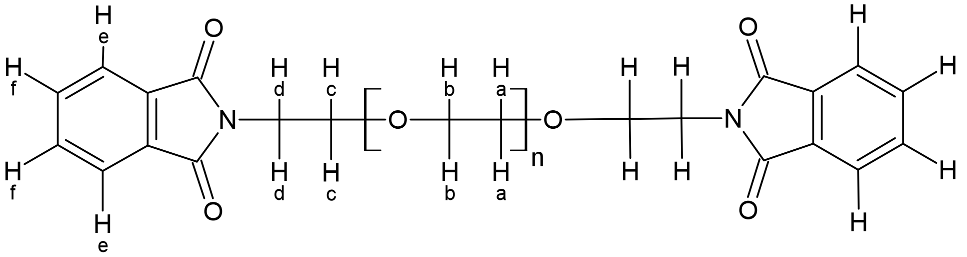

¹H NMR *PEG 600 diphthalimide*

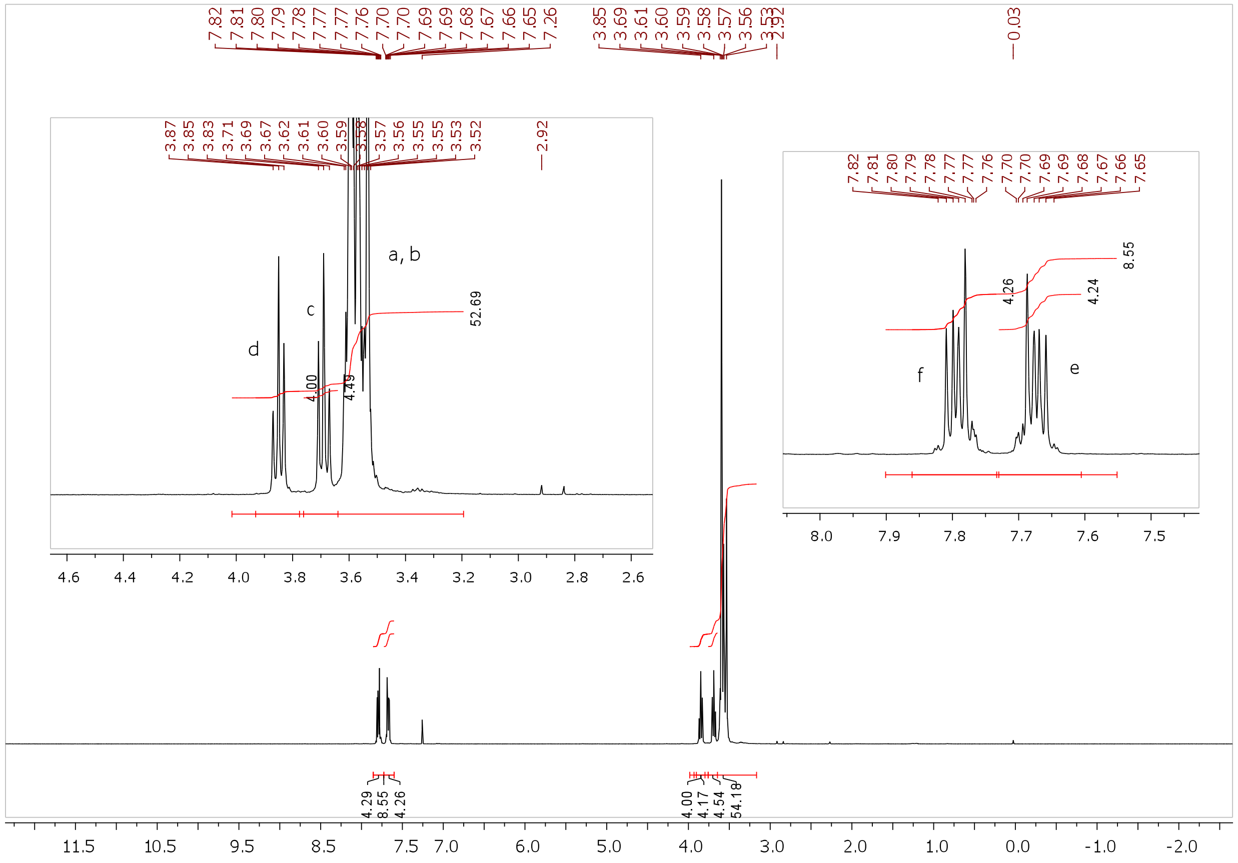

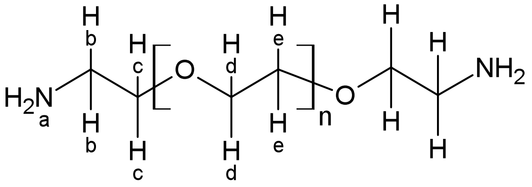

^{1}***H NMR*** *PEG 600 diamine*

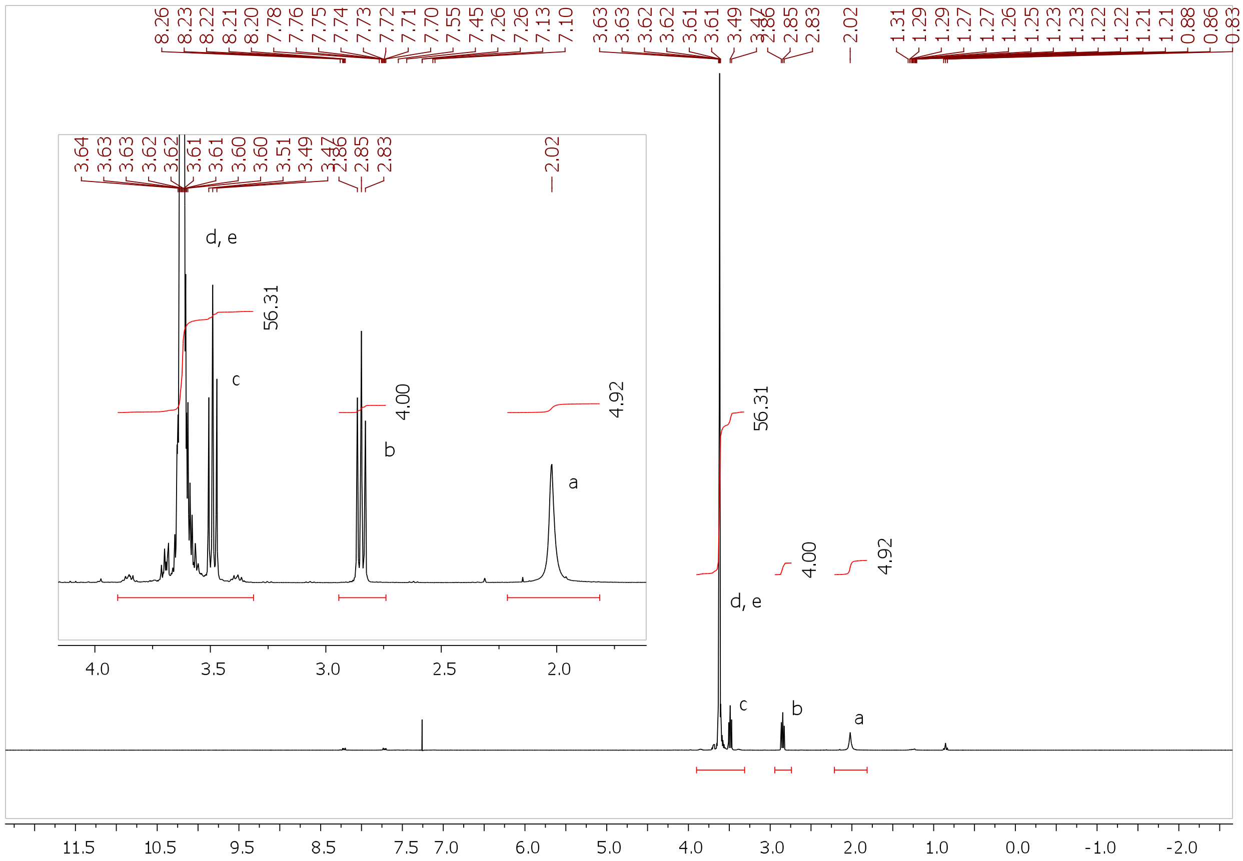

Supplement: RA-008-C8RA01254G-s001 [file RA-008-C8RA01254G-s001.pdf]
